# Challenges in traumatic spinal cord injury care in developing countries – a scoping review

**DOI:** 10.3389/fpubh.2024.1377513

**Published:** 2024-08-19

**Authors:** Mohammad Hosein Ranjbar Hameghavandi, Elaheh Khodadoust, Mahgol Sadat Hassan Zadeh Tabatabaei, Farzin Farahbakhsh, Zahra Ghodsi, Sabra Rostamkhani, Shahryar Ghashghaie, Mahkame Abbaszade, Arash Arbabi, Seyedeh Maede Hossieni, Mohsen Sadeghi-Naini, Rasha Atlasi, Samuel Berchi Kankam, Alexander R. Vaccaro, James Guest, Michael Fehlings, Vafa Rahimi-Movaghar

**Affiliations:** ^1^Sina Trauma and Surgery Research Center, Tehran University of Medical Sciences, Tehran, Iran; ^2^Neurosurgery Department, Shohada Hospital, Lorestan University of medical sciences, Khoram-Abad, Iran; ^3^Non-Communicable Diseases Research Center, Endocrinology and Metabolism Population Sciences Institute, Tehran, Iran; ^4^Endocrinology and Metabolism Research Center, Endocrinology and Metabolism Clinical Sciences Institute, Tehran University of Medical Sciences, Tehran, Iran; ^5^Department of Neurosurgery, Shariati Hospital, Tehran University of Medical Sciences, Tehran, Iran; ^6^Department of Orthopedics and Neurosurgery, Rothman Institute, Thomas Jefferson University, Philadelphia, PA, United States; ^7^Neurosurgery and the Miami Project to Cure Paralysis, Miller School of Medicine, University of Miami, Coral Gables, FL, United States; ^8^Division of Genetics and Development, Krembil Research Institute, University Health Network, Toronto, ON, Canada; ^9^Division of Neurosurgery, Toronto Western Hospital, University Health Network, Toronto, ON, Canada; ^10^Department of Surgery and Spine Program, University of Toronto, Toronto, ON, Canada; ^11^Universal Scientific Education and Research Network (USERN), Tehran, Iran; ^12^Spine Program, University of Toronto, Toronto, ON, Canada

**Keywords:** spinal cord injury, patient care management, developing countries, wounds and injuries, prevention and control

## Abstract

**Objective:**

To evaluate the leading challenges in developing countries’ traumatic spinal cord injury (TSCI) care.

**Methods:**

We conducted a systematic search in electronic databases of PubMed, SCOPUS, Web of Science, EMBASE, and Cochrane Library on 16 April 2023. Studies that investigated challenges associated with the management of TSCI in developing countries were eligible for review. We extracted related outcomes and categorized them into four distinct parts: injury prevention, pre-hospital care, in-hospital care, and post-hospital care.

**Results:**

We identified 82 articles that met the eligibility criteria including 13 studies on injury prevention, 25 on pre-hospital care, 32 on in-hospital care, and 61 on post-hospital care. Challenges related to post-hospital problems including the personal, financial, and social consequences of patients’ disabilities and the deficiencies in empowering people with TSCI were foremost studied. Lack of trained human resources, insufficient public education and delays in care delivery were barriers in the acute and chronic management of TSCI. A well-defined pre-hospital network and standard guidelines for the management of acute neurotrauma are needed. Critical challenges in injury prevention include deficiencies in infrastructure and supportive legislation.

**Conclusion:**

Studies focusing on injury prevention and pre-hospital care in TSCI management in developing countries warrant further investigation. It is imperative to develop systematic and evidence-based initiatives that are specifically tailored to the unique circumstances of each country to address these challenges effectively. By understanding the primary obstacles, policymakers and healthcare providers can establish goals for improving education, planning, legislation, and resource allocation.

## 1 Introduction

Traumatic Spinal Cord Injury (TSCI) is a devastating condition with a global incidence of 0.9 million cases annually (1). Developing countries have an annual incidence of 22.55 cases per million (2), while the provision of care for TSCI is comparatively less organized and effective when compared to developed countries (3). There are notable discrepancies in the management of TSCI between high-income and low- and middle-income countries, particularly regarding the availability and organization of trauma care services, rehabilitation facilities, and access to medical resources (4). The social support and health system are crucially associated with the health-related outcomes of individuals with long-term disabilities and the region of living is a strong predictor of these measures (5). TSCI imposes a significant economic burden on both the healthcare system and individuals with disabilities (6). Moreover, about two million deaths due to trauma are preventable through improving the trauma care system in developing countries (7, 8) and significant disparities in TSCI-related mortality rates exist between developed and developing countries. These differences are partly due to social, economic, political, and geographical obstacles in trauma care and prevention (3, 9). For instance, Iran’s road traffic crash (RTC) mortality rates are twice the rates observed in Europe and North America (10).

To better understand the challenges faced by policymakers in developing countries, it is essential to classify the care provided for TSCI based on the temporal characteristics of accident avoidance and management. This classification covers several key aspects, including injury prevention, pre-hospital communication, triage, and transport, as well as in-hospital and post-hospital care.

Challenges encompass a broad range of issues that collectively hinder the effective prevention, management, and rehabilitation of TSCI in developing countries. These challenges include economic, socio-cultural, and political challenges. However, the scope of challenges goes beyond these categories, reflecting the multifaceted nature of addressing TSCI in developing countries. Various components of healthcare systems and government entities face distinct challenges and require specific solutions within each category. For instance, in a review conducted in LMICs, it was discovered that inadequate access to cervical collars for immobilization was a prevalent issue (11). Furthermore, the lack of efficient and expedited transport emerged as the most critical pre-hospital factor contributing to mortality rates (11). Social media platforms, preventive legislation, and road safety regulations play crucial roles in injury prevention (4). However, healthcare systems can also contribute to enhancing the quality of care during both the pre-hospital and in-hospital phases. Zakrasek et al. identified several areas that require intervention, including acute care, support surfaces, nurse-to-patient ratios, and education (12). Cultural perspectives can also impose limitations on post-hospital care, particularly when injured patients are faced with obstacles that prevent them from reintegrating into their communities due to their acquired disabilities (13).

The initial step in problem-solving involves recognition and defining the issues. Drawing from Kingdon’s multiple stream theory, it is essential to transform a health concern into a well-defined problem and address various aspects of that problem when formulating health priorities in policy-making (14). Despite the significant mortality and morbidity attributed to TSCI, there is a lack of studies systematically reviewing and classifying challenges related to TSCI prevention and care within the health systems of developing countries. In the present study, we aimed to comprehensively identify challenges related to TSCI prevention, providing appropriate pre-hospital, in-hospital and post-hospital trauma care and support in developing countries. Our goal is to enable policymakers to enhance TSCI care across primary, secondary, and tertiary prevention levels. The diverse and wide-ranging outcomes from existing studies prompted the need for this scoping review.

## 2 Methods

We have conducted a scoping review which is a suitable method for data collection and synthesis in the broader topics with various outcomes (15). This scoping review is based on the Preferred Reporting Items for Systematic Reviews and Meta-Analyses (PRISMA) reporting framework for the scoping review. We used the PICo (Population, phenomena of Interest, Context) model to formulate the question of this qualitative scoping review (16). Our research question is: What are the challenges in the management (I) of TSCI (P) from prevention to post-hospital care in developing countries (Co)?

### 2.1 Identifying related studies

A systematic search was conducted in electronic databases of PubMed, SCOPUS, Web of Science, and the EMBASE and Cochrane Library on 16 April 2023. Our search strategy was formed based on three concepts including “Traumatic Spinal Cord Injury,” “management,” and “developing countries” (Appendix A).

### 2.2 Study selection

After deduplication, two reviewers independently assessed the titles and abstracts. Thereafter, two independent researchers assessed the full text of the eligible studies based on predefined eligibility criteria. Disagreements were resolved by a third reviewer.

### 2.3 Eligibility criteria

We included studies that discussed TSCI management and pertinent challenges in developing countries, without any restrictions based on language, or timeframe. Developing countries were defined based on the International Monetary Fund (IFM) list (17). The term of challenges in our study refers to problems that require effort, strategy and problem-solving to overcome the obstacles. Studies were excluded if the primary study objective was not the challenges posed by TSCI, focused on spinal fracture without TSCI, non-traumatic spinal cord injury, or only Traumatic Brain Injury (TBI). We also excluded case reports, reviews, case series containing less than 10 cases, animal studies, unpublished data, and studies conducted in high-income countries.

### 2.4 Data extraction

We collected general information, study design, and outcomes relating to challenges in managing TSCI in the developing countries in four key areas: injury prevention, pre-hospital triage, in-patient acute management, and post-hospital care (Appendix B). We did not appraise the methodological quality and risk of bias of the included studies, which is aligned with the scoping review guidelines.

## 3 Results

**Figure 1:**
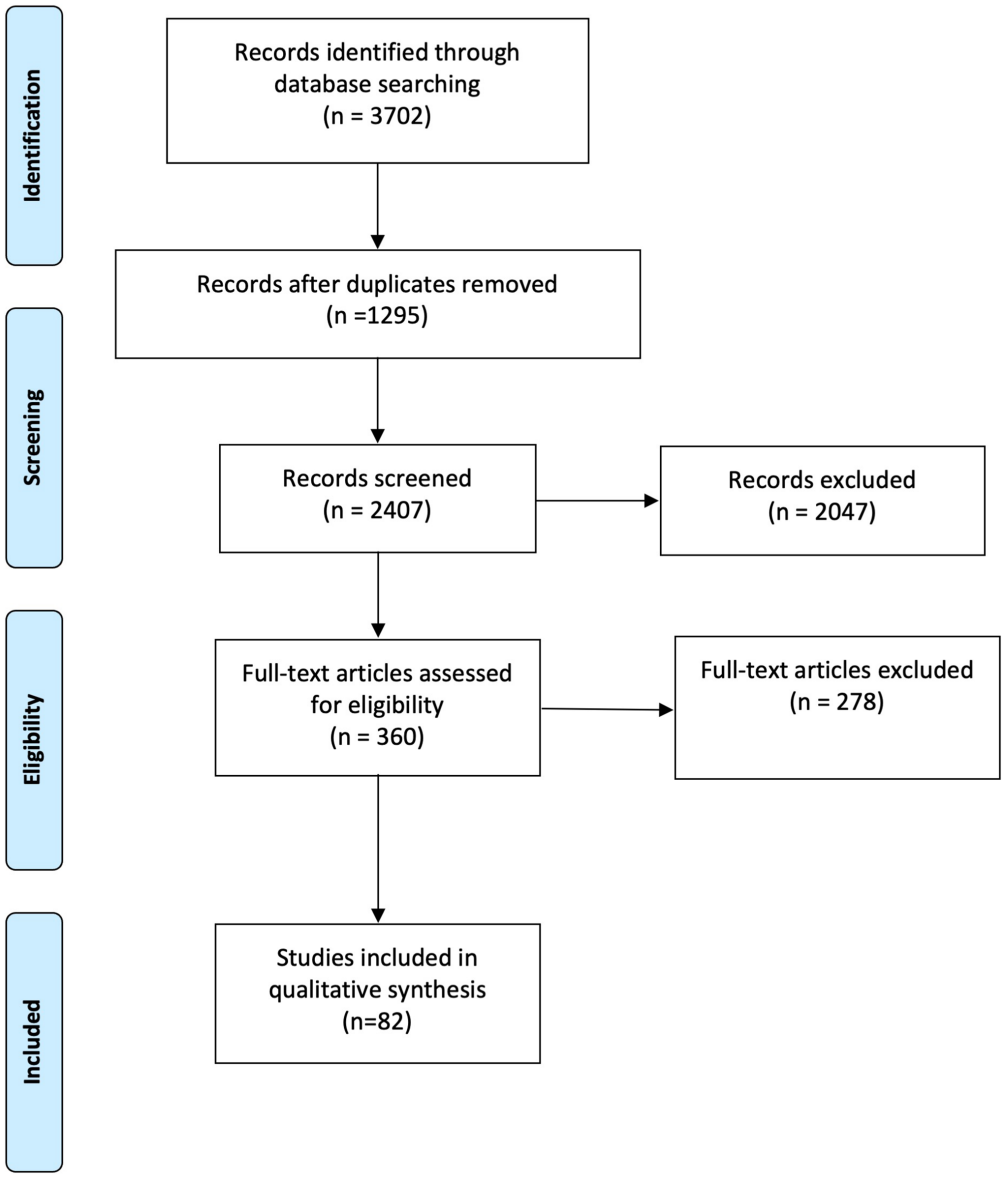
PRISMA flow chart.

We found 1,295 articles after removing duplicates. Following screening titles and abstracts, 360 articles were included for full-text review. A total of 82 articles matched our study goals (10, 18–101), and were included for data analysis. The PRISMA chart is presented in Figure 1 (15). The included studies were published between 1983 and 2023 with 63.4% (*n* = 52) of them published after 2018. These studies described care challenges surrounding TSCI from 26 developing countries. Most articles were from Iran (*n* = 9), India (*n* = 8), Nepal (*n* = 8), and South Africa (*n* = 8). Brazil, Cambodia, Colombia, Ethiopia, Haiti, Macedonia, Malaysia, and the United Arab Emirates were the least studied countries with one article for each. The challenges in the African Region (AFR) were most common in this review (*n* = 30) and the American and European regions had the least number of studies, which can be attributed to the small number of developing countries in these regions (*n* = 3). The South-East Asian Region (SEAR), Eastern Mediterranean Region (EMR), and Western Pacific Region (WPR) were mentioned in 24, 16, and 4 studies, respectively. Two studies were international and multi-center. Fourteen, 25, 32, and 61 articles included injury prevention, pre-hospital, in-hospital, and post-hospital, respectively which are illustrated in Appendix B. We also categorized the neurotrauma challenges for each country which are illustrated in Appendix C.

### 3.1 Injury prevention

**Figure 2:**
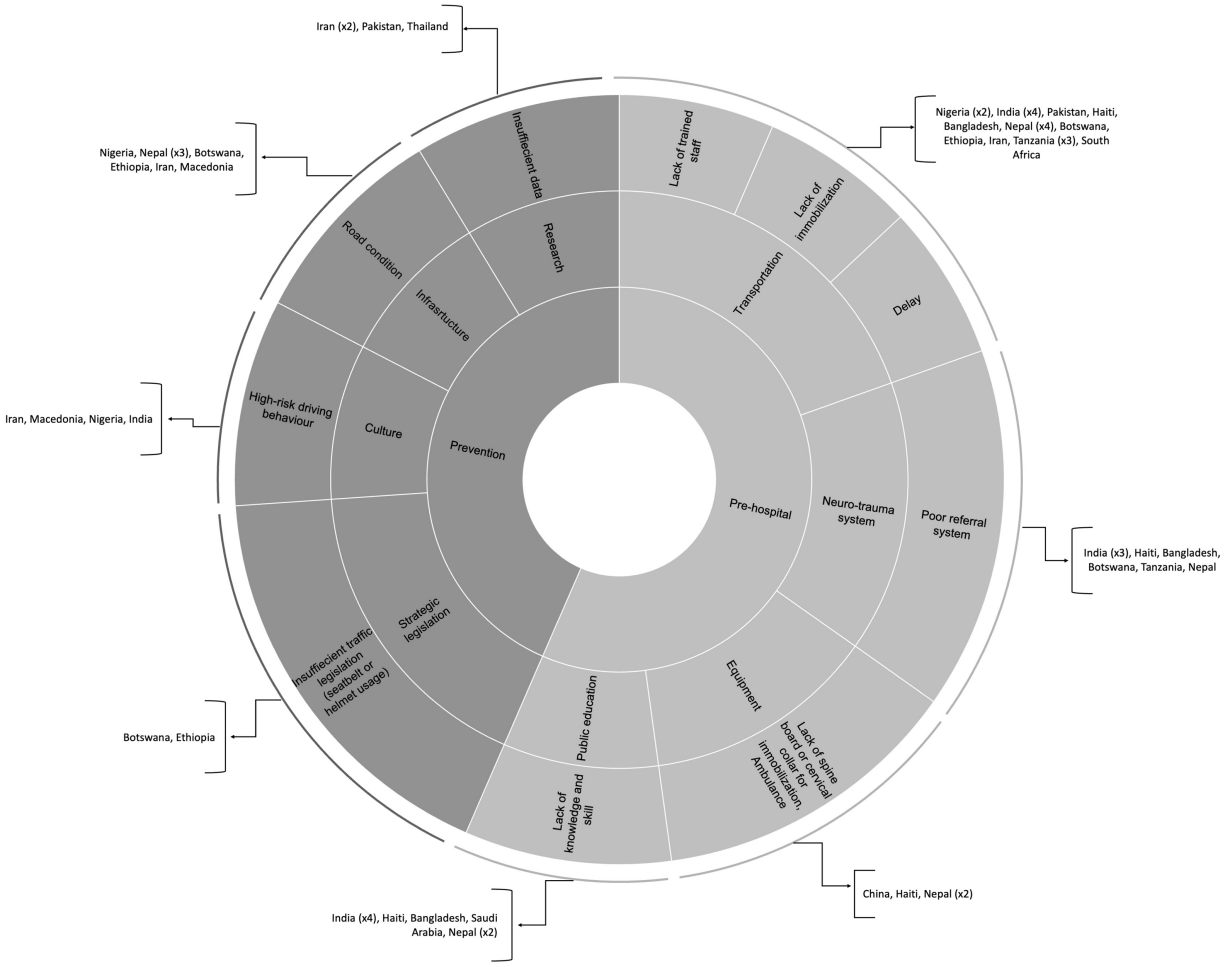
Challenges related to prevention and pre-hospital TSCI management.

The injury prevention category was evaluated by 13 articles (10, 18, 26, 28, 36, 41, 44, 59, 62, 76, 88, 93) (Appendix B-Table S2). We identified four main challenges, including a lack of valid data based on well-defined research, preventive legislation, cultural barriers to implementing preventive strategies, and a lack of well-defined infrastructure to address injury prevention. There were prominent gaps in injury prevention in Iran (10, 26, 102), Nigeria (18, 27, 76), Tanzania (79), and Saudi Arabia (103). Figure 2 illustrates the key challenges in injury prevention. The challenges related to injury prevention can be categorized into four main categories.

#### 3.1.1 Research

Four studies mentioned lack of sufficient data (26), lack of an SCI registry (28), low case identification rate and a delay in data completion at the SCI registry (76), and lack of research on the epidemiology of SCI (88) as some of the challenges that exist with research in the injury prevention phase of SCI management. Azadmanjir et al. outlined insufficient infrastructure, funds, trained human resources, and technical issues as the root causes for poor data gathering through a registry (76).

#### 3.1.2 Stewardship

On the legislative front, the absence of laws mandating seatbelt use in the backseats of motor vehicles is a notable concern (93).

#### 3.1.3 Cultural and behavioral challenges

Inadequate use of restraints and helmets, poor driving behaviors (10), diving into shallow water is one of the reasons for TSCI (51).

#### 3.1.4 Infrastructure

Infra-structure problems were the most cited challenge in the injury prevention phase especially in Nepal and included non-standard roads (51), poor driving (18, 41), single-lane highways with no separation from street lights or domestic animals, poor cars and tires (93), non-standard old motor vehicles (41), inaccessibility to all-weather roads (44), and unsafe workplaces (36).

### 3.2 Pre-hospital

Challenges in pre-hospital care were mentioned in 25 articles (18, 19, 21, 25–29, 32, 33, 36, 41, 44, 48, 50, 53, 56, 59, 63–65, 75, 79, 82, 92–94) (Appendix B-Table S3) including lack of adequate public education, lack of a well-defined pre-hospital system and communication network to manage TSCI, inadequate equipment, transportation difficulties, and inequality in implementing trauma guidelines. Nepal (36, 44, 53, 64, 75), India (19, 25, 59, 92, 94, 104), and Tanzania (63, 79, 82) were the most commonly investigated countries for pre-hospital care. Figure 2 shows the main problems in pre-hospital management in developing countries. These can be examined in five groups.

#### 3.2.1 Public education

Including lack of knowledge about essential precautions for transportation (25), a lack of skilled human resources (29, 75), and a lack of basic knowledge about immobilization (32, 75). Several reports indicated that untrained people helping the injured can lead to or exacerbate an underlying TSCI (18, 19, 21, 25, 28, 29, 32, 33, 36, 41, 48, 92, 93).

#### 3.2.2 Integrated neurotrauma care system

Including insufficient integrated pre-hospital system, communication network, and patient journey and referral centers (29, 79).

#### 3.2.3 Equipment

Lack of special equipment for safe transportation including Emergency Medical Care (EMC) and equipment, EMC with no special equipment for SCI patients (29), and an ambulance shortage due to bureaucracy in importing ambulances from international borders (44). Rathore et al. found that not only were ambulances and high-tech instruments scarce, but simple and efficient tools such as spine boards were also rarely available in Pakistan (28).

#### 3.2.4 Transportation

Transportation by untrained personnel (18, 33, 75, 92) or health care providers’ unawareness of log rolling and immobilization techniques (28, 29, 32, 36, 41, 65), delay in transportation to SCI centers due to poverty and lack of awareness (27, 33, 44, 75, 79, 82, 92), poor referral system (93), or the far distance between the accident site and EMC (21), and lack of ambulance availability (75), more availability of fast unequipped road vehicles in comparison with equipped ambulances (19, 33, 48, 75, 93), and inadequate airlift capabilities (28).

#### 3.2.5 Adherence to guidelines

Two included studies evaluated commitment to standard SCI management guidelines in developing countries. They found that unawareness of the American Spinal Injury Association (ASIA) system for differentiating complete and incomplete SCI (28) and poor national health referral systems (33, 75) may cause inappropriate or incomplete treatment strategies for managing patients with TSCI.

### 3.3 In-hospital

**Figure 3:**
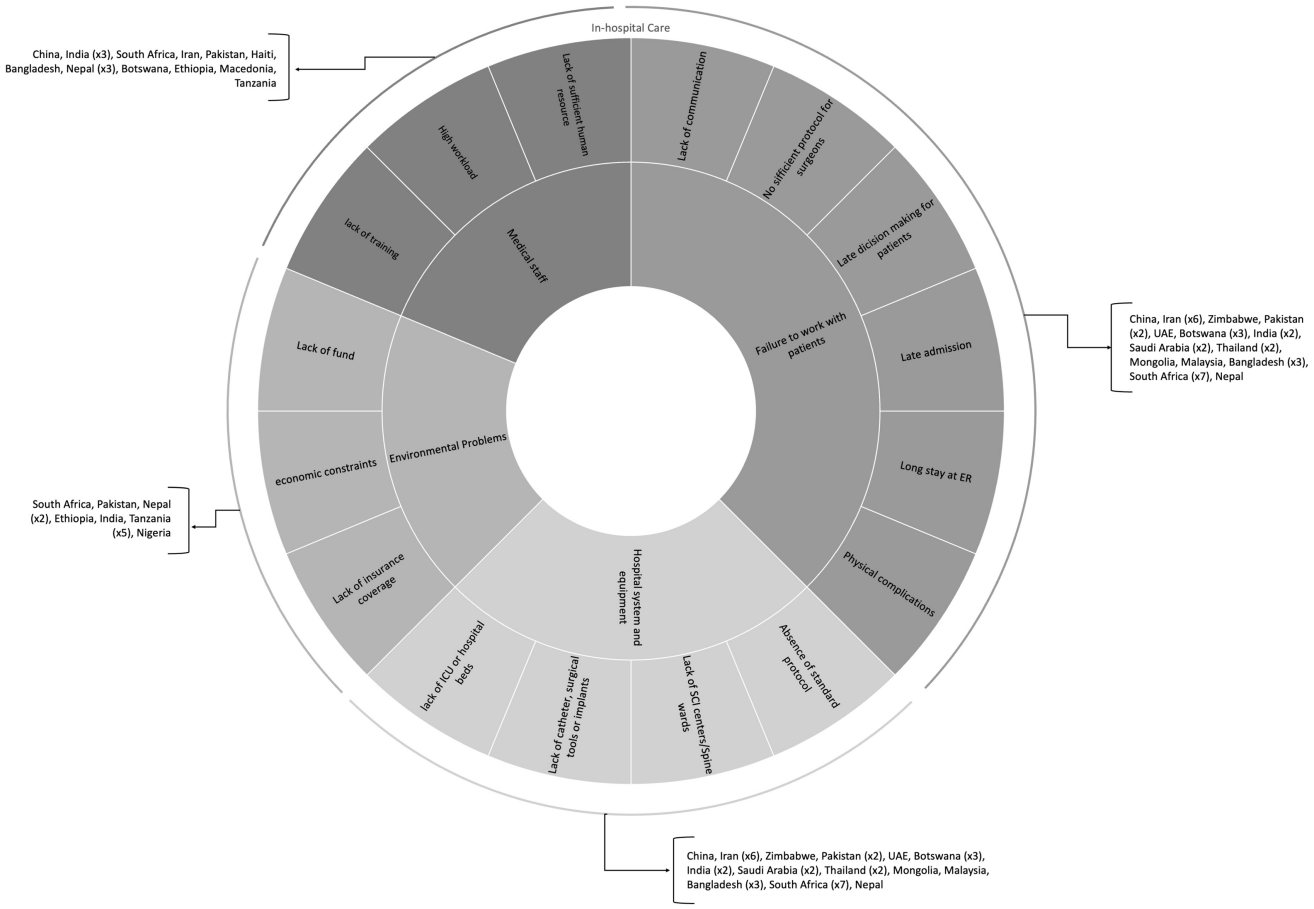
In-hospital TSCI management challenges.

TSCI challenges encountered during hospital admission and acute management were evaluated in 32 articles (18–22, 26, 28, 29, 32, 33, 36, 38, 40–44, 50–52, 59, 61–65, 79, 80, 82, 92–94) (Appendix B-Table S4) and the challenges are separated into two main categories: (A) direct problems -including (1) medical staff, (2) hospital system and equipment, and (3) failure to work with patients and their peers and (B) indirect/environmental problems. India (19, 20, 59, 92, 94, 104) and Tanzania (43, 63, 79, 80, 105) have studied the barriers to in-hospital care more substantially. Figure 3 maps the prominent barriers to acute care of people with TSCI in these countries.

#### 3.3.1 Direct problems

##### 3.3.1.1 Medical staff

Including SCI treatment, resource utilization, post-discharge complications, medical staff capacity, patient education, surgical challenges, and barriers in attitudes of medical staff. Challenges in SCI treatment include inadequate nursing care (19), lack of knowledge, skills, and manpower to treat SCI among physicians (21, 26, 28, 29, 32, 33, 36, 41, 79, 93), increased workload per staff (22), and neglected SCI in traumatic patients (92). A lack of well-trained staff and awareness of TSCI complications can lead to premature discharge, deterioration, readmission, or even death (92). There are also deficient standard continuing medical education (CME) programs to keep doctors and medical staff up-to-date on novel management strategies in the TSCI (25). Ideally, doctors and other providers should educate the patients to understand and implement essential healing process steps which is a challenge in the developing world (25). Further exacerbating the problem, on-call neurosurgeons are often not available (106), and there is a disproportionately small number of trained spine surgeons compared to the regional population (28).

##### 3.3.1.2 Hospital system and equipment

Twenty-five included studies presented different problems that can be categorized as follows: lack of equipment and facilities, insufficient SCI centers, underdeveloped methods of care, delays and inappropriate referrals, post-acute management challenges, challenges in emergency care, treatment gap in surgery indications, lack of formal trauma care pathways, insufficient critical care facilities, limited multi-disciplinary care, and cost and expense challenges. Numerous studies mentioned a shortage of essential equipment and facilities crucial for SCI care (18, 22, 28, 36, 42, 63, 79, 82). These include specialized beds, catheters, invasive blood pressure monitoring, spinal braces, implants, and pedicle screws. Insufficient availability of SCI centers is identified as a problem in several studies (19, 28, 32, 36, 40). Delays for referral from regional hospitals to tertiary centers are reported in one study (80). Inappropriate prioritization of care and cases of intermediate admission were also noted (26, 79). Problems in emergency care included long stays at adult emergency centers without receiving adequate care and patients leaving without being admitted to intermediate admission (38, 41). Unfortunately, the protocols for trauma reception, patient transfer, triage, and management of patients are *ad hoc*, and there is no formal trauma care pathway, such as a detailed overall plan with specific stages, each with its unique characteristics (61, 107–109). Issues with ICUs included poor access (43, 93), inadequate availability (110), and a lack of 24/7 available operating rooms, surgical instruments, and imaging services (111).

##### 3.3.1.3 Failure to work with patients and their peers

Urinary tract infection (UTI), bedsores (21, 32), lack of familiarity of temporary personnel with cultural aspects to effectively communicate with the patients (26), and lack of awareness about SCI (92) were reported problems associated with ineffective patient communication.

#### 3.3.2 Indirect and environmental problems

Twelve included studies mentioned indirect and environmental problems including patient reluctance and economic constraints, insurance and emergency center challenges, the influence of local policies, population density and geographical differences, global inequity in resource distribution, and specific TSCI-related challenges. Patients’ reluctance to be discharged due to fear of loss of financial support is identified as an indirect problem (28). Economic and financial constraints posed challenges in SCI care, as illustrated in multiple studies (22, 36, 44, 80, 82, 92). Lack of insurance and overcrowded emergency centers were reported issues impacting SCI care (41, 80). Moreover, the lack of manpower and health facilities outside the capital city contributed to challenges in providing care (44). In addition to the aforementioned issues, there were other TSCI-specific problems, including a lack of educational brochures, unawareness of ASIA standards and SCI medicine by many physicians, and a lack of TSCI beds and rehabilitation units/specialists (28).

### 3.4 Post-hospital

**Figure 4:**
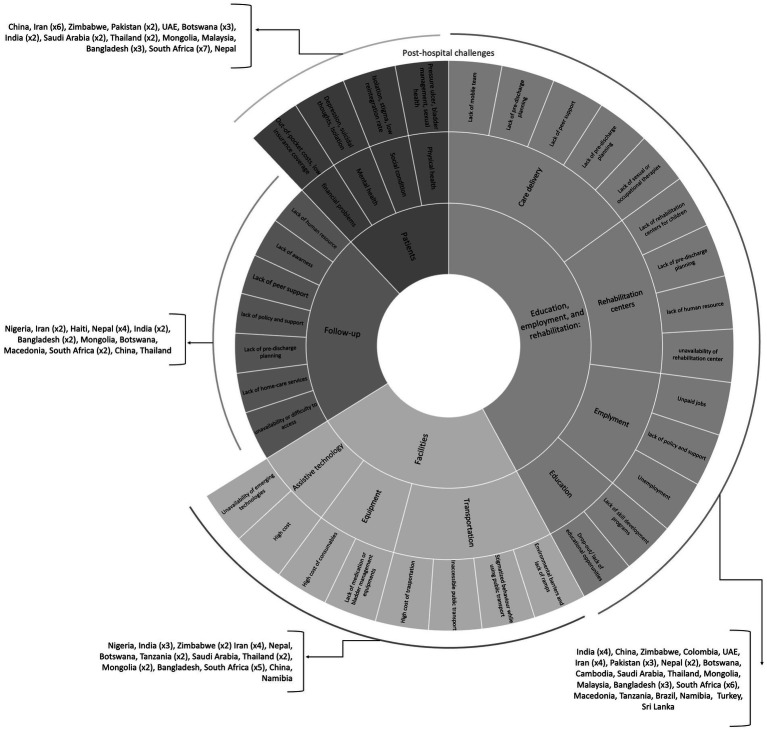
Post-hospital TSCI management challenges.

Most of the studies focused on the post-hospital challenges of patients with TSCI. Sixty-one articles mentioned insufficient facilities; patient education, rehabilitation, and employment; lack of systematic follow-up; and social, family, and personal limitations (Appendix B-Table S5). These studies highlighted the post-hospital care in 26 countries in which Iran (26, 30, 55, 58, 89), South Africa (65, 66, 72, 97, 101, 112), and Nepal (31, 34, 36, 53, 60) were most frequently studied. Figure 4 depicts a picture of the challenges that people with TSCI encounter after discharge.

#### 3.4.1 Facilities

Twenty-eight included studies pointed to diverse challenges in facilities categorized as follows: deficient medical and paramedical equipment, insufficient nursing and assistive services, ethical issues in allocating resources, inadequate building facilities, rescue and retrieval systems, environmental challenges, and challenges in mobility and independence. Lack of essential medical and paramedical equipment, such as catheters and wheelchairs, was identified as a significant barrier in numerous studies (18, 30, 38, 43, 74, 89, 91, 92, 100, 101). Issues related to the poor quality and high cost of purchasing or repairing paramedical equipment were delineated in specific studies (83, 99, 101). Inadequacy in nursing and assistance services was recognized as a barrier to SCI care (91, 99). Lack of facilities for social activities and ethical concerns in allocating facilities were noted challenges (26). Building facilities, including stair ramps and toilet modifications, were reported as inadequate for accessibility for individuals with SCI (30, 74). Inadequacies in rescue and retrieval systems posed challenges in certain settings (19, 32). Moreover, people with SCI face environmental challenges, including inadequate housing (23, 31) and accessibility to toilets, clean water, and electricity (31, 101). Environmental and structural barriers (81, 86, 88, 91, 101) along with unsupported public transportation (57, 88), are identified as significant obstacles. Challenges in mobility and independence lead individuals with SCI to abandon unsuitable assistive devices. These individuals also have trouble in independent access to the community due to their disability (77, 89) and challenging physical terrain (31, 35, 37).

#### 3.4.2 Education, employment, and rehabilitation

During post-hospital phases, the most cited problems were poor education in individuals with SCI, insufficient employment, and rehabilitation problems. Insufficient health literacy was reported in individuals with SCI and their families (67, 72). Poor education can cause an increased rate of avoidable complications including inappropriate bladder care (25), inadequate pressure ulcer care (90), lack of knowledge about pain management strategies (66), and misinformation about SCI rehabilitation (26, 89). Individuals also do not receive adequate information and training about coping with their problems, including maintaining their daily social and mental functions. In addition, educational resources like educational materials in the local language, sexual and reproductive health and consultation and education are scarce (26, 28, 72, 92, 98). Secondary to insufficient rehabilitation care centers, specialists, and teams, individuals faced challenges in the rehabilitation (21, 28, 36, 39, 40, 42, 44, 45, 51, 57, 73, 89, 92, 100, 101). Inadequacy or unavailability of pediatric SCI units (73), transportation problems (26), difficulties with discharge policy, unsuitable accommodations (73), lack of financial resources (23, 30, 37, 40, 45, 92), absence of rehabilitation guidelines, and inadequate availability of nursing homes contributed to rehabilitation challenges (38). Lack of a low-cost outpatient program (24, 89), negative societal attitude toward disability (39), lack of occupational therapy, and minimal social welfare assistance (23, 30, 55, 57, 92) were key factors contributing to insufficient employment of individuals with SCI. There is also a lack of systematic post-discharge plans for SCI rehabilitation, which can further exacerbate employment challenges.

#### 3.4.3 Follow-up

Nineteen studies reported follow-up challenges in SCI individuals that can be categorized into several items. Lack of SCI-related follow-up routines in the treatment of complications (18, 41, 51, 89) and unsuitable transport infrastructure for follow-up contribute to difficulties in travel (45, 92). Exorbitant healthcare policy (34, 57, 66, 74, 86, 89, 101) impacting access to follow-up care, inadequate governmental financial support (36), and financial constraints leading to fewer follow-ups also present significant financial barriers to comprehensive follow-up in these individuals (92). Non-negotiable actions typically dictated by healthcare providers (81) and limited peer support for SCI individuals and their care providers can make strict protocols difficult or even impossible to follow (44). Another important challenge is the discrimination in service delivery. In Low- and middle-income countries (LMICs) women mostly do not receive adequate physical, emotional, and social follow-up (96). Further treatments and systematic follow-up may be needed to improve the patient’s quality of life and survival (103). The lack of home visits after discharge and follow-up home care services were among the issues that caused poor outcomes for patients (28, 92).

#### 3.4.4 Patients

Forty-one studies identified a variety of challenges related to post-injury complications. Patients complain about Secondary Health Conditions (SHC), such as pressure ulcers, muscle spasms, bladder-related problems, and pain (38, 55, 70, 81, 83, 90, 95, 99, 101), lack of information regarding pressure relief, effective pain management (89, 90), clean intermittent catheterization and effective bladder and bowel management methods (38, 52, 69) among patients and caregivers. The sexual function of people with SCI is altered due to physical and psychological factors, a stressor that can be mitigated with proper sexual counseling; however, the patients often do not receive such services (98). The studies reported patients’ mental distress which includes struggling with experiences of denial, loss of dignity, low self-esteem, diminished motivation, feelings of discontentment and unimportance, isolation, depression, and even suicidal ideation (55, 66, 71, 78, 81, 88, 89, 96, 97, 101, 113). Patients with SCI dealt with social challenges like a low rate of reintegration into the community (21, 31, 92), stigma (30, 35, 37, 71, 78, 88, 91, 101), discrimination (55, 71), social isolation (55, 101), issues related to cultural belief, and dependency in activities (26, 81, 91). In addition, financial issues are one of the major complaints of people with SCI and their families (49, 55, 58, 60, 61, 89, 95, 96, 113). Issues in care delivery, such as the availability of prescribed and alternative medicine (86, 92, 101), and lack of home-based care (89, 90) add another layer of complexity. Lack of effective communication between healthcare providers and patients was also a barrier to obtaining healthcare services (72). Delayed initiation of rehabilitation may result in missing a critical window of opportunity before complications such as contractures become severely limiting (92). Although the needs of family members and informal caregivers are often neglected, they may also need special support. Sacrificing personal aspirations to care for their family member can influence their physical, mental, and social well-being (58, 59). On the other hand, informal caregivers do not have adequate, professional knowledge about supporting and providing care to the person with SCI (69, 70). Hiring a personal assistant would help people with SCI to perform their daily activities, self-care, and employment without dependence on their family members. However, the cost of these services is high and most families cannot afford it (88). The patients and their families point out that policymakers do not know their needs. Additionally, parts of written policies that may provide support or relief are not implemented (55).

Overall, the most mentioned challenges were related to patients’ post-injury complications and empowerment of people with SCI in post-hospital challenges, negligence and deficiencies in hospital systems and transportation in acute phase management, and infra-structural and legislation problems in injury prevention.

## 4 Discussion

In this scoping review, we holistically investigate the challenges in managing TSCI across developing countries into four broad chronologic phases including injury prevention, pre-hospital, in-hospital, and post-hospital phases. An important point to consider when interpreting the results of this review is that, although we assume all LMICs face similar obstacles in the management of TSCI, they have different challenges based on their region, Gross Domestic Product (GDP), and legislation.

### 4.1 Injury prevention

In this review, we identified several challenges in injury prevention. These include insufficient valid data from well-defined research, gaps in preventive legislation, cultural barriers to implementing preventive strategies, and a lack of well-defined infrastructure to address injury prevention.

Consistent with other studies, our findings emphasized the insufficiency of research on the cause and epidemiology of trauma in developing countries (114, 115). Addressing these challenges is crucial in the injury prevention phase of SCI management and properly conducted epidemiological studies play a pivotal role, as they have the potential to bring about change by presenting issues and proposing clear preventive measures, influencing regional policy (92, 114, 116). Given the regional nuances in the effectiveness of preventive measures, there is a pressing need for the collection of sufficient, specific data (92). This necessitates the establishment and funding of research centers (117), the implementation of technology, and the creation of research opportunities (118). Failure to prioritize these efforts may result in a stalling of developments in SCI prevention indefinitely. Notably, there is a current dearth of reports for many developing countries, which may be grappling with these challenges without the necessary resources or attention to properly address them. Enhancing an interaction between the researcher and health-care system and evidence-based policy-making should be also prioritized to enhance more evidence-based policy-making (116).

Motor Vehicle Crash (MVC) is the leading cause of TSCI in developing countries (119). Other studies recognized high-speed driving, alcohol consumption before driving, nonexistent alcohol breath testing, lack of using seatbelts, helmets or child restraints, neglecting vulnerable road user such as pedestrians, cyclists and motorcyclists, and non-standardized roads and vehicles as the challenges in injury prevention (116, 120, 121) which were in concordance with our results. Excess speed is not only associated with a higher risk of injury, but also the severity of injury and is known as a priority in preventing MVC (122). Although public educative interventions can be effective, but legislative enforcement is the most cost-effective approach to controlling speed and alcohol consumption supported with a high level of evidence (123) whereas the legislative enforcement is inadequate in LMICs (124). The health sector’s limited participation in prevention efforts, coupled with the inappropriate allocation of resources (117, 118) further exacerbates the management limitations in these regions. It is crucial to recognize that effective prevention strategies require tailored legislative frameworks for each country. These frameworks should be developed based on the country’s available resources, facilities, and cultural nuances. Striking the right balance ensures that legislation is not only acceptable but also applicable, fostering a comprehensive approach to SCI prevention in diverse socio-cultural contexts.

Additionally, lack of standardized roads and vehicles posed infrastructural obstacles in the prevention of MCVs (125). In developing countries, the application of effective engineering methods for vehicles and roads has been inconsistent (117). These infrastructural challenges can be systematically categorized into five domains critical for injury prevention: policy, involving inappropriate construction of settlements, roads, and pedestrian areas (126); culture (127); investment, characterized by inadequate resources for law enforcement and injury prevention; education, with a lack of systematic education for personnel, particularly in work-related settings; and laws, reflecting an inadequate safety culture, poor legal conditions, and insufficient regulations for motor vehicles (119). Motor vehicle maintenance including periodic inspection of vehicles specially busses and old vehicles has been effective in preventing MVC (117, 121). Sweden implemented a road safety policy named “Vision Zero” which prioritizes the protection of vulnerable road users. In this program the responsibility for road safety lies primarily with the system designers and it focuses on environmental changes over human-related factors (123).

Although the government plays a vital role in TSCI management, without the active participation of the people at risk for trauma, the designed strategy will fail. Relying solely on legal enforcement of seat belts and motorcycle helmets does not guarantee their consistent application in the real world (128). The use of seatbelts in developed (i.e., United States) and developing (i.e., Saudi Arabia) countries is about 85 and 5%, respectively (103). The result of a trial on the effect of four preventive interventions, including (1) helmet laws for motorcycles, (2) seatbelt laws for cars, (3) implementation of general traffic laws, and (4) national media educational activities on community behavior indicated that the ratio of subjects using a seatbelt or helmet did not improve (129). Montazeri et al. suggest that knowledge and awareness about high-risk behavior do not always affect the behavior (128).

Work-related injuries are contributed to 15% of TSCI (13). To mitigate these injuries, risk analysis of the workplace and improved safety measures are necessary (36, 51). Interventions targeting improvements in policy, standardization, and laws were the most effective preventive practices in developed countries (125, 130).

### 4.2 Pre-hospital

The included studies reported the absence of safe and timely prehospital transportation, inadequate public education, lack of a well-defined pre-hospital trauma system and referral network for managing TSCI, insufficient equipment, and disparities in implementing trauma guidelines.

A major component of the trauma system is pre-hospital care which refer to identifying, managing TSCI cases and transporting them safely to the hospital (8). An effective trauma system needs coordination between different sectors including governmental agencies, legislative sectors, and private and community-based institutions. The absence of a structured and integrated referral system are revealed by the findings of several studies which is consistent with prior studies (131). In addressing the challenges within the pre-hospital system and communication network, a comprehensive strategy is essential to establish well-coordinated and efficient neurotrauma care systems in developing countries. This involves refining pre-hospital systems, fortifying communication networks, and ensuring clarity in the roles of operational teams in developing countries (132). Bridging the gap between urban and rural areas in terms of personnel availability is imperative to establish an equitable and effective neurotrauma care framework. Additionally, streamlining the patient journey to referral centers is crucial to optimizing outcomes and preventing unnecessary delays in critical interventions.

Many developing countries lack a well-defined pre-hospital system and communication network to manage TSCI patients (133). This shortcoming leads to unsatisfactory emergency medical services (EMS) interventions (134) and thus excessive mortality. The studies indicated ambiguity in the respective roles of operational teams like EMS, fire station, and police to reach the scene and manage emergent situations (107). This issue can be exacerbated by inequality in the availability of reserve personnel between urban and rural areas (133). Unfortunately, patients often reach referral centers after being transferred between multiple institutions—a major concern for patients who require emergent intervention. This practice may result in the loss of the crucial golden time for decompression surgery, leading to more serious complications.

Safe and prompt transportation is a significant factor in reducing mortality and severity of injury following TSCI. The considered challenges were (1) transportation by untrained personnel (18, 33, 75, 92) or lay responders who are unaware of log rolling and immobilization techniques, (2) delay in transportation to SCI centers and (3) lack of equipment. WHO has identified knowledge and skills, and equipment as essential factors in providing an efficient trauma care (135).

Consistent with other studies, A lack of skilled personnel and paramedics is a challenge in transportation and delivering acute pre-hospital care to individuals with TSCI which results in increased death or injury severity (11, 131). A nationwide study in Japan showed that physician-led pre-hospital management reduced in-hospital mortality regardless of transportation time (124, 136). Rathore et al. found insufficient knowledge among healthcare providers in Pakistan regarding immobilization techniques and log rolling in patients with suspected SCI (28). These results underscore the need for nationally relevant guidelines or adaptation of international ones for managing TSCI in developing countries.

In developing countries, lay responders have a significant impact as the front line of informal trauma care (11, 131). Achieving adequate public education such that responders to trauma patients use resuscitation and immobilization strategies to prevent the worsening of neurological deficits in patients with incomplete SCI is a significant challenge (92). Several reports indicated that untrained people helping the injured can lead to or exacerbate an underlying TSCI (18, 19, 21, 25, 28, 29, 32, 33, 36, 41, 48, 92, 93). Such injury-worsening events might also occur frequently in post-disaster situations, such as earthquakes, due to the need to remove people from the rubble. In a study conducted in Saudi Arabia, one-third of respondents did not know the signs of cervical SCI and two-thirds of them were not aware of first-aid responses to cervical SCI (56). Similarly, a study from Pakistan that evaluated the management of patients following the 2005 earthquake reported that none of the 83 injured people received immobilization at the scene (28). Promoting first aid knowledge and communicating the trauma and immobilization protocols through social media platforms and mobile apps are encouraged for enhancing public education (4).

Delay in transportation is another impediment in the pre-hospital management of TSCI. Prehospital time consists of activation time, response time, scene time and transport time which is varied across the countries (131). Significant disparities exist in the average time from injury to hospital admission, ranging from 2.8 to 5.9 days in Tanzania, 45 days in India, and 17 days in Sierra Leone (79). Very few TSCI patients reached care in less than 24 h (23, 117, 126, 128). This is likely due to inappropriate triage criteria (117) and inter-hospital transfer issues (137) that may be compounded by poor roads and difficult geography. In a study conducted in Tanzania, a longer time between injury and admission was associated with lower odds of mortality which is in contrast with the guidelines that early admission and treatment can reduce complications and improve patient-related outcomes (63). In some developing countries, the insurance race can determine the hospital to which patients are referred (138).

Equipment deficiencies can be divided into three categories: (1) lack of adequate vehicles for transportation, (2) lack of technology devices in ambulances such as ventilators, blood pressure monitors, and pulse oximeters, (3) lack of standard equipment in ambulances such as standard long backboards for vertebral fracture patients, cervical collars, sandbags, and warmers. There are numerous reports that in many developing countries, untrained individuals with unsuitable vehicles, including motorcycles, busses, bicycles, and private cars transport injured patients to hospitals (23, 52, 57, 119, 126, 133, 139, 140). In some developing countries like Iran (128) and Pakistan (139), air transportation is available, particularly from rural regions. This is beneficial to provide life-saving care (134).

### 4.3 In-hospital

In-hospital challenges included a lack of well-trained staff, insufficient Intensive Care Units (ICUs) and other facilities, lack of tertiary centers and non-standard emergency management to improve TSCI care, and ineffective patient-doctor communication. Patients in developing countries have a lower disability survival rate than those in high-income countries (4). This trend can be also seen also for TSCI patients and may be due to LMICs’ differences in medical care and sociocultural factors. For example, a lack of formal trauma care pathway results in support only in the initial management or in the end-life support for critically ill patients before or after admission, resulting in a more favorable prognosis as the patient arrives at the hospital (119, 130, 133).

Formative resource utilization and care should aim to reduce re-admission and post-discharge complications. Insufficient trained human resources and high workloads pose challenges in delivering an optimal neurotrauma care. A lack of well-trained staff and awareness of TSCI complications can lead to premature discharge, deterioration, readmission, or even death (92). Limited availability of neurosurgeons and small number of trained spine surgeons compared to the regional population are often not available (106), and there is a disproportionately small number of trained spine surgeons compared to the regional population (28). The ratio of neurosurgeons to population is meager in many developing countries: 1:7,000,000 in sub-Saharan Africa, 1:9,000,000 in East Africa, and 1:12,000,000 in Uganda and Tanzania (141). Establishing a neurotrauma fellowship to educate sub-specialized neurosurgeons have been suggested as a strategy of improving the level of care (142). Additionally, many patients and their families reported inappropriate attitudes from health workers as a barrier to proper care delivery.

Some studies pointed to underdeveloped methods of care for SCI patients within general hospitals and level one trauma centers were associated with mortality (143). Patients’ impaired hemodynamics such as bradycardia and hypotension are associated with higher mortality rate following TSCI (144). In developing countries, patients do not receive Mean Arterial Pressure (MAP)-targeted therapy in general hospitals mostly due to the unavailability of ICU and monitoring facilities (145). Thus, a certain level of development is generally needed before tertiary care like neurosurgery is a viable goal. Most developing countries are still lacking critical TSCI care supplies, such as intermittent pneumatic compression stockings and specialized mattresses (146). On the other hand, delays for referral from regional hospitals to tertiary centers are reported in our findings, as well as other studies (147). Hospital crowding, influenced by policies, can reduce the quality of care by increasing wait times (148). Population density and geographical differences were other influential factors. When compared to rural hospitals, urban hospitals are better equipped and have highly trained providers (Saudi Arabia, India, Zimbabwe, and Iran) (23, 149–151). Global inequity in resource distribution was another problem (92). This unequal distribution was evident in the Lancet Global Surgery 2030 report that highlights discrepancies in accessing basic surgical needs (152).

Studies have shown decompressive surgery within 24 h after the injury is associated with higher neurological recovery (153). However, this timeline seems unrealistic in developing countries. Structural barriers like inadequate Operating Rooms (OR), surgical equipment and lack of trained staff are significant factors causing delay in surgical management of people with TSCI. A study conducted in Tanzania reported only 25% of patients with surgery indications were operated on. Unfortunately, the protocols for trauma reception, patient transfer, triage, and management of patients are *ad hoc*, and there is no formal trauma care pathway, such as a detailed overall plan with specific stages, each with its unique characteristics (61, 107–109).

Many studies identified patient reluctance and economic constraints as a significant barrier in providing emergency care and a barrier in taking surgical management. It is noteworthy that the insurance status of patients also influences the transfer process, further contributing to patient anxiety surrounding their financial stability after SCI (104).

Post-acute management during hospital admission is an important factor in reducing the complications of TSCI. Creating an effective physician-patient communication have a positive impact on accepting the new conditions and educating patients and their family about necessary considerations (125). Having a “turning team” to change the position of patients every two hours and persuading the patients to sleep on their faces can help prevent decubitus ulcers and reduce their treatment costs.

### 4.4 Post hospital

Individuals with SCI are posed to a myriad of interconnected challenges in dealing with this chronic condition. Our study highlights secondary health conditions, inadequate rehabilitation and patient education; insufficient facilities; lack of systematic follow-up; and social and occupational limitations as common post-hospital issues.

People with SCI must cope with and manage complications related to this chronic condition including physical secondary health conditions, psychological distress and sexual dysfunction. Other studies have demonstrated Secondary Health Conditions (SHC) such as pressure ulcer and UTI are the primary causes of more severe long-term disability and premature death among people with SCI (154). Effective management of urine and bowel incontinence, pain and pressure ulcer prevention play a crucial role in enhancing their quality of life (35). A significant challenge in the management of SHC and sexual dysfunction lies in the insufficient education provided to patients and their families regarding bowel and urinary management as well as the lack of counseling opportunities for sexual adjustment. Enhancing self-efficacy and self-management in preventing SHC is associated with higher adjustment and quality of life among people with SCI which can be obtained by empowering and educating the patients about their new conditions (125, 155). Additionally, there remains limited awareness about pain management, clean intermittent catheterization and urodynamic evaluation methods for neurogenic bladder (92). Psychological distress, such as depression and anxiety, is prevalent among individuals with SCI. It is recommended to screen for mental disorders during the early stages after the injury and discharge (125). People who maintain good mental well-being tend to adapt more easily to their new condition (125). Empowering and educating patients and their caregivers in coping with disability and complications caused by SCI should be more emphasized.

The present study highlights considerable challenges related to rehabilitation and follow-up for individuals with SCI. Appropriate rehabilitation to adapt to the individual’s new situation is critical and can substantially influence outcomes. The patterns of life, communication, and expectations are often set favorably in rehabilitation through interacting with other SCI peers. The World Health Organization (WHO) recognizes rehabilitation as an essential health service and a core component of universal health coverage (14). However, barriers hinder timely access to rehabilitation services (124). For instance, in Nepal a wide gap between hospital discharge and rehabilitation initiation led to high rates of pressure ulcers affecting 32% of admitted individuals (156). To address these challenges, stakeholder groups should prioritize rehabilitation services (14) and training multi-disciplinary and specialized rehabilitation teams is essential (157, 158). Alternative services, such as home-based rehabilitation and community-based rehabilitation have shown promising results (28, 159, 160). Social Follow-up systems are also inefficient in developing countries due to various causes such as the lack of a surveillance system to collect patients’ records and information, structural and cultural barriers in determining a follow-up plan at the time of discharge, or financial constraints. Additionally, many rehabilitation centers do not provide home-care services. Eslami et al. argued that the high rate of pressure ulcers in SCI patients in developing countries like Iran is due to the fee-for-service model but there is no responsible party for those not referred or who cannot pay (161). The lack of home visits after discharge and follow-up home care services were among the issues that caused poor outcomes for patients (28, 92). Mobile teams could be a method to increase effective follow-up care (26). One of the best practices in follow-up suggested by Liu et al. was to follow the patients with regular phone calls and home visits, which could result in better management of secondary health conditions and improved assessment of patients’ home environment, facilitating the alliance of patient and health providers in decision-making (81). Follow-ups provide a support system in managing SHC and other SCI complications and it should constantly assess the Quality of Care (QoC) for people with SCI. A comprehensive assessment tool developed by Ghodsi et al. evaluates the QoC in three categories of indicators for TSCI management including pre-hospital, in-hospital, and post-hospital management of TSCI (140) and it provides a valuable resource for evaluating and improving TSCI care (162). Without essential rehabilitation, the injured person’s expectations might be unduly stilted. Inpatient and outpatient rehabilitation are critical to educate the injured person on how to avoid complications and help them cope with the injury state and achieve a higher quality of life.

Social reintegration is another predictor of quality of life in individuals with SCI. An important factor in improving social participation is developing an enabling environment by enhancing access to transportation, and buildings (163). The absence of policy framework for accessibility standards and insufficient financial resources allocated to addressing these policies, and lack of public awareness about the importance of accessibility have been noted as main challenges in providing an enabling environment (125). A limited number of studies conducted in developing countries mentioned adjusting these modifications to their housing, while it enhances safety, independence, and quality of life of individuals with SCI (164). This limited number of home modifications in developing countries can be due to financial barriers in affording such environmental modifications. The family might not know how to modify their homes to improve the injured person’s independence.

Employment varied according to the type and severity of injury. Only 12% of those who were employed at the time of injury were able to return to their jobs or similar jobs after injury due to factors such as a lack of proper occupational rehabilitation programs, insurance failing to cover rehabilitation costs (165), and changes in psychological issues and self-esteem (166, 167). SHCs are a barrier in employment of people following TSCI (168). Most people with SCI cannot continue their prior careers and informal caregivers have to abandon their job due to the high burden of caring for disabled family members. The out-of-pocket payments of healthcare services (e.g., rehabilitation), and lack of insurance coverage put vulnerable groups at the risk of poverty and other socio-economic consequences (169). It is recommended to investigate the financial implications of TSCI and compensative interventions specifically in the context of LMICs in future studies.

Consistent with other studies, we found a limited access to emerging technologies in developing countries (170). Although these technologies had some advantages in enabling individuals with TSCI, the used technology-intensive equipment that was difficult to repair and maintain. The costs of this equipment were not all covered by insurance, which might adversely affect psychosocial outcomes (166). There are ethical dilemmas in allocating the available resources (96). Ethical challenges in the management of TSCI are not limited to assistive technologies. An important ethical challenge is the patients’ autonomy during their treatment or rehabilitation process (96). People with SCI must be engaged not only in their treatment process, but also in policy-making and their experience can provide invaluable insights (125).

### 4.5 Limitations

We would like to acknowledge certain limitations in our study including: **I. Variations in Definitions and Diagnostic Criteria**: A notable limitation is the heterogeneity in the definitions and diagnostic criteria for TSCI across the included studies. This variability may affect the comparability of results and the generalizability of findings across different settings and populations. The diversity in methodologies and definitions could lead to discrepancies in interpreting the magnitude and specifics of challenges in TSCI care. **II. Geographical Limitations in Data**: The study’s findings are predominantly based on data from a limited number of developing countries, which may not fully represent the global spectrum of challenges in TSCI care. There is a lack of comprehensive data covering all developing countries, leading to potential bias in understanding the global situation. The absence of information from numerous regions might limit the applicability of the recommendations and findings to those areas not covered in the review. **III. Focus on Specific Phases of Care**: There is an observed imbalance in the quantity of research focusing on different phases of TSCI care, with a notably lesser focus on injury prevention. This imbalance highlights a potential gap in the literature and underscores the need for further research in underrepresented areas, particularly in developing effective injury prevention strategies and interventions. **IV. Unpublished Data and Gray Literature**: The scoping review methodology did not include unpublished data or gray literature, which could contain relevant information on the challenges of TSCI care in developing countries. This exclusion might overlook valuable insights, innovative practices, or local solutions that have not been formally published, potentially biasing the findings toward published literature. **V. Potential for Publication Bias**: Given the reliance on published studies, there is a risk of publication bias, where studies reporting significant or positive findings are more likely to be published than those with negative or inconclusive results. This bias could skew the understanding of challenges in TSCI care, emphasizing certain issues over others that might be equally critical but less reported. **VI. Cultural and Socio-economic Contexts**: The review acknowledges challenges in TSCI care but may not fully encapsulate the depth and impact of cultural and socio-economic contexts on these challenges. The socio-cultural factors that influence the implementation of care strategies, patient and caregiver behaviors, and the adoption of prevention measures are complex and multifaceted, requiring further exploration beyond the scope of this review.

## 5 Conclusion

The goals of TSCI care are to increase survival, prevent further injury, reduce complications, and set the path to living safely and productively with SCI. To establish a conceptual framework for TSCI care in developing countries, we categorize and summarize the current challenges from different developing countries into four significant areas based on the timeline from injury, including injury prevention, pre-hospital care, hospital care, and post-hospital care. Most studies focused on in-hospital and post-hospital care, which fall into secondary and tertiary prevention strategies. We strongly recommend that researchers and policy-makers appreciably highlight the primary prevention strategies that may prohibit the trauma from happening. International guidelines can provide benchmarks, but overcoming these challenges is a complex problem. Potential solutions should be approached with consideration for underlying regional and cultural factors, interconnecting networks, and the interest of different stakeholders to produce stepwise progress. Often, better outcomes can be achieved with existing capabilities through systematic education and rewarding compliance to care standards, aided internationally where appropriate. Leveraging widely available technology such as cell phones through creative approaches, including telemedicine, education, compliance monitoring, emergency response systems, and data collection, can be instrumental in addressing challenges and improving outcomes in TSCI care in developing countries. The challenges presented here can serve as goals for policymakers and healthcare providers as focus areas for progress.

## Data availability statement

The original contributions presented in the study are included in the article/Supplementary material, further inquiries can be directed to the corresponding author.

## Ethics statement

The Ethics Committee of Tehran University of Medical Sciences approved the study with the reference number 97-02-38-347.

## Author contributions

MR: Conceptualization, Data curation, Formal analysis, Investigation, Methodology, Project administration, Validation, Visualization, Writing – review & editing, Resources, Software, Writing – original draft. EK: Data curation, Formal analysis, Investigation, Methodology, Resources, Software, Validation, Visualization, Writing – original draft, Writing – review & editing. MH: Data curation, Formal analysis, Investigation, Methodology, Resources, Validation, Visualization, Writing – original draft, Writing – review & editing. FF: Data curation, Investigation, Methodology, Resources, Validation, Visualization, Writing – original draft, Writing – review & editing, Conceptualization, Software, Supervision. ZG: Conceptualization, Data curation, Investigation, Methodology, Resources, Validation, Visualization, Writing – review & editing, Project administration, Supervision, Writing – original draft. SR: Data curation, Investigation, Resources, Writing – original draft, Writing – review & editing, Visualization. SG: Data curation, Investigation, Visualization, Writing – original draft, Writing – review & editing, Resources. MA: Data curation, Investigation, Visualization, Writing – original draft, Writing – review & editing, Resources. AA: Data curation, Investigation, Visualization, Writing – original draft, Writing – review & editing, Resources. SH: Writing – original draft, Writing – review & editing, Resources, Data curation, Investigation, Methodology, Visualization. MS-N: Investigation, Methodology, Visualization, Writing – review & editing, Conceptualization, Resources. RA: Data curation, Investigation, Methodology, Writing – review & editing, Resources. SK: Data curation, Investigation, Resources, Writing – review & editing, Visualization. AV: Writing – review & editing, Validation. JG: Validation, Writing – review & editing, Visualization. MF: Validation, Writing – review & editing. VR-M: Validation, Writing – original draft, Writing – review & editing, Conceptualization, Data curation, Formal analysis, Investigation, Methodology, Project administration, Supervision, Visualization, Resources.

## References

[1] Safdarian M Trinka E Rahimi-Movaghar V Thomschewski A Aali A Abady GG . Global, regional, and national burden of spinal cord injury, 1990–2019: a systematic analysis for the global burden of disease study 2019. Lancet Neurol. (2023) 22:1026–47. doi: 10.1016/S1474-4422(23)00287-9, PMID: 37863591 PMC10584692

[2] Golestani A Shobeiri P Sadeghi-Naini M Jazayeri SB Maroufi SF Ghodsi Z . Epidemiology of traumatic spinal cord injury in developing countries from 2009 to 2020: a systematic review and meta-analysis. Neuroepidemiology. (2022) 56:219–39. doi: 10.1159/000524867 35512643

[3] Quake SYL Khoda F Arjomandi Rad A Subbiah Ponniah H Vardanyan R Frisoni P . The current status and challenges of prehospital trauma care in low-and middle-income countries: A systematic review. Prehosp Emerg Care. (2024) 28:76–86. doi: 10.1080/10903127.2023.2165744, PMID: 36629481

[4] Shanthakumar D Payne A Leitch T Alfa-Wali M. Trauma care in low-and middle-income countries. Surgery J. (2021) 7:e281–5. doi: 10.1055/s-0041-1732351 PMC8536645 34703885

[5] Ona A Strøm V Lee B-S Le Fort M Middleton J Gutenbrunner C . Health inequalities and income for people with spinal cord injury. A comparison between and within countries. SSM Popul Health. (2021) 15:100854. doi: 10.1016/j.ssmph.2021.100854, PMID: 34258374 PMC8259327

[6] Malekzadeh H Golpayegani M Ghodsi Z Sadeghi-Naini M Asgardoon M Baigi V . Direct cost of illness for spinal cord injury: a systematic review. Glob Spine J. (2022) 12:1267–81. doi: 10.1177/21925682211031190, PMID: 34289308 PMC9210246

[7] Mock C Joshipura M Arreola-Risa C Quansah R. An estimate of the number of lives that could be saved through improvements in trauma care globally. World J Surg. (2012) 36:959–63. doi: 10.1007/s00268-012-1459-6, PMID: 22419411

[8] Callese TE Richards CT Shaw P Schuetz SJ Paladino L Issa N . Trauma system development in low-and middle-income countries: a review. J Surg Res. (2015) 193:300–7. doi: 10.1016/j.jss.2014.09.040 25450600

[9] Hyder AA Botchey I Mehmood A Kobusingye O Razzak J. Developing and evaluating trauma care systems in low- and middle-income countries (LMICs): experiences in africa. Injury Prevention. (2016) 22:A12–A13.

[10] Rasouli MR Nouri M Zarei M-R Saadat S Rahimi-Movaghar V. Comparison of road traffic fatalities and injuries in Iran with other countries. Chinese J Traumatol. (2008) 11:131–4. doi: 10.1016/S1008-1275(08)60028-0, PMID: 18507940

[11] Eisner ZJ Delaney PG Widder P Aleem IS Tate DG Raghavendran K . Prehospital care for traumatic spinal cord injury by first responders in 8 sub-Saharan African countries and 6 other low-and middle-income countries: a scoping review. Afr J Emerg Med. (2021) 11:339–46. doi: 10.1016/j.afjem.2021.04.006, PMID: 34141529 PMC8187159

[12] Zakrasek E Creasey G Crew J. Pressure ulcers in people with spinal cord injury in developing nations. Spinal Cord. (2015) 53:7–13. doi: 10.1038/sc.2014.179, PMID: 25366536

[13] Rubiano AM Carney N Chesnut R Puyana JC. Global neurotrauma research challenges and opportunities. Nature. (2015) 527:S193–7. doi: 10.1038/nature16035, PMID: 26580327

[14] Neill R Shawar YR Ashraf L Das P Champagne SN Kautsar H . Prioritizing rehabilitation in low-and middle-income country national health systems: a qualitative thematic synthesis and development of a policy framework. Int J Equity Health. (2023) 22:91. doi: 10.1186/s12939-023-01896-5, PMID: 37198596 PMC10189207

[15] Tricco AC Lillie E Zarin W O'Brien KK Colquhoun H Levac D . PRISMA extension for scoping reviews (PRISMA-ScR): checklist and explanation. Ann Intern Med. (2018) 169:467–73. doi: 10.7326/M18-0850, PMID: 30178033

[16] Munn Z Stern C Aromataris E Lockwood C Jordan Z. What kind of systematic review should I conduct? A proposed typology and guidance for systematic reviewers in the medical and health sciences. BMC Med Res Methodol. (2018) 18:1–9. doi: 10.1186/s12874-017-0468-4 29316881 PMC5761190

[17] International Monetary Fund. Group and aggreagtes information. April 2023. Available at: https://www.imf.org/en/Publications/WEO/weo-database/2023/April/groups-and-aggregates.

[18] Iwegbu C. Traumatic paraplegia in Zaria, Nigeria: the case for a centre for injuries of the spine. Spinal Cord. (1983) 21:81–5. doi: 10.1038/sc.1983.11, PMID: 6866559

[19] Chacko V Joseph B Mohanty S Jacob T. Management of spinal cord injury in a general hospital in rural India. Spinal Cord. (1986) 24:330–5. doi: 10.1038/sc.1986.48 3774371

[20] Shanmugasundaram T. The care of SCI patients in the developing nations—can we stem the rot? Spinal Cord. (1988) 26:10–1. doi: 10.1038/sc.1988.4, PMID: 3353120

[21] Wang D Wu X Shi G Wang Y. China's first total care unit for the spinal cord injured. Spinal Cord. (1990) 28:318–20. doi: 10.1038/sc.1990.41, PMID: 2235040

[22] Faure J. Spinal cord injury management in 1990. South Afr Med J. (1990) 78:443–4. PMID: 2218776

[23] Levy L Makarawo S Madzivire D Bhebhe E Verbeek N Parry O. Problems, struggles and some success with spinal cord injury in Zimbabwe. Spinal Cord. (1998) 36:213–8. doi: 10.1038/sj.sc.3100574, PMID: 9554024

[24] Lugo LH Salinas F García HI. Out-patient rehabilitation programme for spinal cord injured patients: evaluation of the results on motor FIM score. Disabil Rehabil. (2007) 29:873–81. doi: 10.1080/09638280701455494, PMID: 17577722

[25] Pandey V Nigam V Goyal T Chhabra H. Care of post-traumatic spinal cord injury patients in India: an analysis. Indian J Orthop. (2007) 41:295–9. doi: 10.4103/0019-5413.36990, PMID: 21139781 PMC2989513

[26] Raissi GR. Earthquakes and rehabilitation needs: experiences from Bam, Iran. J Spinal Cord Med. (2007) 30:369–72. doi: 10.1080/10790268.2007.11753954 17853660 PMC2031928

[27] Nwadinigwe C Ugezu A. Management of penetrating spinal cord injuries in a non spinal centre: experience at Enugu, Nigeria. Nigerian J Med. (2008) 17:205–9. doi: 10.4314/njm.v17i2.37385 18686841

[28] Rathore FA Farooq F Muzammil S New PW Ahmad N Haig AJ. Spinal cord injury management and rehabilitation: highlights and shortcomings from the 2005 earthquake in Pakistan. Arch Phys Med Rehabil. (2008) 89:579–85. doi: 10.1016/j.apmr.2007.09.027, PMID: 18295642

[29] RC BHA. Development of a rehabilitation programme after the earthquake in Haiti: opportunities and challenges from emergency to post-acute care. Physiotherapy. (2011) 97

[30] Babamohamadi H Negarandeh R Dehghan-Nayeri N. Barriers to and facilitators of coping with spinal cord injury for Iranian patients: a qualitative study. Nurs Health Sci. (2011) 13:207–15. doi: 10.1111/j.1442-2018.2011.00602.x, PMID: 21595815

[31] Scovil CY Ranabhat MK Craighead IB Wee J. Follow-up study of spinal cord injured patients after discharge from inpatient rehabilitation in Nepal in 2007. Spinal Cord. (2012) 50:232–7. doi: 10.1038/sc.2011.119, PMID: 22025245

[32] Singh R. Epidemiology of spinal cord injuries: Indian perspectives. Epidemiol Spinal Cord Injuries. (2012):157–68.

[33] Razzak ATMA. Early care following traumatic spinal cord injury (TSCI) in a rehabilitation Centre in Bangladesh-an analysis. Disabil CBR Inclusive Dev. (2013) 24:64–78. doi: 10.5463/dcid.v24i2.211

[34] Shah N Shrestha B Subba K. Spinal cord injury rehabilitation in Nepal. JNMA J Nepal Med Assoc. (2013) 52:427–31. doi: 10.31729/jnma.1531, PMID: 24362674

[35] Oderud T. Surviving spinal cord injury in low income countries. Afr J Disabil. (2014) 3:1–9. doi: 10.4102/ajod.v3i2.80 PMC5442511 28730012

[36] Shrestha D. Traumatic spinal cord injury in Nepal. Kathmandu Univ Med J. (2014) 12:161–2. doi: 10.3126/kumj.v12i3.13707 PMID: 25855104

[37] Löfvenmark I Norrbrink C Nilsson Wikmar L Löfgren M. ‘The moment I leave my home–there will be massive challenges’: experiences of living with a spinal cord injury in Botswana. Disabil Rehabil. (2016) 38:1483–92. doi: 10.3109/09638288.2015.1106596, PMID: 26694314

[38] Löfvenmark I Hasselberg M Nilsson Wikmar L Hultling C Norrbrink C. Outcomes after acute traumatic spinal cord injury in Botswana: from admission to discharge. Spinal Cord. (2017) 55:208–12. doi: 10.1038/sc.2016.122 27527239

[39] Rathore FA. Revisiting the 2005 earthquake paraplegics: what has changed in a decade? J Ayub Med Coll Abbottabad. (2015) 27:513–4. 26720995

[40] Al-Chalabi KM. Spinal cord injuries in UAE: retrospective, demographic & overview study of patients admitted & managed in neuro-spinal hospital Dubai during last 12 years. Int J Phys Med Rehabil. (2015) 3

[41] Debebe F Woldetsadik A Laytin AD Azazh A Maskalyk J. The clinical profile and acute care of patients with traumatic spinal cord injury at a tertiary care emergency Centre in Addis Ababa, Ethiopia. Afr J Emerg Med. (2016) 6:180–4. doi: 10.1016/j.afjem.2016.06.001, PMID: 30456092 PMC6234159

[42] Choi J-H Park PJ Din V Sam N Iv V Park KB. Epidemiology and clinical management of traumatic spine injuries at a major government hospital in Cambodia. Asian Spine J. (2017) 11:908–16. doi: 10.4184/asj.2017.11.6.908, PMID: 29279746 PMC5738312

[43] Moshi H Sundelin G Sahlen K-G Sörlin A. Traumatic spinal cord injury in the north-East Tanzania–describing incidence, etiology and clinical outcomes retrospectively. Glob Health Action. (2017) 10:1355604. doi: 10.1080/16549716.2017.1355604 28856978 PMC5645664

[44] Munakomi S Bhattarai B Cherian I. Prospective observational research on the clinical profile and outcome analysis among a cohort of patients sustaining traumatic cervical spine and cord injury in a peripheral tertiary spine care Centre in Nepal. F1000Res. (2017) 6:6. doi: 10.12688/f1000research.12911.1 PMC5701450 29250317

[45] Suman D. WhatsApp as a tool to improve distance urology care and follow-up of spinal cord injury (sci) patients in developing countries. Neurourology and urodynamics. Hoboken, NJ: Wiley (2017).

[46] Alwashmi AH. Vocational rehabilitation awareness among spinal cord injury male patients in Saudi Arabia: a brief communication. Cureus. (2019) 11:e3886. doi: 10.7759/cureus.3886 30911443 PMC6424544

[47] Dorjbal D Prodinger B Zanini C Avirmed B Stucki G Rubinelli S. Living with spinal cord injury in Mongolia: a qualitative study on perceived environmental barriers. J Spinal Cord Med. (2020) 43:518–31. doi: 10.1080/10790268.2019.1565707 30633693 PMC7480610

[48] Ghajarzadeh M Saberi H. Transportation mode and timing of spinal cord decompression and stabilization in patients with traumatic spinal cord injury in Iran. Spinal Cord. (2019) 57:150–5. doi: 10.1038/s41393-018-0189-5, PMID: 30201998

[49] Rahman PA Abdul Hakim MU Che Daud AZ Ahmad Sharoni SK. Sexuality among men with spinal cord injury. Indian J Public Health Res Dev. (2019) 10:1286. doi: 10.5958/0976-5506.2019.00234.1

[50] Yusuf AS Mahmud MR Alfin DJ Gana SI Timothy S Nwaribe EE . Clinical characteristics and challenges of management of traumatic spinal cord injury in a trauma center of a developing country. J Neurosci Rural Pract. (2019) 10:393–9. doi: 10.1055/s-0039-1695696 31595109 PMC6779583

[51] Jakimovska VM Biering-Sørensen F Lidal IB Kostovski E. Epidemiological characteristics and early complications after spinal cord injury in former Yugoslav Republic of Macedonia. Spinal Cord. (2020) 58:86–94. doi: 10.1038/s41393-019-0342-9, PMID: 31427697 PMC7223761

[52] Nade ES Andriessen MV Rimoy F Maendeleo M Saria V Moshi HI . Intermittent catheterisation for individuals with disability related to spinal cord injury in Tanzania. Spinal Cord Ser Cases. (2020) 6:66. doi: 10.1038/s41394-020-0316-3, PMID: 32719337 PMC7385170

[53] Baniya M Kitrungrote L Damkliang J. Prevalence, severity, and self-management of depressive mood among community-dwelling people with spinal cord injury in Nepal. Belitung Nurs J. (2022) 8:101–7. doi: 10.33546/bnj.1991, PMID: 37521900 PMC10386813

[54] Alve YA Bontje P Begum S. Intra-and interpersonal agency: resuming occupational participation among persons with spinal cord injury after discharge from in-patient rehabilitation. Scand J Occup Ther. (2020) 27:66–79. doi: 10.1080/11038128.2019.1628298, PMID: 31230503

[55] Mahooti F Raheb G Alipour F Hatamizadeh N. Psychosocial challenges of social reintegration for people with spinal cord injury: a qualitative study. Spinal Cord. (2020) 58:1119–27. doi: 10.1038/s41393-020-0449-z, PMID: 32203067

[56] Al-Otaibi ML Almutairi KH Al-Otaibi KM Alghaeb AN Al-Hadi SH. Levels of public awareness regarding cervical spine injury and the suitable first aid response among adults in Saudi Arabia. Saudi Med J. (2021) 42:543–9. doi: 10.15537/smj.2021.42.5.20200760, PMID: 33896784 PMC9149699

[57] Pacheco Barzallo D Oña A Gemperli A. Unmet health care needs and inequality: a cross-country comparison of the situation of people with spinal cord injury. Health Serv Res. (2021) 56:1429–40. doi: 10.1111/1475-6773.13738 34386981 PMC8579205

[58] Farmahini-Farahani M Khankeh H Hosseini M Dalvandi A Tabrizi K. Excruciating care: experiences of care transition from hospital to home among the family caregivers of patients with spinal cord injury. Nurs Midwifery Stud. (2021) 10:34. doi: 10.4103/nms.nms_102_19

[59] Jha RK Gupta R. Traumatic spinal cord injury, an overview of epidemiology and Management in Vindhya Region. Indian J Public Health Res Dev. (2021) 12:304–7. doi: 10.37506/ijphrd.v12i2.14136

[60] Munakomi S Bajracharya A Gurung S Dewan M Joshi NP Mishra A . Appraisal of burden of caregivers to chronically rehabilitated patients with spinal cord injuries in a tertiary neurological Center in Nepal. Med Biomed. (2021) 1289:125–31. doi: 10.1007/5584_2020_569 32696444

[61] Odunaiya N Omosehin R Ojo J Odole A. A mixed-method study of burden of care and its associated factors among informal caregivers of individuals with spinal cord injury in Nigeria. Int J Physiother. (2021) 8:121–30. doi: 10.15621/ijphy/2021/v8i2/995

[62] Toluse AM Adeyemi TO. Epidemiology and clinical outcomes of spinal cord injuries at a level II trauma Centre in Nigeria: a longitudinal five year study. Int Orthop. (2021) 45:665–71. doi: 10.1007/s00264-020-04898-y, PMID: 33443597

[63] Zuckerman SL Haghdel A Lessing NL Carnevale J Cheserem B Lazaro A . Cervical spine trauma in East Africa: presentation, treatment, and mortality. Int J Spine Surg. (2021) 15:879–89. doi: 10.14444/8113, PMID: 34551932 PMC8651192

[64] Shah G Dhakal GR Gupta A Hamal PK Dhungana S Poudel S. Outcome of cervical spine trauma patients admitted to the intensive care unit at a tertiary government referral trauma center in Nepal. Global Spine J. (2022) 12:1388–91. doi: 10.1177/2192568220980703, PMID: 33455459 PMC9393990

[65] Smith CJ Bergene EB Tadele A Mesfin FB. A comparison of thoracolumbar injury classification in spine trauma patients among neurosurgeons in East Africa versus North America. Cureus. (2022) 14:e31761. doi: 10.7759/cureus.31761 36569733 PMC9774996

[66] Williams TL Joseph C Nilsson-Wikmar L Phillips J. Exploration of the experiences of persons in the traumatic spinal cord injury population in relation to chronic pain management. Int J Environ Res Public Health. (2022) 20:77. doi: 10.3390/ijerph20010077, PMID: 36612393 PMC9819756

[67] Sertkaya Z Koyuncu E Nakipoğlu Yüzer GF Özgirgin N. Investigation of health literacy level and its effect on quality of life in patients with spinal cord injury. J Spinal Cord Med. (2023) 46:62–7. doi: 10.1080/10790268.2021.1991162, PMID: 34726584 PMC9897774

[68] Zanini C Amann J Brach M Gemperli A Rubinelli S. The challenges characterizing the lived experience of caregiving. A qualitative study in the field of spinal cord injury. Spinal Cord. (2021) 59:493–503. doi: 10.1038/s41393-021-00618-4, PMID: 33742117 PMC8110474

[69] AlSaleh AJ Qureshi AZ Abdin ZS AlHabter AM. Long-term compliance with bladder management in patients with spinal cord injury: a Saudi-Arabian perspective. J Spinal Cord Med. (2020) 43:374–9. doi: 10.1080/10790268.2018.1531609, PMID: 30346256 PMC7241560

[70] Tharu NS Alam M Bajracharya S Chaudhary GP Pandey J Kabir MA. Caregivers’ knowledge, attitude, and practice towards pressure injuries in spinal cord injury at rehabilitation center in Bangladesh. Adv Orthopedics. (2022) 2022:1–9. doi: 10.1155/2022/8642900, PMID: 35747167 PMC9213162

[71] Ashipala DO Langendorf L. Experiences of spinal cord injury patients admitted to the rehabilitation unit at the national referral hospital in Khomas region, Namibia. Afr J Disabil. (2022) 11:1018. doi: 10.4102/ajod.v11i0.1018 35936925 PMC9350479

[72] Badenhorst M Verhagen E Lambert M van Mechelen W Brown J. Accessing healthcare as a person with a rugby-related spinal cord injury in South Africa: the injured player’s perspective. Physiother Theory Pract. (2022) 38:1639–55. doi: 10.1080/09593985.2021.1872753 33491535

[73] Höfers W Jørgensen V Sällström S Vege KM Strøm M New PW . Organisation of services and systems of care in paediatric spinal cord injury rehabilitation in seven countries: a survey with a descriptive cross-sectional design. Spinal Cord. (2022) 60:339–47. doi: 10.1038/s41393-021-00726-1, PMID: 34802054

[74] Dorjbal D Zanini C Tsegmid N Stucki G Rubinelli S. Toward an optimization of rehabilitation services for persons with spinal cord injury in Mongolia: the perspective of medical doctors. Disabil Rehabil. (2021) 43:2200–12. doi: 10.1080/09638288.2019.1696415, PMID: 31790290

[75] Shrestha S Shrestha K Groves CC. Patient handling and transportation from site of injury to tertiary trauma centres in Nepal following acute traumatic spinal cord injury: a descriptive study. Spinal Cord Ser Cases. (2022) 8:79. doi: 10.1038/s41394-022-00545-3, PMID: 36088345 PMC9464210

[76] Azadmanjir Z Mohtasham-Amiri Z Ziabari S-M Kochakinejad L Haidari H Mohseni M . Sustaining the national spinal cord injury registry of Iran (NSCIR-IR) in a regional center: challenges and solutions. Iran J Public Health. (2020) 49:736–43. doi: 10.18502/ijph.v49i4.3181, PMID: 32548054 PMC7283190

[77] Kumprou M Thaweewannakij T Arayawichanon P Amatachaya P Amatachaya S. External devices among individuals with spinal cord injury from a developing country. Am J Phys Med Rehabil. (2021) 100:952–7. doi: 10.1097/PHM.0000000000001676 33394593

[78] Kuzu D Perrin PB Pugh M Jr. Spinal cord injury/disorder function, affiliate stigma, and caregiver burden in Turkey. PM&R. (2021) 13:1376–84. doi: 10.1002/pmrj.12548, PMID: 33400847

[79] Leidinger A Kim EE Navarro-Ramirez R Rutabasibwa N Msuya SR Askin G . Spinal trauma in Tanzania: current management and outcomes. J Neurosurg Spine. (2019) 31:103–11. doi: 10.3171/2018.12.SPINE18635, PMID: 30952133

[80] Lessing NL Lazaro A Zuckerman SL Leidinger A Rutabasibwa N Shabani HK . Nonoperative treatment of traumatic spinal injuries in Tanzania: who is not undergoing surgery and why? Spinal Cord. (2020) 58:1197–205. doi: 10.1038/s41393-020-0474-y, PMID: 32350408 PMC7222864

[81] Liu H Hossain MS Islam MS Rahman MA Costa PD Herbert RD . Understanding how a community-based intervention for people with spinal cord injury in Bangladesh was delivered as part of a randomised controlled trial: a process evaluation. Spinal Cord. (2020) 58:1166–75. doi: 10.1038/s41393-020-0495-6, PMID: 32541882 PMC7606133

[82] Magogo J Lazaro A Mango M Zuckerman SL Leidinger A Msuya S . Operative treatment of traumatic spinal injuries in Tanzania: surgical management, neurologic outcomes, and time to surgery. Global Spine J. (2021) 11:89–98. doi: 10.1177/2192568219894956 32875835 PMC7734258

[83] Mansoor SN Rathore FA. Bladder management practices in spinal cord injury patients: a single center experience from a developing country. J Spinal Cord Med. (2019) 42:786–90. doi: 10.1080/10790268.2017.1417803, PMID: 29323623 PMC6830287

[84] Oliveira FG Dutra F Resende RA Mancini MC Sampaio RF. Spinal cord injury and work challenges: an analysis of paid work status and pathways of return to work in Brazil. Spinal Cord. (2021) 59:1111–9. doi: 10.1038/s41393-021-00637-1, PMID: 33972700

[85] Barzallo DP Gross-Hemmi M Bickenbach J Juocevičius A Popa D Wahyuni LK . Quality of life and the health system: a 22-country comparison of the situation of people with spinal cord injury. Arch Phys Med Rehabil. (2020) 101:2167–76. doi: 10.1016/j.apmr.2020.04.030 32533934

[86] Paulus-Mokgachane TMM Visagie SJ Mji G. Access to primary care for persons with spinal cord injuries in the greater Gaborone area, Botswana. Afr J Disabil. (2019) 8:539. doi: 10.4102/ajod.v8i0.539 31616623 PMC6779981

[87] Mohammadi F Oshvandi K Bijani M Borzou SR Khodaveisi M Masoumi SZ. Perception of facing life's challenges in patients with spinal cord injury in Iran: a qualitative study. BMC Psychol. (2022) 10:202. doi: 10.1186/s40359-022-00909-2, PMID: 35971169 PMC9376906

[88] Foongchomcheay A Eitivipart AC Kespichayawattana J Muangngoen M. Quality of life after spinal cord injury in Thai individuals: a mixed-methods study. Hong Kong Physiother J. (2019) 39:35–55. doi: 10.1142/S1013702519500045, PMID: 31156316 PMC6467828

[89] Shabany M NikbakhtNasrabadi A Mohammadi N Pruitt SD. Family-centered empowerment process in individuals with spinal cord injury living in Iran: a grounded theory study. Spinal Cord. (2020) 58:174–84. doi: 10.1038/s41393-019-0348-3, PMID: 31477808

[90] Visser AM Visagie S. Pressure ulcer knowledge, beliefs and practices in a group of South Africans with spinal cord injury. Spinal Cord Ser Cases. (2019) 5:83. doi: 10.1038/s41394-019-0226-4, PMID: 31700681 PMC6821773

[91] Yang Y Gong Z Reinhardt JD Xu G Xu Z Li J. Environmental barriers and participation restrictions in community-dwelling individuals with spinal cord injury in Jiangsu and Sichuan provinces of China: results from a cross-sectional survey. J Spinal Cord Med. (2023) 46:277–90. doi: 10.1080/10790268.2021.1935094, PMID: 34139132 PMC9987748

[92] Chhabra H Sharma S Arora M. Challenges in comprehensive management of spinal cord injury in India and in the Asian spinal cord network region: findings of a survey of experts, patients and consumers. Spinal Cord. (2018) 56:71–7. doi: 10.1038/sc.2017.102, PMID: 28895578

[93] Löfvenmark I Norrbrink C Nilsson-Wikmar L Hultling C Chakandinakira S Hasselberg M. Traumatic spinal cord injury in Botswana: characteristics, aetiology and mortality. Spinal Cord. (2015) 53:150–4. doi: 10.1038/sc.2014.203, PMID: 25420494

[94] Sharma S Sivakami M. “God will decide her fate”: the trajectories of women with traumatic spinal cord injury in India. Disabil Rehabil. (2023) 45:2003–12. doi: 10.1080/09638288.2022.2083245, PMID: 35654780

[95] Hossain MS Islam MS Rahman MA Glinsky JV Herbert RD Ducharme S . Health status, quality of life and socioeconomic situation of people with spinal cord injuries six years after discharge from a hospital in Bangladesh. Spinal Cord. (2019) 57:652–61. doi: 10.1038/s41393-019-0261-9, PMID: 30787428

[96] Uddin T Shakoor M Rathore FA Sakel M. Ethical issues and dilemmas in spinal cord injury rehabilitation in the developing world: a mixed-method study. Spinal Cord. (2022) 60:882–7. doi: 10.1038/s41393-022-00808-8, PMID: 35523952

[97] Pilusa SI Myezwa H Potterton J. Services and interventions needed to prevent secondary health conditions throughout the life span of people with spinal cord injury, South Africa. Afr J Disabil. (2022) 11:1–8. doi: 10.4102/ajod.v11i0.881 PMC9724123 36483844

[98] Gowinnage SS Wicramabahu Senarath Paranayapa P Arambepola C. Sexual and reproductive health experiences, knowledge and associations: a neglected issue among adults with spinal cord injury in Sri Lanka. Sex Disabil. (2022) 40:687–700. doi: 10.1007/s11195-022-09758-8

[99] Pilusa S Myezwa H Potterton J. ‘I forget to do pressure relief’: personal factors influencing the prevention of secondary health conditions in people with spinal cord injury, South Africa. South Afr J Physiother. (2021) 77:1493. doi: 10.4102/sajp.v77i1.1493, PMID: 33824916 PMC8008043

[100] Pilusa S Myezwa H Potterton J. Views of health care users and providers: solutions to improve the prevention of secondary health conditions among people with spinal cord injury, South Africa. Spinal Cord Ser Cases. (2022) 8:67. doi: 10.1038/s41394-022-00530-w, PMID: 35853865 PMC9296448

[101] Pilusa S Myezwa H Potterton J. Environmental factors influencing the prevention of secondary health conditions among people with spinal cord injury, South Africa. PLoS One. (2021) 16:e0252280. doi: 10.1371/journal.pone.0252280, PMID: 34170928 PMC8232458

[102] Tarighi P Tabibi SJ Motevalian SA Tofighi S Maleki MR Panahi F . Designing a model for trauma system management using public health approach: the case of Iran. Acta Med Iran. (2012) 50:9–17. 22267372

[103] Alghnam S AlSayyari A Albabtain I Aldebasi B Alkelya M. Long-term disabilities after traumatic head injury (THI): a retrospective analysis from a large level-I trauma center in Saudi Arabia. Inj Epidemiol. (2017) 4:1–8. doi: 10.1186/s40621-017-0126-7 29090361 PMC5663989

[104] Noonan VK Fingas M Farry A Baxter D Singh A Fehlings MG . Incidence and prevalence of spinal cord injury in Canada: a national perspective. Neuroepidemiology. (2012) 38:219–26. doi: 10.1159/000336014 22555590

[105] Bökel A Egen C Gutenbrunner C Weidner N Moosburger J Abel FR . Spinal cord injury in Germany – a survey on the living and care situation of people with spinal cord injury. Rehabilitation. (2020) 59:205–13. doi: 10.1055/a-1071-5935, PMID: 31962349

[106] Thomson N. Emergency medical services in Zimbabwe. Resuscitation. (2005) 65:15–9. doi: 10.1016/j.resuscitation.2005.01.008 15797271

[107] Bal A Cooper M Lee A Anil M Hennes H. The evaluation of trauma care: the comparison of 2 high-level pediatric emergency departments in the United States and Turkey. Pediatr Emerg Care. (2019) 35:611–7. doi: 10.1097/PEC.0000000000001110 28419017

[108] Masmejean EH Faye A Alnot J-Y Mignon AF. Trauma care systems in France. Injury. (2003) 34:669–73. doi: 10.1016/S0020-1383(03)00146-3 12951291

[109] Morrissey BE Delaney RA Johnstone AJ Petrovick L Smith RM. Do trauma systems work? A comparison of major trauma outcomes between Aberdeen Royal Infirmary and Massachusetts General Hospital. Injury. (2015) 46:150–5. doi: 10.1016/j.injury.2014.08.048, PMID: 25270693

[110] Sadek A-R Damian M Eynon CA. The role of neurosciences intensive care in neurological conditions. Br J Hosp Med. (2005) 74:558–63. doi: 10.12968/hmed.2013.74.10.558 24105308

[111] McLane HC Berkowitz AL Patenaude BN McKenzie ED Wolper E Wahlster S . Availability, accessibility, and affordability of neurodiagnostic tests in 37 countries. Neurology. (2015) 85:1614–22. doi: 10.1212/WNL.0000000000002090, PMID: 26446063 PMC4642148

[112] Pilusa S Myezwa H Potterton J. Secondary health conditions in people with spinal cord injury in South Africa: prevalence and associated factors. S Afr Med J. (2021) 111:1211–7. doi: 10.7196/SAMJ.2021.v111i12.15761, PMID: 34949309

[113] Alve YA Bontje P. Factors influencing participation in daily activities by persons with spinal cord injury: lessons learned from an international scoping review. Topics Spinal Cord Injury Rehabil. (2019) 25:41–61. doi: 10.1310/sci2501-41, PMID: 30774289 PMC6368111

[114] Wisborg T Montshiwa TR Mock C. Trauma research in low-and middle-income countries is urgently needed to strengthen the chain of survival. Scand J Trauma Resusc Emerg Med. (2011) 19:62–5. doi: 10.1186/1757-7241-19-62 22024376 PMC3219714

[115] Perel P Ker K Ivers R Blackhall K. Road safety in low-and middle-income countries: a neglected research area. Inj Prev. (2007) 13:227. doi: 10.1136/ip.2007.016527 17686930 PMC2598339

[116] Bishai DM Hyder AA. Modeling the cost effectiveness of injury interventions in lower and middle income countries: opportunities and challenges. Cost Effectiveness Resource Allocation. (2006) 4:1–11. doi: 10.1186/1478-7547-4-2 16423285 PMC1379660

[117] Gururaj G. Road traffic deaths, injuries and disabilities in India: current scenario. Natl Med J India. (2008) 21:14–20. PMID: 18472698

[118] Punjani NS Shams S Bhanji SM. Analysis of health care delivery systems: Pakistan versus United States. Int J Endorsing Health Sci Res. (2014) 2:38–41. doi: 10.29052/IJEHSR.v2.i1.2014.38-41

[119] Lee B Cripps RA Fitzharris M Wing P. The global map for traumatic spinal cord injury epidemiology: update 2011, global incidence rate. Spinal Cord. (2014) 52:110–6. doi: 10.1038/sc.2012.158, PMID: 23439068

[120] Staton C Vissoci J Gong E Toomey N Wafula R Abdelgadir J . Road traffic injury prevention initiatives: a systematic review and metasummary of effectiveness in low and middle income countries. PLoS One. (2016) 11:e0144971. doi: 10.1371/journal.pone.0144971, PMID: 26735918 PMC4703343

[121] Peden MM. World report on road traffic injury prevention. World Health Organization. (2004).

[122] Doecke SD Baldock MR Kloeden CN Dutschke JK. Impact speed and the risk of serious injury in vehicle crashes. Accid Anal Prev. (2020) 144:105629. doi: 10.1016/j.aap.2020.105629, PMID: 32570088

[123] Khorasani-Zavareh D. System versus traditional approach in road traffic injury prevention: a call for action. J Injury Violence Res. (2011) 3:1. doi: 10.5249/jivr.v3i2.128 PMC3134927 21498966

[124] Selveindran SM Khan MM Simadibrata DM Hutchinson PJ Brayne C Hill C . Mapping global evidence on strategies and interventions in neurotrauma and road traffic collisions prevention: a scoping review protocol. BMJ Open. (2019) 9:e031517. doi: 10.1136/bmjopen-2019-031517, PMID: 31722947 PMC6858136

[125] Bickenbach J Officer A Shakespeare T von Groote P. The international spinal cord S. International perspectives on spinal cord injury: summary. Geneva: World Health Organization (2013) Contract No.: WHO/NMH/VIP/13.03.

[126] Burns AS O'Connell C. The challenge of spinal cord injury care in the developing world. J Spinal Cord Med. (2012) 35:3–8. doi: 10.1179/2045772311Y.0000000043 22330185 PMC3240914

[127] Orach DCG. Health equity: challenges in low income countries. Afr Health Sci. (2009) 9:S49–51. 20589106 PMC2877288

[128] Montazeri A. Road-traffic-related mortality in Iran: a descriptive study. Public Health. (2004) 118:110–3. doi: 10.1016/S0033-3506(03)00173-2, PMID: 15037040

[129] Soori H Royanian M Zali A Movahedinejad A. Road traffic injuries in Iran: the role of interventions implemented by traffic police. Traffic Inj Prev. (2009) 10:375–8. doi: 10.1080/15389580902972579, PMID: 19593716

[130] De Silva MJ Roberts I Perel P Edwards P Kenward MG Fernandes J . Patient outcome after traumatic brain injury in high-, middle-and low-income countries: analysis of data on 8927 patients in 46 countries. Int J Epidemiol. (2009) 38:452–8. doi: 10.1093/ije/dyn189 18782898

[131] Bhattarai HK Bhusal S Barone-Adesi F Hubloue I. Prehospital emergency care in low-and middle-income countries: a systematic review. Prehosp Disaster Med. (2023) 38:495–512. doi: 10.1017/S1049023X23006088 37492946 PMC10445116

[132] Rubiano AM Clavijo A. Neurotrauma registries in low-and middle-income countries for building organized neurotrauma care: the LATINO registry experience: comment on “neurotrauma surveillance in national registries of low-and middle-income countries: a scoping review and comparative analysis of data dictionaries”. Int J Health Policy Manag. (2023) 12:7505. doi: 10.34172/ijhpm.2022.7505 36028976 PMC10125183

[133] Dijkink S Nederpelt CJ Krijnen P Velmahos GC Schipper IB. Trauma systems around the world: a systematic overview. J Trauma Acute Care Surg. (2017) 83:917–25. doi: 10.1097/TA.0000000000001633, PMID: 28715361

[134] Pakkanen T Kämäräinen A Huhtala H Silfvast T Nurmi J Virkkunen I . Physician-staffed helicopter emergency medical service has a beneficial impact on the incidence of prehospital hypoxia and secured airways on patients with severe traumatic brain injury. Scand J Trauma Resusc Emerg Med. (2017) 25:1–7. doi: 10.1186/s13049-017-0438-1 28915898 PMC5603088

[135] Mock C. Guidelines for essential trauma care. World Health Organization. (2004).

[136] Endo A Kojima M Uchiyama S Shiraishi A Otomo Y. Physician-led prehospital management is associated with reduced mortality in severe blunt trauma patients: a retrospective analysis of the Japanese nationwide trauma registry. Scand J Trauma Resusc Emerg Med. (2021) 29:1–8. doi: 10.1186/s13049-020-00828-4 33407748 PMC7789566

[137] Zhang L-Y Zhang X-Z Bai X-J Zhang M Zhao X-G Xu Y-A . Current trauma care system and trauma care training in China. Chin J Traumatol. (2018) 21:73–6. doi: 10.1016/j.cjtee.2017.07.005, PMID: 29395429 PMC5911734

[138] Missios S Bekelis K. Nonmedical factors and the transfer of spine trauma patients initially evaluated at level III and IV trauma centers. Spine J. (2015) 15:2028–35. doi: 10.1016/j.spinee.2015.05.017, PMID: 25998327

[139] Rathore FA New PW Iftikhar A. A report on disability and rehabilitation medicine in Pakistan: past, present, and future directions. Arch Phys Med Rehabil. (2011) 92:161–6. doi: 10.1016/j.apmr.2010.10.004 21187218

[140] Abdollahi S Faramarzi M Delavar MA Bakouei F Chehrazi M Gholinia H. Effect of psychotherapy on reduction of fear of childbirth and pregnancy stress: a randomized controlled trial. Front Psychol. (2020) 11:787. doi: 10.3389/fpsyg.2020.00787, PMID: 32528340 PMC7265090

[141] Servadei F Rossini Z Nicolosi F Morselli C Park KB. The role of neurosurgery in countries with limited facilities: facts and challenges. World Neurosurg. (2018) 112:315–21. doi: 10.1016/j.wneu.2018.01.047, PMID: 29366998

[142] Rubiano AM Griswold DP Adelson PD Echeverri RA Khan AA Morales S . International neurotrauma training based on north-south collaborations: results of an inter-institutional program in the era of global neurosurgery. Front Surg. (2021) 8:633774. doi: 10.3389/fsurg.2021.633774, PMID: 34395505 PMC8358677

[143] Varma A Hill EG Nicholas J Selassie A. Predictors of early mortality after traumatic spinal cord injury: a population-based study. Spine. (2010) 35:778–83. doi: 10.1097/BRS.0b013e3181ba1359, PMID: 20228715

[144] Shibahashi K Nishida M Okura Y Hamabe Y. Epidemiological state, predictors of early mortality, and predictive models for traumatic spinal cord injury: a multicenter nationwide cohort study. Spine. (2019) 44:479–87. doi: 10.1097/BRS.0000000000002871 30234810

[145] Hejrati N Moghaddamjou A Pedro K Alvi MA Harrop JS Guest JD . Current practice of acute spinal cord injury management: a global survey of members from the AO spine. Global Spine J. (2024) 14:546–60. doi: 10.1177/21925682221116888, PMID: 36036628 PMC10802552

[146] Adhikari NK Fowler RA Bhagwanjee S Rubenfeld GD. Critical care and the global burden of critical illness in adults. Lancet. (2010) 376:1339–46. doi: 10.1016/S0140-6736(10)60446-1, PMID: 20934212 PMC7136988

[147] Zimmerman A Fox S Griffin R Nelp T Thomaz EBAF Mvungi M . An analysis of emergency care delays experienced by traumatic brain injury patients presenting to a regional referral hospital in a low-income country. PLoS One. (2020) 15:e0240528. doi: 10.1371/journal.pone.0240528, PMID: 33045030 PMC7549769

[148] Chang AM Cohen DJ Lin A Augustine J Handel DA Howell E . Hospital strategies for reducing emergency department crowding: a mixed-methods study. Ann Emerg Med. (2018) 71:497–505.e4. doi: 10.1016/j.annemergmed.2017.07.022 28844764 PMC5828915

[149] Al-Naami MY Arafah MA Al-Ibrahim FS. Trauma care systems in Saudi Arabia: an agenda for action. Ann Saudi Med. (2010) 30:50–8. doi: 10.5144/0256-4947.59374, PMID: 20103958 PMC2850182

[150] Joshipura M. Trauma care in India: current scenario. World J Surg. (2008) 32:1613–7. doi: 10.1007/s00268-008-9634-5, PMID: 18553048

[151] Zargar M Motamedi SMRK Karbakhsh M Ghodsi SM Rahimi-Movaghar V Panahi F . Trauma care system in Iran. Chinese J Traumatol. (2011) 14:131–6. doi: 10.3760/cma.j.issn.1008-1275.2011.03.001 21635797

[152] Meara JG Leather AJ Hagander L Alkire BC Alonso N Ameh EA . Global surgery 2030: evidence and solutions for achieving health, welfare, and economic development. Lancet. (2015) 386:569–624. doi: 10.1016/S0140-6736(15)60160-X 25924834

[153] Maroufi SF Azadnajafabad S Pour-Rashidi A Jazayeri SB Ghodsi Z Ghawami H . Adopting and adapting clinical practice guidelines for timing of decompressive surgery in acute spinal cord injury from a developed world context to a developing region. Acta Neurochir. (2023) 165:1401–6. doi: 10.1007/s00701-023-05591-w, PMID: 37074391

[154] Richardson A Samaranayaka A Sullivan M Derrett S. Secondary health conditions and disability among people with spinal cord injury: a prospective cohort study. J Spinal Cord Med. (2021) 44:19–28. doi: 10.1080/10790268.2019.1581392, PMID: 30882288 PMC7919890

[155] Hug K Stumm C Debecker I Fellinghauer CS Peter C Hund-Georgiadis M. Self-efficacy and pressure ulcer prevention after spinal cord injury—results from a nationwide community survey in Switzerland (SwiSCI). PM&R. (2018) 10:573–86. doi: 10.1016/j.pmrj.2017.11.017, PMID: 29225161

[156] Groves C Poudel M Baniya M Rana C House D. Descriptive study of earthquake-related spinal cord injury in Nepal. Spinal Cord. (2017) 55:705–10. doi: 10.1038/sc.2017.25, PMID: 28290470

[157] Furlan AD Irvin E Munhall C Giraldo-Prieto M Fullerton L McMaster R . Rehabilitation service models for people with physical and/or mental disability living in low-and middle-income countries: a systematic review. J Rehabil Med. (2018) 50:487–98. doi: 10.2340/16501977-2325, PMID: 29616278

[158] Abedi A Biering-Sørensen F Chhabra HS D’Andréa Greve JM Khan NM Koskinen E . An international survey of the structure and process of care for traumatic spinal cord injury in acute and rehabilitation facilities: lessons learned from a pilot study. BMC Health Serv Res. (2022) 22:1565. doi: 10.1186/s12913-022-08847-w 36544168 PMC9768992

[159] Brown DS Nell V. Epidemiology of traumatic brain injury in Johannesburg—I. Methodological issues in a developing country context. Soc Sci Med. (1991) 33:283–7. doi: 10.1016/0277-9536(91)90362-G, PMID: 1925692

[160] Rezaei M Sharifi A Vaccaro A Rahimi-Movaghar V. Home-based rehabilitation programs: promising field to maximize function of patients with traumatic spinal cord injury. Asian J Neurosurg. (2019) 14:634–40. doi: 10.4103/ajns.AJNS_86_17 31497079 PMC6703054

[161] Eslami V Saadat S Habibi Arejan R Vaccaro A Ghodsi S Rahimi-Movaghar V. Factors associated with the development of pressure ulcers after spinal cord injury. Spinal Cord. (2012) 50:899–903. doi: 10.1038/sc.2012.75 22777490

[162] Ghodsi Z Jazayeri SB Pourrashidi A Sadeghi-Naeini M Azadmanjir Z Baigi V . Development of a comprehensive assessment tool to measure the quality of care for individuals with traumatic spinal cord injuries. Spinal Cord Ser Cases. (2023) 9:12. doi: 10.1038/s41394-023-00569-3, PMID: 37005413 PMC10067818

[163] Derakhshan P Miller WC Bundon A Labbé D Bolt T Mortenson WB. Adaptive outdoor physical activities for adults with mobility disability: a scoping review. Front Rehabil Sci. (2024) 4:1331971. doi: 10.3389/fresc.2023.1331971, PMID: 38259872 PMC10801018

[164] Rahman S. The perception of people with spinal cord injury on modified home environment. World J Advan Res Rev. (2018) 18:376–83. doi: 10.30574/wjarr.2023.18.1.0584

[165] Krause JS. Employment after spinal cord injury. Arch Phys Med Rehabil. (1992) 73:163–9. PMID: 1543412

[166] Tate DG Stiers W Daugherty J Forchheimer M Cohen E Hansen N. The effects of insurance benefits coverage on functional and psychosocial outcomes after spinal cord injury. Arch Phys Med Rehabil. (1994) 75:407–14. doi: 10.1016/0003-9993(94)90164-3 8172500

[167] Jongbloed L Backman C Forwell SJ Carpenter C. Employment after spinal cord injury: the impact of government policies in Canada. Work. (2007) 29:145–54. PMID: 17726290

[168] O’Neill J Dyson-Hudson TA. Employment after spinal cord injury. Curr Phys Med Rehabil Rep. (2020) 8:141–8. doi: 10.1007/s40141-020-00266-4

[169] Hossain MS Harvey LA Islam MS Rahman MA Liu H Herbert RD. Loss of work-related income impoverishes people with SCI and their families in Bangladesh. Spinal Cord. (2020) 58:423–9. doi: 10.1038/s41393-019-0382-1 31772346 PMC7138756

[170] Tangcharoensathien V Witthayapipopsakul W Viriyathorn S Patcharanarumol W. Improving access to assistive technologies: challenges and solutions in low-and middle-income countries. WHO South East Asia J Public Health. (2018) 7:84–9. doi: 10.4103/2224-3151.239419, PMID: 30136666

