# Supplementary material for: Challenges in traumatic spinal cord injury care in developing countries – a scoping review
Source: Front Public Health. 2024 Aug 19;12:1377513. doi: 10.3389/fpubh.2024.1377513 (PMC11368135; doi:10.3389/fpubh.2024.1377513)
Supplement: Supplementary file 1 [file Data_Sheet_1.docx]

**Appendix A-** **Electronic search strategy for different databases.**

| Database | Search strategy |
| --- | --- |
| PubMed | (TSCI [TIAB] OR ( ("Spinal Cord"[Mesh] OR "Spinal Cord Injuries"[Mesh] OR SCI[TIAB] OR Myelopath* [TIAB] OR (lateral*[TIAB] AND (cornu[TIAB] OR horn[TIAB] )) OR (spinal[TIAB] AND marrow[TIAB] ) OR spinalcord*[TIAB] OR Myelon*[TIAB] OR (Medulla[TIAB] AND Spinal*[TIAB]) OR (Conus[TIAB] AND (Medullari* [TIAB] OR Terminali*[TIAB])) OR ((spine[TIAB] OR spinal*[tiab] OR Lumbar [TIAB] OR Thoracic[TIAB] OR Coccygeal[TIAB] OR Sacral[TIAB] ) AND Cord*[TIAB]) ) AND ("Wounds and Injuries"[Mesh] OR Injur*[TIAB] OR reinjury[TIAB] OR Trauma*[TIAB] OR Transection* [TIAB] OR Laceration*[TIAB] OR Contusion*[TIAB] OR fracture*[TIAB] OR wound*[TIAB] OR damag*[TIAB] OR hemisection* [TIAB] OR compress*[TIAB] OR Broken [TIAB] OR break*[TIAB] OR "Spinal Injuries"[Mesh] ) ) ) AND  (Care [TIAB] OR cares[TIAB] OR management*[TIAB] OR "Disease Management"[Mesh] ) AND  ("Developing Countries"[Mesh] OR ( (Developing[TIAB] OR (Least[TIAB] AND Developed[TIAB] ) OR (Less[TIAB] AND Developed[TIAB] ) OR Under-Developed[TIAB] OR underdeveloped[TIAB] OR Third-World[TIAB] ) AND (Nation*[TIAB] OR Countr*[TIAB] OR Area[TIAB] ) ) OR Armenia*[TIAB] OR "Armenia"[Mesh] OR "Azerbaijan"[Mesh] OR Azerbaijan* [TIAB] OR Belarus* [TIAB] OR Byelarus*[TIAB] OR Byelorussia*[TIAB] OR Belorussia*[TIAB] OR Georgia*[TIAB] OR "Georgia (Republic)"[Mesh] OR "Republic of Belarus"[Mesh] OR “White Russia” [TIAB] OR Kazakhstan*[TIAB] OR Kazakh [TIAB] OR "Kazakhstan"[Mesh] OR Kyrgyz [TIAB] OR "Kyrgyzstan"[Mesh] OR Kirghizia [TIAB] OR Kirghiz [TIAB] OR Kirgizstan [TIAB] OR Kazak [TIAB] OR Moldova* [TIAB] OR Moldavia*[TIAB] OR Moldavian[TIAB] OR "Moldova"[Mesh] OR Russia [TIAB] OR Russian[TIAB] OR "Russia"[Mesh] OR Siberia [TIAB] OR Tajikistan* [TIAB] OR "Tajikistan"[Mesh] OR Tadjikistan*[TIAB] OR Tadzhik*[TIAB] OR Tadzhikistan*[TIAB] OR Turkmenistan* [TIAB] OR "Turkmenistan"[Mesh] OR Turkmen*[TIAB] OR Turkmenia[TIAB] OR Turkmenian[TIAB] OR Ukraine* [TIAB] OR "Ukraine"[Mesh] OR Ukraina[TIAB] OR Ukrainian [TIAB] OR Uzbekistan* [TIAB] OR "Uzbekistan"[Mesh] OR Uzbek*[TIAB] OR Bangladesh* [TIAB] OR "Bangladesh"[Mesh] OR “east Pakistan” [TIAB] OR Bhutan* [TIAB] OR "Bhutan"[Mesh] OR Brunei*[TIAB] OR "Brunei"[Mesh] OR Cambodia* [TIAB] OR "Cambodia"[Mesh] OR Khmer[TIAB] OR Kampuchea*[TIAB] OR kampudja*[TIAB] OR China [TIAB] OR "China"[Mesh] OR Manchuria*[TIAB] OR PRC[TIAB] OR Beijing [TIAB] OR Chinese[TIAB] OR Sinkiang*[TIAB] OR “Inner Mongolia” [TIAB] OR Fiji* [TIAB] OR "Fiji"[Mesh] OR Indian* [TIAB] OR India [TIAB] OR "India"[Mesh] OR Indonesia [TIAB] OR "Indonesia"[Mesh] OR “East Indies”[TIAB] OR “West Irian” [TIAB] OR Indonesian[TIAB] OR (“New Guinea” [TIAB] AND West[TIAB] ) OR ( rian[TIAB] Jaya [TIAB] Timor[TIAB]) OR Java[TIAB] OR Bali[TIAB] OR Sumatra[TIAB] OR Celebes[TIAB] OR Sulawesi[TIAB] OR (Malay[TIAB] Archipelago[TIAB] ) OR Madoera[TIAB] OR Madura[TIAB] OR Kiribati [TIAB] OR "Micronesia"[Mesh] OR Micronesia*[TIAB] OR “Gilbert Islands” [TIAB] OR “Mariana Islands”[TIAB] OR “Marshall Islands”[TIAB] OR Nauru[TIAB] OR “Pacific Islands” [TIAB] OR Tuvalu[TIAB] OR “Ellice Islands” [TIAB] OR “Caroline Islands” [TIAB] OR Micronesia*[TIAB] OR Lao [TIAB] OR LAOS*[TIAB] OR "Laos"[Mesh] OR Malaysia* [TIAB] OR "Malaysia"[Mesh] OR Malaya*[TIAB] OR Malay*[TIAB] OR Sabah[TIAB] OR Sarawak[TIAB] OR “North Borneo” [TIAB] OR Maldives[TIAB] OR "Indian Ocean Islands"[Mesh] OR Pemba[TIAB] OR Cocos[TIAB] OR "Micronesia"[Mesh] OR “Johnston Island” [TIAB] OR “Gilbert Islands”[TIAB] OR “Mariana Islands” [TIAB] OR Nauru[TIAB] OR Tuvalu[TIAB] OR “Caroline Islands”[TIAB] OR Micronesia [TIAB] OR Mongolia* [TIAB] OR "Mongolia"[Mesh] OR Mongolian [TIAB] OR Myanmar* [TIAB] OR "Myanmar"[Mesh] OR Myanma*[TIAB] OR Burma[TIAB] OR “Pleasant Island” [TIAB] OR Nepal* [TIAB] OR "Nepal"[Mesh] OR Palau*[TIAB] OR "Palau"[Mesh] OR Belau[TIAB] OR Palaos[TIAB] OR Pelew[TIAB] OR Papua [TIAB] OR (“New Guinea”[TIAB] East[TIAB] ) OR "Papua New Guinea"[Mesh] OR PNG [TIAB] OR Philippines [TIAB] OR Phillipines[TIAB] OR "Philippines"[Mesh] OR Samoa* [TIAB] OR "Samoa"[Mesh] OR Samoan[TIAB] OR “Navigator Island”[TIAB] OR “Navigator Islands” [TIAB] OR “Solomon Islands” [TIAB] OR "Melanesia"[Mesh] OR “Norfolk Island“[TIAB] OR “Sri Lanka” [TIAB] OR "Sri Lanka"[Mesh] OR Ceylon[TIAB] OR Thailand* [TIAB] OR "Thailand"[Mesh] OR Siam [TIAB] OR "Timor-Leste"[Mesh] OR Timor [TIAB] OR Tonga*[TIAB] OR "Tonga"[Mesh] OR Tuvalu [TIAB] OR “Ellice Islands” [TIAB] OR Vanuatu [TIAB] OR "Vanuatu"[Mesh] OR “New Hebrides” [TIAB] OR Vietnam*[TIAB] OR "Vietnam"[Mesh] OR “ Viet Nam” [TIAB] OR Albania* [TIAB] OR "Albania"[Mesh] OR Bosnia*[TIAB] OR "Bosnia and Herzegovina"[Mesh] OR Bulgaria* [TIAB] OR Bulgarian[TIAB] OR Bulgarians[TIAB] OR "Bulgaria"[Mesh] OR Croatia* [TIAB] OR "Croatia"[Mesh] OR Hungary*[TIAB] OR "Hungary"[Mesh] OR Kosovo* [TIAB] OR "Kosovo"[Mesh] OR Montenegro* [TIAB] OR "Montenegro"[Mesh] OR “North Macedonia”[TIAB] OR "Macedonia (Republic)"[Mesh] OR Macedonia*[TIAB] OR Yugoslavia[TIAB] OR Poland* [TIAB] OR "Poland"[Mesh] OR Romania* [TIAB] OR "Romania"[Mesh] OR Rumania*[TIAB] OR Roumania*[TIAB] OR Serbia [TIAB] OR "Serbia"[Mesh] OR Turkey*[TIAB] OR "Turkey"[Mesh] OR Barbuda* [TIAB] OR "Antigua and Barbuda"[Mesh] OR Antigua*[TIAB] OR Argentina*[TIAB] OR Argentine*[TIAB] OR "Argentina"[Mesh] OR Aruba* [TIAB] OR "Aruba"[Mesh] OR bahama*[TIAB] OR Bahamas* [TIAB] OR "Bahamas"[Mesh] OR Barbados* [TIAB] OR "Barbados"[Mesh] OR Belize* [TIAB] OR "Belize"[Mesh] OR “British Honduras” [TIAB] OR Bolivia* [TIAB] OR "Bolivia"[Mesh] OR Brazil* [TIAB] OR "Brazil"[Mesh] OR Chile* [TIAB] OR "Chile"[Mesh] OR chili*[TIAB] OR Colombia* [TIAB] OR "Colombia"[Mesh] OR “Costa Rica” [TIAB] OR "Costa Rica"[Mesh] OR Dominica* [TIAB] OR "Dominica"[Mesh] OR Dominican[TIAB] OR Ecuador* [TIAB] OR "Ecuador"[Mesh] OR “Galapagos Islands” [TIAB] OR Salvador* [TIAB] OR "El Salvador"[Mesh] OR Grenada* [TIAB] OR "Grenada"[Mesh] OR Guatemala* [TIAB] OR "Guatemala"[Mesh] OR Guyana* [TIAB] OR "Guyana"[Mesh] OR Guiana[TIAB] OR Haiti*[TIAB] OR "Haiti"[Mesh] OR Hayti* [TIAB] OR Honduras* [TIAB] OR "Honduras"[Mesh] OR Jamaica* [TIAB] OR "Jamaica"[Mesh] OR Mexico [TIAB] OR "Mexico"[Mesh] OR Mexican[TIAB] OR Nicaragua* [TIAB] OR "Nicaragua"[Mesh] OR Panama* [TIAB] OR "Panama"[Mesh] OR Paraguay* [TIAB] OR "Paraguay"[Mesh] OR Peru* [TIAB] OR "Peru"[Mesh] OR Nevis [TIAB] OR "Saint Kitts and Nevis"[Mesh] OR Lucia [TIAB] OR "Saint Lucia"[Mesh] OR Vincent [TIAB] OR Grenadines[TIAB] OR "Saint Vincent and the Grenadines"[Mesh] OR Surinam*[TIAB] OR Suriname* [TIAB] OR "Suriname"[Mesh] OR “Dutch Guiana” [TIAB] OR “Netherlands Guiana” [TIAB] OR Trinidad [TIAB] OR Tobago* [TIAB] OR "Trinidad and Tobago"[Mesh] OR Uruguay* [TIAB] OR "Uruguay"[Mesh] [TIAB] OR Venezuela* [TIAB] OR "Venezuela"[Mesh] OR Afghanistan*[TIAB] OR "Afghanistan"[Mesh] OR Algeria*[TIAB] OR "Algeria"[Mesh] OR Bahrain*[TIAB] OR "Bahrain"[Mesh] OR Djibouti*[TIAB] OR "Djibouti"[Mesh] OR “French Somaliland” [TIAB] OR “afar and issa land” [TIAB] OR “french sudan” [TIAB] OR Egypt* [TIAB] OR "Egypt"[Mesh] OR “united arab republic” [TIAB] OR Iran*[TIAB] OR "Iran"[Mesh] OR Persia*[TIAB] OR Iraq*[TIAB] OR "Iraq"[Mesh] OR Jordan* [TIAB] OR "Jordan"[Mesh] OR transjordan[TIAB] OR Kuwait* [TIAB] OR "Kuwait"[Mesh] OR Lebanon* [TIAB] OR "Lebanon"[Mesh] OR Lebanese [TIAB] OR libia*[TIAB] OR Libya* [TIAB] OR "Libya"[Mesh] OR Mauritania* [TIAB] OR "Mauritania"[Mesh] OR Morocco* [TIAB] OR "Morocco"[Mesh] OR Ifni[TIAB] OR Oman* [TIAB] OR "Oman"[Mesh] OR Muscat*[TIAB] OR Pakistan* [TIAB] OR "Pakistan"[Mesh] OR Qatar* [TIAB] OR "Qatar"[Mesh] OR Katar*[TIAB] OR Quatar*[TIAB] OR (Saudi*[TIAB] Arabia [TIAB]) OR "Saudi Arabia"[Mesh] OR Somalia* [TIAB] OR "Somalia"[Mesh] OR Sudan* [TIAB] OR "Sudan"[Mesh] OR Syria*[TIAB] OR "Syria"[Mesh] OR Syrian[TIAB] OR Tunisia* [TIAB] OR "Tunisia"[Mesh] OR tunesia*[TIAB] OR UAE[TIAB] OR Emirates [TIAB] OR "United Arab Emirates"[Mesh] OR “Trucial States” [TIAB] OR “Abu Dhabi” [TIAB] OR Yemen* [TIAB] OR "Yemen"[Mesh] OR Sanaa[TIAB] OR Aden[TIAB] OR Angola* [TIAB] OR "Angola"[Mesh] OR Benin* [TIAB] OR "Benin"[Mesh] OR Dahomey*[TIAB] OR Botswana* [TIAB] OR "Botswana"[Mesh] OR Bechuanaland[TIAB] OR Kalahari[TIAB] OR (Burkina[TIAB] Faso [TIAB]) OR "Burkina Faso"[Mesh] OR “Upper Volta” [TIAB] OR Burundi* [TIAB] OR "Burundi"[Mesh] OR Urundi[TIAB] OR (Cabo [TIAB] Verde [TIAB]) OR "Cabo Verde"[Mesh] OR “Cape Verde” [TIAB] OR Cameroon* [TIAB] OR "Cameroon"[Mesh] OR Cameroons*[TIAB] OR Cameron*[TIAB] OR cameroun*[TIAB] OR "Central African Republic"[Mesh] OR Ubangi-Shari[TIAB] OR Centrafrican [TIAB] OR Centrafrique [TIAB] OR (Central [TIAB] African [TIAB]) OR Chad* [TIAB] OR "Chad"[Mesh] OR Tchad[TIAB] OR Comoros*[TIAB] OR "Comoros"[Mesh] OR “comoro islands “[TIAB] OR “Comoro Mayotte”[TIAB] OR Congo* [TIAB] OR "Congo"[Mesh] OR “Côte d’Ivoire” [TIAB] OR "Cote d'Ivoire"[Mesh] OR “Cote diIvoire” [TIAB] OR “ Ivory Coast” [TIAB] OR (Equatorial[TIAB] Guinea* [TIAB]) OR "Equatorial Guinea"[Mesh] OR “rio muni “[TIAB] OR “Spanish Guinea” [TIAB] OR “Rio Muni” [TIAB] OR Eritrea* [TIAB] OR "Eritrea"[Mesh] OR Eswatini [TIAB] OR Swaziland* [TIAB] OR "Swaziland"[Mesh] OR Ethiopia* [TIAB] OR "Ethiopia"[Mesh] OR Ethiopians[TIAB] OR Gabon* [TIAB] OR "Gabon"[Mesh] OR Gabonese[TIAB] OR gaboon[TIAB] OR Gambia* [TIAB] OR "Gambia"[Mesh] OR Ghana* [TIAB] OR "Ghana"[Mesh] OR “Gold Coast” [TIAB] OR Guinea [TIAB] OR "Guinea"[Mesh] OR Guinea-Bissau [TIAB] OR "Guinea-Bissau"[Mesh] OR “Portuguese Guinea” [TIAB] OR Kenya* [TIAB] OR "Kenya"[Mesh] OR Lesotho* [TIAB] OR "Lesotho"[Mesh] OR Basutoland[TIAB] OR Liberia* [TIAB] OR "Liberia"[Mesh] OR Madagascar* [TIAB] OR Madagascan*[TIAB] OR Madagascans*[TIAB] OR "Madagascar"[Mesh] OR Malagasy[TIAB] OR Malawi* [TIAB] OR "Malawi"[Mesh] OR Nyasaland[TIAB] OR Mali [TIAB] OR "Mali"[Mesh] OR Mauritius* [TIAB] OR "Mauritius"[Mesh] OR “ Agalega Islands” [TIAB] OR Mozambique*[TIAB] OR "Mozambique"[Mesh] OR (Portuguese[TIAB] “East Africa” [TIAB] ) OR Namibia* [TIAB] OR "Namibia"[Mesh] OR “Southwest Africa” [TIAB] OR ( South[TIAB] “West Africa” [TIAB] ) OR Niger* [TIAB] OR "Niger"[Mesh] OR Nigeria* [TIAB] OR "Nigeria"[Mesh] OR Rwanda[TIAB] OR "Rwanda"[Mesh] OR Ruanda*[TIAB] OR “São Tomé and Príncipe “[TIAB] OR "Sao Tome and Principe"[Mesh] OR Senegal* [TIAB] OR "Senegal"[Mesh] OR Seychelles* [TIAB] OR "Seychelles"[Mesh] OR Sierra*[TIAB] OR Leone [TIAB] OR Salone [TIAB] OR "Sierra Leone"[Mesh] OR “South Africa” [TIAB] OR "South Africa"[Mesh] OR RSA[TIAB] OR “South Sudan”[TIAB] OR "South Sudan"[Mesh] OR Tanzania* [TIAB] OR "Tanzania"[Mesh] OR Zanzibar*[TIAB] OR Tanganyika[TIAB] OR Togo* [TIAB] OR Togolese*[TIAB] OR "Togo"[Mesh] OR Uganda* [TIAB] OR "Uganda"[Mesh] OR Zambia* [TIAB] OR "Zambia"[Mesh] OR “Northern Rhodesia” [TIAB] OR Zimbabwe*[TIAB] OR "Zimbabwe"[Mesh] OR Rhodesia[TIAB] ) |
| Scopus | ( ( TSCI OR ( ( SCI OR Myelopath* OR (lateral* AND (cornu OR horn )) OR (spinal AND marrow ) OR spinalcord* OR Myelon* OR (Medulla AND Spinal*) OR (Conus AND (Medullari* OR Terminali*)) OR ((spine OR spinal* OR Lumbar OR Thoracic OR Coccygeal OR Sacral ) AND Cord*) )   AND (Injur* OR reinjury OR Trauma* OR Transection* OR Laceration* OR Contusion* OR fracture* OR wound* OR damag* OR hemisection* OR compress* OR Broken OR break* ) ) ) AND  (Care OR cares OR management*) AND ( ( (Developing OR (Least AND Developed ) OR (Less AND Developed ) OR Under-Developed OR underdeveloped OR Third-World ) AND (Nation* OR Countr* OR Area) ) OR Armenia* OR Azerbaijan* OR Belarus* OR Byelarus* OR Byelorussia* OR Belorussia* OR Georgia* OR “White Russia” OR Kazakhstan* OR Kazakh OR Kyrgyz OR Kirghizia OR Kirghiz OR Kirgizstan OR Kazak OR Moldova* OR Moldavia* OR Moldavian OR Russia OR Russian OR Siberia OR Tajikistan* OR Tadjikistan* OR Tadzhik* OR Tadzhikistan* OR Turkmenistan* OR Turkmen* OR Turkmenia OR Turkmenian OR Ukraine* OR Ukraina OR Ukrainian OR Uzbekistan* OR Uzbek* OR Bangladesh* OR “east Pakistan” OR Bhutan* OR Brunei* OR Cambodia* OR Khmer OR Kampuchea* OR kampudja* OR China OR Manchuria* OR PRC OR Beijing OR Chinese OR Sinkiang* OR “Inner Mongolia” OR Fiji* OR Indian* OR India OR Indonesia OR “East Indies” OR “West Irian” OR Indonesian OR (“New Guinea” AND West ) OR ( rian Jaya Timor) OR Java OR Bali OR Sumatra OR Celebes OR Sulawesi OR (Malay Archipelago ) OR Madoera OR Madura OR Kiribati OR Micronesia* OR “Gilbert Islands” OR “Mariana Islands” OR “Marshall Islands” OR Nauru OR “Pacific Islands” OR Tuvalu OR “Ellice Islands” OR “Caroline Islands” OR Micronesia* OR Lao OR LAOS* OR Malaysia* OR Malaya* OR Malay* OR Sabah OR Sarawak OR “North Borneo” OR Maldives OR Pemba OR Cocos OR “Johnston Island” OR “Gilbert Islands” OR “Mariana Islands” OR Nauru OR Tuvalu OR “Caroline Islands” OR Micronesia OR Mongolia* OR Mongolian OR Myanmar* OR Myanma* OR Burma OR “Pleasant Island” OR Nepal* OR Palau* OR Belau OR Palaos OR Pelew OR Papua OR (“New Guinea” East ) OR PNG OR Philippines OR Phillipines OR Samoa* OR Samoan OR “Navigator Island” OR “Navigator Islands” OR “Solomon Islands” OR “Norfolk Island“ OR “Sri Lanka” OR Ceylon OR Thailand* OR Siam OR Timor OR Tonga* OR Tuvalu OR “Ellice Islands” OR Vanuatu OR “New Hebrides” OR Vietnam* OR “ Viet Nam” OR Albania* OR Bosnia* OR Bulgaria* OR Bulgarian OR Bulgarians OR Croatia* OR Hungary* OR Kosovo* OR Montenegro* OR “North Macedonia” OR Macedonia* OR Yugoslavia OR Poland* OR Romania* OR Rumania* OR Roumania* OR Serbia OR Turkey* OR Barbuda* OR Antigua* OR Argentina* OR Argentine* OR Aruba* OR bahama* OR Bahamas* OR Barbados* OR Belize* OR “British Honduras” OR Bolivia* OR Brazil* OR Chile* OR chili* OR Colombia* OR “Costa Rica” OR Dominica* OR Dominican OR Ecuador* OR “Galapagos Islands” OR Salvador* OR Grenada* OR Guatemala* OR Guyana* OR Guiana OR Haiti* OR Hayti* OR Honduras* OR Jamaica* OR Mexico OR Mexican OR Nicaragua* OR Panama* OR Paraguay* OR Peru* OR Nevis OR Lucia OR Vincent OR Grenadines OR Surinam* OR Suriname* OR “Dutch Guiana” OR “Netherlands Guiana” OR Trinidad OR Tobago* OR Uruguay* OR Venezuela* OR Afghanistan* OR Algeria* OR Bahrain* OR “French Somaliland” OR “afar and issa land” OR “french sudan” OR Egypt* OR “united arab republic” OR Iran* OR Persia* OR Iraq* OR Jordan* OR transjordan OR Kuwait* OR Lebanon* OR Lebanese OR libia* OR Libya* OR Mauritania* OR Morocco* OR Ifni OR Oman* OR Muscat* OR Pakistan* OR Qatar* OR Katar* OR Quatar* OR (Saudi* Arabia) OR Somalia* OR Sudan* OR Syria* OR Syrian OR Tunisia* OR tunesia* OR UAE OR Emirates OR “Trucial States” OR “Abu Dhabi” OR Yemen* OR Sanaa OR Aden OR Angola* OR Benin* OR Dahomey* OR Botswana* OR Bechuanaland OR Kalahari OR (Burkina Faso) OR “Upper Volta” OR Burundi* OR Urundi OR (Cabo Verde) OR “Cape Verde” OR Cameroon* OR Cameroons* OR Cameron* OR cameroun* OR Ubangi-Shari OR Centrafrican OR Centrafrique OR (Central African ) OR Chad* OR Tchad OR Comoros* OR “comoro islands “ OR “Comoro Mayotte” OR Congo* OR “Côte d’Ivoire” OR “Cote diIvoire” OR “ Ivory Coast” OR (Equatorial Guinea*) OR “rio muni “ OR “Spanish Guinea” OR “Rio Muni” OR Eritrea* OR Eswatini OR Swaziland* OR Ethiopia* OR Ethiopians OR Gabon* OR Gabonese OR gaboon OR Gambia* OR Ghana* OR “Gold Coast” OR Guinea OR Guinea-Bissau OR “Portuguese Guinea” OR Kenya* OR Lesotho* OR Basutoland OR Liberia* OR Madagascar* OR Madagascan* OR Madagascans* OR Malagasy OR Malawi* OR Nyasaland OR Mali OR Mauritius* OR “ Agalega Islands” OR Mozambique* OR (Portuguese “East Africa”) OR Namibia* OR “Southwest Africa” OR ( South “West Africa”) OR Niger* OR Nigeria* OR Rwanda OR Ruanda* OR “São Tomé and Príncipe “ OR Senegal* OR Seychelles* OR Sierra* OR Leone OR Salone OR “South Africa” OR RSA OR “South Sudan” OR Tanzania* OR Zanzibar* OR Tanganyika OR Togo* OR Togolese* OR Uganda* OR Zambia* OR “Northern Rhodesia” OR Zimbabwe* OR Rhodesia)) |
| Web of Science | TS=( ( TSCI OR ( ( SCI OR Myelopath* OR (lateral* AND (cornu OR horn )) OR (spinal AND marrow ) OR spinalcord* OR Myelon* OR (Medulla AND Spinal*) OR (Conus AND (Medullari* OR Terminali*)) OR ((spine OR spinal* OR Lumbar OR Thoracic OR Coccygeal OR Sacral ) AND Cord*) )   AND (Injur* OR reinjury OR Trauma* OR Transection* OR Laceration* OR Contusion* OR fracture* OR wound* OR damag* OR hemisection* OR compress* OR Broken OR break* ) ) ) AND  (Care OR cares OR management*)  AND ( ( (Developing OR (Least AND Developed ) OR (Less AND Developed ) OR Under-Developed OR underdeveloped OR Third-World ) AND (Nation* OR Countr* OR Area ) ) OR Armenia* OR Azerbaijan* OR Belarus* OR Byelarus* OR Byelorussia* OR Belorussia* OR Georgia* OR "White Russia" OR Kazakhstan* OR Kazakh OR Kyrgyz OR Kirghizia OR Kirghiz OR Kirgizstan OR Kazak OR Moldova* OR Moldavia* OR Moldavian OR Russia OR Russian OR Siberia OR Tajikistan* OR Tadjikistan* OR Tadzhik* OR Tadzhikistan* OR Turkmenistan* OR Turkmen* OR Turkmenia OR Turkmenian OR Ukraine* OR Ukraina OR Ukrainian OR Uzbekistan* OR Uzbek* OR Bangladesh* OR "east Pakistan" OR Bhutan* OR Brunei* OR Cambodia* OR Khmer OR Kampuchea* OR kampudja* OR China OR Manchuria* OR PRC OR Beijing OR Chinese OR Sinkiang* OR "Inner Mongolia" OR Fiji* OR Indian* OR India OR Indonesia OR "East Indies" OR "West Irian" OR Indonesian OR ("New Guinea" AND West ) OR ( rian Jaya Timor) OR Java OR Bali OR Sumatra OR Celebes OR Sulawesi OR (Malay Archipelago ) OR Madoera OR Madura OR Kiribati OR Micronesia* OR "Gilbert Islands" OR "Mariana Islands" OR "Marshall Islands" OR Nauru OR "Pacific Islands" OR Tuvalu OR "Ellice Islands" OR "Caroline Islands" OR Micronesia* OR Lao OR LAOS* OR Malaysia* OR Malaya* OR Malay* OR Sabah OR Sarawak OR "North Borneo" OR Maldives OR Pemba OR Cocos OR "Johnston Island" OR "Gilbert Islands" OR "Mariana Islands" OR Nauru OR Tuvalu OR "Caroline Islands" OR Micronesia OR Mongolia* OR Mongolian OR Myanmar* OR Myanma* OR Burma OR "Pleasant Island" OR Nepal* OR Palau* OR Belau OR Palaos OR Pelew OR Papua OR ("New Guinea" East ) OR PNG OR Philippines OR Phillipines OR Samoa* OR Samoan OR "Navigator Island" OR "Navigator Islands" OR "Solomon Islands" OR "Norfolk Island" OR "Sri Lanka" OR Ceylon OR Thailand* OR Siam OR Timor OR Tonga* OR Tuvalu OR "Ellice Islands" OR Vanuatu OR "New Hebrides" OR Vietnam* OR " Viet Nam" OR Albania* OR Bosnia* OR Bulgaria* OR Bulgarian OR Bulgarians OR Croatia* OR Hungary* OR Kosovo* OR Montenegro* OR "North Macedonia" OR Macedonia* OR Yugoslavia OR Poland* OR Romania* OR Rumania* OR Roumania* OR Serbia OR Turkey* OR Barbuda* OR Antigua* OR Argentina* OR Argentine* OR Aruba* OR bahama* OR Bahamas* OR Barbados* OR Belize* OR "British Honduras" OR Bolivia* OR Brazil* OR Chile* OR chili* OR Colombia* OR "Costa Rica" OR Dominica* OR Dominican OR Ecuador* OR "Galapagos Islands" OR Salvador* OR Grenada* OR Guatemala* OR Guyana* OR Guiana OR Haiti* OR Hayti* OR Honduras* OR Jamaica* OR Mexico OR Mexican OR Nicaragua* OR Panama* OR Paraguay* OR Peru* OR Nevis OR Lucia OR Vincent OR Grenadines OR Surinam* OR Suriname* OR "Dutch Guiana" OR "Netherlands Guiana" OR Trinidad OR Tobago* OR Uruguay* OR Venezuela* OR Afghanistan* OR Algeria* OR Bahrain* OR "French Somaliland" OR "afar and issa land" OR "french sudan" OR Egypt* OR "united arab republic" OR Iran* OR Persia* OR Iraq* OR Jordan* OR transjordan OR Kuwait* OR Lebanon* OR Lebanese OR libia* OR Libya* OR Mauritania* OR Morocco* OR Ifni OR Oman* OR Muscat* OR Pakistan* OR Qatar* OR Katar* OR Quatar* OR (Saudi* Arabia) OR Somalia* OR Sudan* OR Syria* OR Syrian OR Tunisia* OR tunesia* OR UAE OR Emirates OR "Trucial States" OR "Abu Dhabi" OR Yemen* OR Sanaa OR Aden OR Angola* OR Benin* OR Dahomey* OR Botswana* OR Bechuanaland OR Kalahari OR (Burkina Faso) OR "Upper Volta" OR Burundi* OR Urundi OR (Cabo Verde) OR "Cape Verde" OR Cameroon* OR Cameroons* OR Cameron* OR cameroun* OR Ubangi-Shari OR Centrafrican OR Centrafrique OR (Central African ) OR Chad* OR Tchad OR Comoros* OR "comoro islands " OR "Comoro Mayotte" OR Congo* OR "Côte dIvoire" OR "Cote diIvoire" OR " Ivory Coast" OR (Equatorial Guinea*) OR "rio muni " OR "Spanish Guinea" OR "Rio Muni" OR Eritrea* OR Eswatini OR Swaziland* OR Ethiopia* OR Ethiopians OR Gabon* OR Gabonese OR gaboon OR Gambia* OR Ghana* OR "Gold Coast" OR Guinea OR Guinea-Bissau OR "Portuguese Guinea" OR Kenya* OR Lesotho* OR Basutoland OR Liberia* OR Madagascar* OR Madagascan* OR Madagascans* OR Malagasy OR Malawi* OR Nyasaland OR Mali OR Mauritius* OR " Agalega Islands" OR Mozambique* OR (Portuguese "East Africa") OR Namibia* OR "Southwest Africa" OR ( South "West Africa") OR Niger* OR Nigeria* OR Rwanda OR Ruanda* OR "São Tomé and Príncipe " OR Senegal* OR Seychelles* OR Sierra* OR Leone OR Salone OR "South Africa" OR RSA OR "South Sudan" OR Tanzania* OR Zanzibar* OR Tanganyika OR Togo* OR Togolese* OR Uganda* OR Zambia* OR "Northern Rhodesia" OR Zimbabwe* OR Rhodesia)) |
| Embase | (TSCI:ab,ti,kw OR ( ( 'spinal cord'/exp OR 'spinal cord injury'/exp OR SCI:ab,ti,kw OR Myelopath*:ab,ti,kw OR (lateral*:ab,ti,kw AND (cornu:ab,ti,kw OR horn:ab,ti,kw )) OR (spinal:ab,ti,kw AND marrow:ab,ti,kw ) OR spinalcord*:ab,ti,kw OR Myelon*:ab,ti,kw OR (Medulla:ab,ti,kw AND Spinal*:ab,ti,kw ) OR (Conus:ab,ti,kw AND (Medullari*:ab,ti,kw OR Terminali*:ab,ti,kw )) OR ((spine:ab,ti,kw OR spinal*:ab,ti,kw OR Lumbar:ab,ti,kw OR Thoracic:ab,ti,kw OR Coccygeal:ab,ti,kw OR Sacral:ab,ti,kw ) AND Cord*:ab,ti,kw ) ) AND ( 'injury'/exp OR 'spine injury'/exp OR Injur*:ab,ti,kw OR reinjury:ab,ti,kw OR Trauma*:ab,ti,kw OR Transection*:ab,ti,kw OR Laceration*:ab,ti,kw OR Contusion*:ab,ti,kw OR fracture*:ab,ti,kw OR wound*:ab,ti,kw OR damag*:ab,ti,kw OR hemisection*:ab,ti,kw OR compress*:ab,ti,kw OR Broken:ab,ti,kw OR break*:ab,ti,kw ) ) ) AND (Care:ab,ti,kw OR cares:ab,ti,kw OR management*:ab,ti,kw OR 'management'/exp ) AND ('developing country'/exp OR ( (Developing:ab,ti,kw OR (Least:ab,ti,kw AND Developed:ab,ti,kw ) OR (Less:ab,ti,kw AND Developed:ab,ti,kw ) OR Under-Developed:ab,ti,kw OR underdeveloped:ab,ti,kw OR Third-World:ab,ti,kw ) AND (Nation*:ab,ti,kw OR Countr*:ab,ti,kw OR Area:ab,ti,kw ) ) OR 'Armenia'/exp OR 'Azerbaijan'/exp OR 'Russian Federation'/exp OR 'Moldova'/exp OR 'Kazakhstan'/exp OR 'Belarus'/exp OR 'georgia'/exp OR 'Kyrgyzstan'/exp OR 'Tajikistan'/exp OR 'Turkmenistan'/exp OR 'Ukraine'/exp OR 'Uzbekistan'/exp OR 'Bangladesh'/exp OR 'Bhutan'/exp OR 'Brunei Darussalam'/exp OR 'Cambodia'/exp OR 'China'/exp OR 'India'/exp OR 'Indonesia'/exp OR 'Federated States of Micronesia'/exp OR 'Laos'/exp OR 'Malaysia'/exp OR 'Maldives'/exp OR 'Federated States of Micronesia'/exp OR 'Mongolia'/exp OR 'Myanmar'/exp OR 'Nepal'/exp OR 'Palau'/exp OR 'Papua New Guinea'/exp OR 'Philippines'/exp OR 'Samoa'/exp OR 'Melanesia'/exp OR 'Sri Lanka'/exp OR 'Thailand'/exp OR 'Timor-Leste'/exp OR 'Tonga'/exp OR 'Vanuatu'/exp OR 'Viet Nam'/exp OR 'Albania'/exp OR 'Bulgarian (people)'/exp OR 'Bosnia and Herzegovina'/exp OR 'Croatia'/exp OR 'Hungary'/exp OR 'Kosovo'/exp OR 'Montenegro (republic)'/exp OR 'Macedonia (republic)'/exp OR 'Poland'/exp OR 'Romania'/exp OR 'Serbia'/exp OR 'turkey'/exp OR 'Antigua and Barbuda'/exp OR 'Argentina'/exp OR 'Aruba'/exp OR 'Bahamas'/exp OR 'Barbados'/exp OR 'Belize'/exp OR 'Bolivia'/exp OR 'Brazil'/exp OR 'Chile'/exp OR 'Colombia'/exp OR 'Costa Rica'/exp OR 'Dominica'/exp OR 'Ecuador'/exp OR 'El Salvador'/exp OR 'Grenada'/exp OR 'Guatemala'/exp OR 'Guyana'/exp OR 'Haiti'/exp OR 'Honduras'/exp OR 'Jamaica'/exp OR 'Mexico'/exp OR 'Nicaragua'/exp OR 'Panama'/exp OR 'Paraguay'/exp OR 'Peru'/exp OR 'Saint Kitts and Nevis'/exp OR 'Saint Lucia'/exp OR 'Saint Vincent and the Grenadines'/exp OR 'Suriname'/exp OR 'Trinidad and Tobago'/exp OR 'Uruguay'/exp OR 'Venezuela'/exp OR 'Afghanistan'/exp OR 'Algeria'/exp OR 'Bahrain'/exp OR 'Djibouti'/exp OR 'Egypt'/exp OR 'Iran'/exp OR 'Iraq'/exp OR 'Jordan'/exp OR 'Kuwait'/exp OR 'Lebanon'/exp OR 'Libyan Arab Jamahiriya'/exp OR 'Mauritania'/exp OR 'Morocco'/exp OR 'Oman'/exp OR 'Pakistan'/exp OR 'Qatar'/exp OR 'Saudi Arabia'/exp OR 'Somalia'/exp OR 'Sudan'/exp OR 'Syrian Arab Republic'/exp OR 'Tunisia'/exp OR 'United Arab Emirates'/exp OR 'Yemen'/exp OR 'Angola'/exp OR 'Benin'/exp OR 'Botswana'/exp OR 'Burkina Faso'/exp OR 'Burundi'/exp OR 'Cape Verde'/exp OR 'Cameroon'/exp OR 'Central African Republic'/exp OR 'Chad'/exp OR 'Comoros'/exp OR 'Congo'/exp OR 'Cote d`Ivoire'/exp OR 'Equatorial Guinea'/exp OR 'Eritrea'/exp OR 'Swaziland'/exp OR 'Ethiopian'/exp OR 'Gabon'/exp OR 'Gambia'/exp OR 'Ghana'/exp OR 'Guinea'/exp OR 'Guinea-Bissau'/exp OR 'Kenya'/exp OR 'Lesotho'/exp OR 'Liberia'/exp OR 'Malagasy (citizen)'/exp OR 'Malawi'/exp OR 'Mali'/exp OR 'Mauritius'/exp OR 'Mozambique'/exp OR 'Namibia'/exp OR 'Niger'/exp OR 'Nigeria'/exp OR 'Rwanda'/exp OR 'Sao Tome and Principe'/exp OR 'Senegal'/exp OR 'Seychelles'/exp OR 'Sierra Leone'/exp OR 'South Africa'/exp OR 'South Sudan'/exp OR 'Togo'/exp OR 'Uganda'/exp OR 'Zambia'/exp OR 'Zimbabwe'/exp OR Armenia*:ab,ti,kw OR Azerbaijan*:ab,ti,kw OR Belarus*:ab,ti,kw OR Byelarus*:ab,ti,kw OR Byelorussia*:ab,ti,kw OR Belorussia*:ab,ti,kw OR Georgia*:ab,ti,kw OR “White Russia”:ab,ti,kw OR Kazakhstan*:ab,ti,kw OR Kazakh:ab,ti,kw OR Kyrgyz:ab,ti,kw OR Kirghizia:ab,ti,kw OR Kirghiz:ab,ti,kw OR Kirgizstan:ab,ti,kw OR Kazak:ab,ti,kw OR Moldova*:ab,ti,kw OR Moldavia*:ab,ti,kw OR Moldavian:ab,ti,kw OR Russia:ab,ti,kw OR Russian:ab,ti,kw OR Siberia:ab,ti,kw OR Tajikistan*:ab,ti,kw OR Tadjikistan*:ab,ti,kw OR Tadzhik*:ab,ti,kw OR Tadzhikistan*:ab,ti,kw OR Turkmenistan*:ab,ti,kw OR Turkmen*:ab,ti,kw OR Turkmenia:ab,ti,kw OR Turkmenian:ab,ti,kw OR Ukraine*:ab,ti,kw OR Ukraina:ab,ti,kw OR Ukrainian:ab,ti,kw OR Uzbekistan*:ab,ti,kw OR Uzbek*:ab,ti,kw OR Bangladesh*:ab,ti,kw OR “east Pakistan”:ab,ti,kw OR Bhutan*:ab,ti,kw OR Brunei*:ab,ti,kw OR Cambodia*:ab,ti,kw OR Khmer:ab,ti,kw OR Kampuchea*:ab,ti,kw OR kampudja*:ab,ti,kw OR China:ab,ti,kw OR Manchuria*:ab,ti,kw OR PRC:ab,ti,kw OR Beijing:ab,ti,kw OR Chinese:ab,ti,kw OR Sinkiang*:ab,ti,kw OR “Inner Mongolia”:ab,ti,kw OR Fiji*:ab,ti,kw OR Indian*:ab,ti,kw OR India:ab,ti,kw OR Indonesia:ab,ti,kw OR “East Indies”:ab,ti,kw OR “West Irian”:ab,ti,kw OR Indonesian:ab,ti,kw OR (“New Guinea”:ab,ti,kw AND West:ab,ti,kw ) OR ( rian:ab,ti,kw Jaya:ab,ti,kw Timor:ab,ti,kw) OR Java:ab,ti,kw OR Bali:ab,ti,kw OR Sumatra:ab,ti,kw OR Celebes:ab,ti,kw OR Sulawesi:ab,ti,kw OR (Malay:ab,ti,kw Archipelago:ab,ti,kw ) OR Madoera:ab,ti,kw OR Madura:ab,ti,kw OR Kiribati:ab,ti,kw OR Micronesia*:ab,ti,kw OR “Gilbert Islands”:ab,ti,kw OR “Mariana Islands”:ab,ti,kw OR “Marshall Islands”:ab,ti,kw OR Nauru:ab,ti,kw OR “Pacific Islands”:ab,ti,kw OR Tuvalu:ab,ti,kw OR “Ellice Islands”:ab,ti,kw OR “Caroline Islands”:ab,ti,kw OR Micronesia*:ab,ti,kw OR Lao:ab,ti,kw OR LAOS*:ab,ti,kw OR Malaysia*:ab,ti,kw OR Malaya*:ab,ti,kw OR Malay*:ab,ti,kw OR Sabah:ab,ti,kw OR Sarawak:ab,ti,kw OR “North Borneo”:ab,ti,kw OR Maldives:ab,ti,kw OR Pemba:ab,ti,kw OR Cocos:ab,ti,kw OR “Johnston Island”:ab,ti,kw OR “Gilbert Islands”:ab,ti,kw OR “Mariana Islands”:ab,ti,kw OR Nauru:ab,ti,kw OR Tuvalu:ab,ti,kw OR “Caroline Islands”:ab,ti,kw OR Micronesia:ab,ti,kw OR Mongolia*:ab,ti,kw OR Mongolian:ab,ti,kw OR Myanmar*:ab,ti,kw OR Myanma*:ab,ti,kw OR Burma:ab,ti,kw OR “Pleasant Island”:ab,ti,kw OR Nepal*:ab,ti,kw OR Palau*:ab,ti,kw OR Belau:ab,ti,kw OR Palaos:ab,ti,kw OR Pelew:ab,ti,kw OR Papua:ab,ti,kw OR (“New Guinea”:ab,ti,kw East:ab,ti,kw ) OR PNG:ab,ti,kw OR Philippines:ab,ti,kw OR Phillipines:ab,ti,kw OR Samoa*:ab,ti,kw OR Samoan:ab,ti,kw OR “Navigator Island”:ab,ti,kw OR “Navigator Islands”:ab,ti,kw OR “Solomon Islands”:ab,ti,kw OR “Norfolk Island“:ab,ti,kw OR “Sri Lanka”:ab,ti,kw OR Ceylon:ab,ti,kw OR Thailand*:ab,ti,kw OR Siam:ab,ti,kw OR Timor:ab,ti,kw OR Tonga*:ab,ti,kw OR Tuvalu:ab,ti,kw OR “Ellice Islands”:ab,ti,kw OR Vanuatu:ab,ti,kw OR “New Hebrides”:ab,ti,kw OR Vietnam*:ab,ti,kw OR “ Viet Nam”:ab,ti,kw OR Albania*:ab,ti,kw OR Bosnia*:ab,ti,kw OR Bulgaria*:ab,ti,kw OR Bulgarian:ab,ti,kw OR Bulgarians:ab,ti,kw OR Croatia*:ab,ti,kw OR Hungary*:ab,ti,kw OR Kosovo*:ab,ti,kw OR Montenegro*:ab,ti,kw OR “North Macedonia”:ab,ti,kw OR Macedonia*:ab,ti,kw OR Yugoslavia:ab,ti,kw OR Poland*:ab,ti,kw OR Romania*:ab,ti,kw OR Rumania*:ab,ti,kw OR Roumania*:ab,ti,kw OR Serbia:ab,ti,kw OR Turkey*:ab,ti,kw OR Barbuda*:ab,ti,kw OR Antigua*:ab,ti,kw OR Argentina*:ab,ti,kw OR Argentine*:ab,ti,kw OR Aruba*:ab,ti,kw OR bahama*:ab,ti,kw OR Bahamas*:ab,ti,kw OR Barbados*:ab,ti,kw OR Belize*:ab,ti,kw OR “British Honduras”:ab,ti,kw OR Bolivia*:ab,ti,kw OR Brazil*:ab,ti,kw OR Chile*:ab,ti,kw OR chili*:ab,ti,kw OR Colombia*:ab,ti,kw OR “Costa Rica”:ab,ti,kw OR Dominica*:ab,ti,kw OR Dominican:ab,ti,kw OR Ecuador*:ab,ti,kw OR “Galapagos Islands”:ab,ti,kw OR Salvador*:ab,ti,kw OR Grenada*:ab,ti,kw OR Guatemala*:ab,ti,kw OR Guyana*:ab,ti,kw OR Guiana:ab,ti,kw OR Haiti*:ab,ti,kw OR Hayti*:ab,ti,kw OR Honduras*:ab,ti,kw OR Jamaica*:ab,ti,kw OR Mexico:ab,ti,kw OR Mexican:ab,ti,kw OR Nicaragua*:ab,ti,kw OR Panama*:ab,ti,kw OR Paraguay*:ab,ti,kw OR Peru*:ab,ti,kw OR Nevis:ab,ti,kw OR Lucia:ab,ti,kw OR Vincent:ab,ti,kw OR Grenadines:ab,ti,kw OR Surinam*:ab,ti,kw OR Suriname*:ab,ti,kw OR “Dutch Guiana”:ab,ti,kw OR “Netherlands Guiana”:ab,ti,kw OR Trinidad:ab,ti,kw OR Tobago*:ab,ti,kw OR Uruguay*:ab,ti,kw OR Venezuela*:ab,ti,kw OR Afghanistan*:ab,ti,kw OR Algeria*:ab,ti,kw OR Bahrain*:ab,ti,kw OR “French Somaliland”:ab,ti,kw OR “afar and issa land”:ab,ti,kw OR “french sudan”:ab,ti,kw OR Egypt*:ab,ti,kw OR “united arab republic”:ab,ti,kw OR Iran*:ab,ti,kw OR Persia*:ab,ti,kw OR Iraq*:ab,ti,kw OR Jordan*:ab,ti,kw OR transjordan:ab,ti,kw OR Kuwait*:ab,ti,kw OR Lebanon*:ab,ti,kw OR Lebanese:ab,ti,kw OR libia*:ab,ti,kw OR Libya*:ab,ti,kw OR Mauritania*:ab,ti,kw OR Morocco*:ab,ti,kw OR Ifni:ab,ti,kw OR Oman*:ab,ti,kw OR Muscat*:ab,ti,kw OR Pakistan*:ab,ti,kw OR Qatar*:ab,ti,kw OR Katar*:ab,ti,kw OR Quatar*:ab,ti,kw OR (Saudi*:ab,ti,kw Arabia:ab,ti,kw) OR Somalia*:ab,ti,kw OR Sudan*:ab,ti,kw OR Syria*:ab,ti,kw OR Syrian:ab,ti,kw OR Tunisia*:ab,ti,kw OR tunesia*:ab,ti,kw OR UAE:ab,ti,kw OR Emirates:ab,ti,kw OR “Trucial States”:ab,ti,kw OR “Abu Dhabi”:ab,ti,kw OR Yemen*:ab,ti,kw OR Sanaa:ab,ti,kw OR Aden:ab,ti,kw OR Angola*:ab,ti,kw OR Benin*:ab,ti,kw OR Dahomey*:ab,ti,kw OR Botswana*:ab,ti,kw OR Bechuanaland:ab,ti,kw OR Kalahari:ab,ti,kw OR (Burkina:ab,ti,kw Faso:ab,ti,kw) OR “Upper Volta”:ab,ti,kw OR Burundi*:ab,ti,kw OR Urundi:ab,ti,kw OR (Cabo:ab,ti,kw Verde:ab,ti,kw) OR “Cape Verde”:ab,ti,kw OR Cameroon*:ab,ti,kw OR Cameroons*:ab,ti,kw OR Cameron*:ab,ti,kw OR cameroun*:ab,ti,kw OR Ubangi-Shari:ab,ti,kw OR Centrafrican:ab,ti,kw OR Centrafrique:ab,ti,kw OR (Central:ab,ti,kw African:ab,ti,kw ) OR Chad*:ab,ti,kw OR Tchad:ab,ti,kw OR Comoros*:ab,ti,kw OR “comoro islands “:ab,ti,kw OR “Comoro Mayotte”:ab,ti,kw OR Congo*:ab,ti,kw OR “Cote dIvoire”:ab,ti,kw OR “Cote diIvoire”:ab,ti,kw OR “ Ivory Coast”:ab,ti,kw OR (Equatorial:ab,ti,kw Guinea*:ab,ti,kw) OR “rio muni “:ab,ti,kw OR “Spanish Guinea”:ab,ti,kw OR “Rio Muni”:ab,ti,kw OR Eritrea*:ab,ti,kw OR Eswatini:ab,ti,kw OR Swaziland*:ab,ti,kw OR Ethiopia*:ab,ti,kw OR Ethiopians:ab,ti,kw OR Gabon*:ab,ti,kw OR Gabonese:ab,ti,kw OR gaboon:ab,ti,kw OR Gambia*:ab,ti,kw OR Ghana*:ab,ti,kw OR “Gold Coast”:ab,ti,kw OR Guinea:ab,ti,kw OR Guinea-Bissau:ab,ti,kw OR “Portuguese Guinea”:ab,ti,kw OR Kenya*:ab,ti,kw OR Lesotho*:ab,ti,kw OR Basutoland:ab,ti,kw OR Liberia*:ab,ti,kw OR Madagascar*:ab,ti,kw OR Madagascan*:ab,ti,kw OR Madagascans*:ab,ti,kw OR Malagasy:ab,ti,kw OR Malawi*:ab,ti,kw OR Nyasaland:ab,ti,kw OR Mali:ab,ti,kw OR Mauritius*:ab,ti,kw OR “ Agalega Islands”:ab,ti,kw OR Mozambique*:ab,ti,kw OR (Portuguese:ab,ti,kw “East Africa”:ab,ti,kw) OR Namibia*:ab,ti,kw OR “Southwest Africa”:ab,ti,kw OR ( South:ab,ti,kw “West Africa”:ab,ti,kw) OR Niger*:ab,ti,kw OR Nigeria*:ab,ti,kw OR Rwanda OR Ruanda*:ab,ti,kw OR “São Tomé and Príncipe “:ab,ti,kw OR Senegal*:ab,ti,kw OR Seychelles*:ab,ti,kw OR Sierra*:ab,ti,kw OR Leone:ab,ti,kw OR Salone:ab,ti,kw OR “South Africa”:ab,ti,kw OR RSA:ab,ti,kw OR “South Sudan”:ab,ti,kw OR Tanzania*:ab,ti,kw OR Zanzibar*:ab,ti,kw OR Tanganyika:ab,ti,kw OR Togo*:ab,ti,kw OR Togolese*:ab,ti,kw OR Uganda*:ab,ti,kw OR Zambia*:ab,ti,kw OR “Northern Rhodesia”:ab,ti,kw OR Zimbabwe*:ab,ti,kw OR Rhodesia:ab,ti,kw) |
| Cochrane library | (TSCI):ti,ab,kw MeSH descriptor: [149] explode all trees MeSH descriptor: [Spinal Cord Injuries] explode all trees (( SCI OR Myelopath* OR (lateral* AND (cornu OR horn )) OR (spinal AND marrow ) OR spinalcord* OR Myelon* OR (Medulla AND Spinal*) OR (Conus AND (Medullari* OR Terminali*)) OR ((spine OR spinal* OR Lumbar OR Thoracic OR Coccygeal OR Sacral ) AND Cord*) )):ti,ab,kw #2 or #3 or #4 MeSH descriptor: [Wounds and Injuries] explode all trees MeSH descriptor: [Spinal Injuries] explode all trees ((Injur* OR reinjury OR Trauma* OR Transection* OR Laceration* OR Contusion* OR fracture* OR wound* OR damag* OR hemisection* OR compress* OR Broken OR break* )):ti,ab,kw #6 or #7 or #8 #5 and #9 #1 or #10 (( ( (Developing OR (Least AND Developed ) OR (Less AND Developed ) OR Under-Developed OR underdeveloped OR Third-World ) AND (Nation* OR Countr* OR Area ) ) OR Armenia* OR Azerbaijan* OR Belarus* OR Byelarus* OR Byelorussia* OR Belorussia* OR Georgia* OR "White Russia" OR Kazakhstan* OR Kazakh OR Kyrgyz OR Kirghizia OR Kirghiz OR Kirgizstan OR Kazak OR Moldova* OR Moldavia* OR Moldavian OR Russia OR Russian OR Siberia OR Tajikistan* OR Tadjikistan* OR Tadzhik* OR Tadzhikistan* OR Turkmenistan* OR Turkmen* OR Turkmenia OR Turkmenian OR Ukraine* OR Ukraina OR Ukrainian OR Uzbekistan* OR Uzbek* OR Bangladesh* OR "east Pakistan" OR Bhutan* OR Brunei* OR Cambodia* OR Khmer OR Kampuchea* OR kampudja* OR China OR Manchuria* OR PRC OR Beijing OR Chinese OR Sinkiang* OR "Inner Mongolia" OR Fiji* OR Indian* OR India OR Indonesia OR "East Indies" OR "West Irian" OR Indonesian OR ("New Guinea" AND West ) OR ( rian Jaya Timor) OR Java OR Bali OR Sumatra OR Celebes OR Sulawesi OR (Malay Archipelago ) OR Madoera OR Madura OR Kiribati OR Micronesia* OR "Gilbert Islands" OR "Mariana Islands" OR "Marshall Islands" OR Nauru OR "Pacific Islands" OR Tuvalu OR "Ellice Islands" OR "Caroline Islands" OR Micronesia* OR Lao OR LAOS* OR Malaysia* OR Malaya* OR Malay* OR Sabah OR Sarawak OR "North Borneo" OR Maldives OR Pemba OR Cocos OR "Johnston Island" OR "Gilbert Islands" OR "Mariana Islands" OR Nauru OR Tuvalu OR "Caroline Islands" OR Micronesia OR Mongolia* OR Mongolian OR Myanmar* OR Myanma* OR Burma OR "Pleasant Island" OR Nepal* OR Palau* OR Belau OR Palaos OR Pelew OR Papua OR ("New Guinea" East ) OR PNG OR Philippines OR Phillipines OR Samoa* OR Samoan OR "Navigator Island" OR "Navigator Islands" OR "Solomon Islands" OR "Norfolk Island" OR "Sri Lanka" OR Ceylon OR Thailand* OR Siam OR Timor OR Tonga* OR Tuvalu OR "Ellice Islands" OR Vanuatu OR "New Hebrides" OR Vietnam* OR " Viet Nam" OR Albania* OR Bosnia* OR Bulgaria* OR Bulgarian OR Bulgarians OR Croatia* OR Hungary* OR Kosovo* OR Montenegro* OR "North Macedonia" OR Macedonia* OR Yugoslavia OR Poland* OR Romania* OR Rumania* OR Roumania* OR Serbia OR Turkey* OR Barbuda* OR Antigua* OR Argentina* OR Argentine* OR Aruba* OR bahama* OR Bahamas* OR Barbados* OR Belize* OR "British Honduras" OR Bolivia* OR Brazil* OR Chile* OR chili* OR Colombia* OR "Costa Rica" OR Dominica* OR Dominican OR Ecuador* OR "Galapagos Islands" OR Salvador* OR Grenada* OR Guatemala* OR Guyana* OR Guiana OR Haiti* OR Hayti* OR Honduras* OR Jamaica* OR Mexico OR Mexican OR Nicaragua* OR Panama* OR Paraguay* OR Peru* OR Nevis OR Lucia OR Vincent OR Grenadines OR Surinam* OR Suriname* OR "Dutch Guiana" OR "Netherlands Guiana" OR Trinidad OR Tobago* OR Uruguay* OR Venezuela* OR Afghanistan* OR Algeria* OR Bahrain* OR "French Somaliland" OR "afar and issa land" OR "french sudan" OR Egypt* OR "united arab republic" OR Iran* OR Persia* OR Iraq* OR Jordan* OR transjordan OR Kuwait* OR Lebanon* OR Lebanese OR libia* OR Libya* OR Mauritania* OR Morocco* OR Ifni OR Oman* OR Muscat* OR Pakistan* OR Qatar* OR Katar* OR Quatar* OR (Saudi* Arabia) OR Somalia* OR Sudan* OR Syria* OR Syrian OR Tunisia* OR tunesia* OR UAE OR Emirates OR "Trucial States" OR "Abu Dhabi" OR Yemen* OR Sanaa OR Aden OR Angola* OR Benin* OR Dahomey* OR Botswana* OR Bechuanaland OR Kalahari OR (Burkina Faso) OR "Upper Volta" OR Burundi* OR Urundi OR (Cabo Verde) OR "Cape Verde" OR Cameroon* OR Cameroons* OR Cameron* OR cameroun* OR Ubangi-Shari OR Centrafrican OR Centrafrique OR (Central African ) OR Chad* OR Tchad OR Comoros* OR "comoro islands " OR "Comoro Mayotte" OR Congo* OR "Côte dIvoire" OR "Cote diIvoire" OR " Ivory Coast" OR (Equatorial Guinea*) OR "rio muni " OR "Spanish Guinea" OR "Rio Muni" OR Eritrea* OR Eswatini OR Swaziland* OR Ethiopia* OR Ethiopians OR Gabon* OR Gabonese OR gaboon OR Gambia* OR Ghana* OR "Gold Coast" OR Guinea OR Guinea-Bissau OR "Portuguese Guinea" OR Kenya* OR Lesotho* OR Basutoland OR Liberia* OR Madagascar* OR Madagascan* OR Madagascans* OR Malagasy OR Malawi* OR Nyasaland OR Mali OR Mauritius* OR " Agalega Islands" OR Mozambique* OR (Portuguese "East Africa") OR Namibia* OR "Southwest Africa" OR ( South "West Africa") OR Niger* OR Nigeria* OR Rwanda OR Ruanda* OR "São Tomé and Príncipe " OR Senegal* OR Seychelles* OR Sierra* OR Leone OR Salone OR "South Africa" OR RSA OR "South Sudan" OR Tanzania* OR Zanzibar* OR Tanganyika OR Togo* OR Togolese* OR Uganda* OR Zambia* OR "Northern Rhodesia" OR Zimbabwe* OR Rhodesia)):ti,ab,kw #11 and #12 ((Care OR cares OR management*)):ti,ab,kw MeSH descriptor: [Disease Management] explode all trees #14 or #15 #13 and #16 |
