# Supplementary material for: Challenges in traumatic spinal cord injury care in developing countries – a scoping review
Source: Front Public Health. 2024 Aug 19;12:1377513. doi: 10.3389/fpubh.2024.1377513 (PMC11368135; doi:10.3389/fpubh.2024.1377513)
Supplement: Supplementary file 2 [file Data_Sheet_2.docx]

**Appendix B-Supplementary Tables:** Extracted findings of included studies.

| **Table S1.** Studies characteristics (ordered based on the year of publication) | | | | | | | | | | |  |
| --- | --- | --- | --- | --- | --- | --- | --- | --- | --- | --- | --- |
| **No** | **1^st^ author** | **Year** | **Country** | **Method** | **Sample size** | **Challenges** | | | | **Ref** |  |
|  |  |  |  |  |  | **Injury prevention** | **Prehospital** | **In hospital** | **Post-hospital** |  |  |
| 1 | Iwegbu | 1983 | Nigeria | Cross-sectional | 48 | ✓ | ✓ | ✓ | ✓ | [12] |  |
| 2 | Chacko | 1986 | India | Cross-sectional | 218 |  | ✓ | ✓ | ✓ | [13] |  |
| 3 | Shanmugasundaram | 1988 | India | Interventional | 844 |  |  | ✓ |  | [14] |  |
| 4 | Wang | 1990 | China | Retrospective cohort |  |  | ✓ | ✓ | ✓ | [15] |  |
| 5 | Faure | 1990 | South Africa | Commentary |  |  |  | ✓ |  | [16] |  |
| 6 | Levy | 1998 | Zimbabwe | Retrospective survey | 432 |  |  |  | ✓ | [17] |  |
| 7 | Lugo | 2007 | Colombia | Prospective quasi-experimental before-and-after study | 42 |  |  |  | ✓ | [18] |  |
| 8 | Pandey | 2007 | India | Cross-sectional | 60 |  | ✓ |  |  | [19] |  |
| 9 | Raissi | 2007 | Iran | Commentary | 88 | ✓ |  | ✓ | ✓ | [20] |  |
| 10 | Rasouli | 2007 | Iran | Retrospective cohort | 64 | ✓ |  |  |  | [6] |  |
| 11 | Nwadinigwe | 2008 | Nigeria | Retrospective cohort | 22 | ✓ |  |  |  | [21] |  |
| 12 | Rathore | 2008 | Pakistan | Commentary |  | ✓ | ✓ | ✓ | ✓ | [22] | |
| 13 | Hadir | 2011 | Haiti | Interventional article |  |  | ✓ | ✓ | ✓ | [23] |  |
| 14 | Babamohamadi | 2011 | Iran | Semi-structured interviews | 18 |  |  |  | ✓ | [24] |  |
| 15 | Scovil | 2012 | Nepal | Prospective cohort | 37 |  |  |  | ✓ | [25] |  |
| 16 | Singh | 2012 | India | Review |  |  | ✓ | ✓ | ✓ | [26] |  |
| 17 | Razzak | 2013 | Bangladesh | Cross-sectional | 56 |  | ✓ | ✓ |  | [27] |  |
| 18 | Shah | 2013 | Nepal | Commentary |  |  |  |  | ✓ | [28] |  |
| 19 | Oderud | 2014 | Zimbabwe | Semi-structured interviews | 23 |  |  |  | ✓ | [29] |  |
| 20 | Shrestha | 2014 | Nepal | Commentary |  | ✓ | ✓ | ✓ | ✓ | [30] |  |
| 21 | Lofvenmark | 2015 | Botswana | Cross-sectional | 49 | ✓ | ✓ | ✓ |  | [87] |  |
| 22 | Rathore | 2015 | Pakistan | Commentary |  |  |  |  | ✓ | [33] |  |
| 23 | Al-Chalabi | 2015 | UAE | Retrospective cohort | 232 |  |  | ✓ | ✓ | [34] |  |
| 24 | Debebe | 2016 | Ethiopia | Cross-sectional | 84 | ✓ | ✓ | ✓ |  | [35] |  |
| 25 | Lofvenmark | 2016 | Botswana | Semi-structured interviews | 13 |  |  |  | ✓ | [31] |  |
| 26 | Lofvenmark | 2017 | Botswana | Prospective cohort | 39 |  |  | ✓ | ✓ | [32] |  |
| 27 | Choi | 2017 | Cambodia | Cross-sectional | 80 |  |  | ✓ | ✓ | [36] |  |
| 28 | Moshi | 2017 | North-East  Tanzania | Retrospective cohort | 288 |  |  | ✓ | ✓ | [37] |  |
| 29 | Munakomi | 2017 | Nepal | Prospective cohort | 163 | ✓ | ✓ | ✓ | ✓ | [38] |  |
| 30 | Suman | 2017 | India | Interventional | 840 |  |  |  | ✓ | [39] |  |
| 31 | Chhabra | 2018 | India | Cross-sectional (Online survey) | 160 (60 patients, 66 consumers, and 34 experts) |  | ✓ | ✓ | ✓ | [86] |  |
| 32 | AlSaleh | 2018 | Saudi Arabia | Cross-sectional | 50 |  |  |  | ✓ | [63] |  |
| 33 | Mansoor | 2018 | Pakistan | cross sectional | 34 |  |  |  | ✓ | [77] |  |
| 34 | Foongchomcheay | 2018 | Thailand | Cross-sectional semi-structured interviews | 11 | ✓ |  |  | ✓ | [82] |  |
| 35 | Alwashmi | 2019 | Saudi Arabia | cross-sectional | 121 |  |  |  | ✓ | [40] |  |
| 36 | Ghajarzadeh | 2019 | Iran | Retrospective cohort | 830 |  | ✓ |  |  | [42] |  |
| 37 | Rahman | 2019 | Malaysia | Qualitative phenomenology design | 5 |  |  |  | ✓ | [43] |  |
| 38 | Yusuf | 2019 | Nigeria | Retrospective review | 133 |  | ✓ | ✓ |  | [44] |  |
| 39 | Hussain | 2019 | Bangladesh | descriptive/Cross-sectional | 260 |  |  |  | ✓ | [89] |  |
| 40 | Leidinger | 2019 | Tanzania | Retrospective cohort | 180 |  | ✓ | ✓ |  | [73] |  |
| 41 | PaulusMokgachane | 2019 | Botswana | cross-sectional | 57 |  |  |  | ✓ | [80] |  |
| 42 | Shabany | 2019 | Iran | semi-structured interviews | 43 |  |  |  | ✓ | [83] |  |
| 43 | Visser | 2019 | Cape Town, South Africa | cross-sectional survey | 71 |  |  |  | ✓ | [84] |  |
| 44 | Dorjbal | 2019 | Mongolia | semi-structured interviews | 12 |  |  |  | ✓ | [68] |  |
| 45 | Azadmanjir | 2020 | Iran | retrospective focus group discussions and semi-structured interviews | - | ✓ |  |  |  | [70] |  |
| 46 | Dorjbal | 2020 | Mongolia | Qualitative study | 16 |  |  |  | ✓ | [41] |  |
| 47 | Nade | 2020 | Tanzania | Cross-sectional pilot study | 48 |  |  | ✓ | ✓ | [46] |  |
| 48 | Alve | 2020 | Bangladesh | Qualitative study (Ethnographic)/ Cross-sectional | 19 |  |  |  | ✓ | [48] |  |
| 49 | Jakimovska | 2020 | Macedonia | Prospective cohort study | 38 | ✓ |  | ✓ | ✓ | [45] |  |
| 50 | Mahooti | 2020 | Iran | Qualitative study (semi-structured interview) | 16 |  |  |  | ✓ | [49] |  |
| 51 | Al-Otaibi | 2021 | Saudi Arabia | Cross-sectional | 4,914 |  | ✓ |  |  | [50] |  |
| 52 | Lessing | 2020 | Tanzania | Retrospective cohort | 270 |  |  | ✓ |  | [74] |  |
| 53 | Liu | 2020 | Bangladesh | community-based intervention (Randomized controlled trial) | 204 |  |  |  | ✓ | [75] |  |
| 54 | Magogo | 2020 | Tanzania | Retrospective case series | 97 |  | ✓ | ✓ |  | [76] |  |
| 55 | Farahani | 2021 | Iran | Qualitative study (semi-structured interview) | 17 |  |  |  | ✓ | [52] |  |
| 56 | Jha | 2021 | India | Prospective | 57 |  | ✓ | ✓ |  | [53] |  |
| 57 | Munakomi | 2021 | Nepal | Cross-sectional | 71 |  |  |  | ✓ | [54] |  |
| 58 | Odunaiya | 2021 | Nigeria | Cross-sectional and explorative qualitative | 34 |  |  | ✓ | ✓ | [55] |  |
| 59 | Toluse | 2021 | Nigeria | Retrospective observational | 99 | ✓ |  | ✓ |  | [56] |  |
| 60 | Zuckerman | 2021 | Tanzania | Retrospective Cohort | 101 |  | ✓ | ✓ |  | [57] |  |
| 61 | Badenhorst | 2021 | South Africa | Qualitative | 31 |  |  |  | ✓ | [66] |  |
| 62 | Höfers | 2021 | Seven countries: Sweden, China, USA, Russia,  Israel, The State of Palestine, and Norway | International multi-center cross-sectional (Online survey) | Pediatric |  |  |  | ✓ | [67] |  |
| 63 | Kumprou | 2021 | Thailand | cross-sectional | 163 |  |  |  | ✓ | [71] |  |
| 64 | Kuzu | 2021 | Turkey | Cross-sectional survey | 82 |  |  |  | ✓ | [72] |  |
| 65 | Oliveira | 2021 | Brazil | Retrospective cohort | 154 |  |  |  | ✓ | [78] |  |
| 66 | Pilusa | 2021 | Gauteng, South Africa | Semi-structured interviews | 44 |  |  |  | ✓ | [95] |  |
| 67 | Pilusa | 2021 | South Africa | Semi-structured interviews | 17 |  |  |  | ✓ | [93] |  |
| 68 | Yang | 2021 | China | Cross-sectional | 1,355 |  |  |  | ✓ | [85] |  |
| 69 | Baniya | 2022 | Nepal | Cross-sectional | 115 |  | ✓ |  | ✓ | [47] |  |
| 70 | Sharma | 2022 | India | phenomenological qualitative research | 21 |  | ✓ | ✓ | ✓ | [88] |  |
| 71 | Shah | 2022 | Nepal | Retrospective | 48 |  | ✓ | ✓ |  | [58] |  |
| 72 | Smith | 2022 | East Africa vs. North America | Comparative | 67 |  |  | ✓ | ✓ | [59] |  |
| 73 | Uddin | 2022 | Low / Low-to-Middle Income countries (LIC/LMIC) | Mixed-method (small group discussions and online literature search). | 3 small groups |  |  |  | ✓ | [90] |  |
| 74 | Tharu | 2022 | Bangladesh | Cross-sectional | 127 |  |  |  |  | [64] |  |
| 75 | Pilusa | 2022 | South Africa | Qualitative (Focus group discussion) | Four focus groups |  |  |  | ✓ | [91] |  |
| 76 | Ashipala | 2022 | Namibia | Qualitative (Exploratory design) | 15 |  |  |  | ✓ | [65] |  |
| 77 | Shrestha | 2022 | Nepal | structured one-on-one interview | 211 |  | ✓ |  |  | [69] |  |
| 78 | Gowinnage | 2022 | Sri Lanka | cross-sectional | 159 |  |  |  | ✓ | [92] |  |
| 79 | Mohammadi | 2022 | Iran | semi-structured interviews | 25 |  |  |  | ✓ | [81] |  |
| 80 | Pilusa | 2022 | Gauteng, South Africa | semi-structured interviews | 38 |  |  |  | ✓ | [94] |  |
| 81 | Williams | 2023 | South Africa | Qualitative study | 13 |  |  |  | ✓ | [60] |  |
| 82 | Sertkaya | 2023 | Turkey | Cross-sectional | 77 |  |  |  | ✓ | [61] |  |
| **Abbreviations:** UAE: United Arab Emirates, LIC: Low Income Countries, LMIC: Lower Middle-income Countries, USA: United states of America | | | | | | | | | | |  |

| **Table S2.** Injury prevention TSCI challenges in developing countries (ordered based on the year of publication) | | | | | | | | |
| --- | --- | --- | --- | --- | --- | --- | --- | --- |
| **1^st^ author** | **Year** | **Country** | **Challenges** | | | | | **Ref** |
|  |  |  | **Research** | **Strategic legislation** | **Culture** | **Infra-structure** | |  |
| Iwegbu | 1983 | Nigeria |  |  |  | Bad roads and poor standards of driving | | [12] |
| Raissi | 2007 | Iran | Lack of sufficient data |  |  |  | | [20] |
| Rasouli | 2007 | Iran |  |  | Not using restraints and helmets  High-risk driving behaviors |  |  | [6] |
| Rathore | 2008 | Pakistan | Lack of an SCI Registry |  |  |  |  | [22] |
| Shrestha | 2014 | Nepal |  |  |  | High-risk environment at the workplace | | [30] |
| Lofvenmark | 2015 | Botswana |  | No legislation for using seatbelts in the back seats |  | 1. Single Lane highways with no separation from street lights or domestic animal 2. Poor cars and tires bursting due to hot climate | | [87] |
| Debebe | 2016 | Ethiopia |  | Inadequate traffic regulations |  | Non-standard old motor vehicles and poor road traffic infrastructure | | [35] |
| Munakomi | 2017 | Nepal |  |  |  | Inaccessibility to all-weather roads | | [38] |
| Foongchomcheay | 2018 | Thailand | Lack of research on epidemiology,  prevalence and incidence of SCI |  |  |  | | [82] |
| Jakimovska | 2019 | Macedonia |  |  | Diving into shallow water. | 1. High-risk environment at the workplace  2. The poor condition of roads and old motor vehicles | | [45] |
| Azadmanjir | 2019 | Iran | Insufficient SCI registry poor communication between principal and regional investigators, insufficient education to the registrar and a neurosurgeon for AOspine classification |  |  | Insufficient infrastructure for the SCI registry like lack of funds and human resources, lack of technical infrastructure | | [70] |
| Toluse | 2021 | Nigeria |  |  | Not using helmets or seatbelts |  | | [56] |
| Jha | 2021 | India |  |  | Alcohol consumption |  | | [53] |
| **Abbreviation:** SCI: Spinal Cord Injury, AO classification: Arbeitsgemeinschaft für Osteosynthesefragen classification | | | | | | | | |

| **Table S3.** Pre-hospital TSCI challenges in developing countries (ordered based on the year of publication) | | | | | | | | |
| --- | --- | --- | --- | --- | --- | --- | --- | --- |
| **1^st^ author** | **Year** | **Country** | **Challenges** | | | | | **Ref** |
|  |  |  | **Public Education** | **Integrated neurotrauma care system** | **Equipment** | **Transportation** | **Adherence to guidelines** |  |
| Iwegbu | 1983 | Nigeria |  |  |  | Transportation by untrained personnel |  | [12] |
| Chacko | 1986 | India |  | Lack of sufficient SCI centers |  | Non-ideal transport from the injury site to the hospital |  | [13] |
| Wang | 1990 | China |  |  | EMC with no special knowledge, skills, and equipment for SCI patients | Far distance between the accident site and EMC |  | [15] |
| Pandey | 2007 | India | Lack of knowledge about essential precautions for transportation |  |  |  |  | [19] |
| Nwadinigwe | 2008 | Nigeria |  |  |  | Initial transportation to primary care centers causing delays in transport to SCI centers |  | [21] |
| Rathore | 2008 | Pakistan |  |  |  | 1. Inadequate airlift capabilities 2. Using all types of vehicles for transporting patients 3. Healthcare providers’ unawareness of log rolling (using boards) and immobilization techniques | Unawareness of the ASIA system for differentiating complete and incomplete SCI | [22] |
| Bourgeosis Hadir | 2011 | Haiti | lack of skilled human resources | Lack of a referral system | lack of emergency medical care and equipment | lack of limited mobility during transportation |  | [23] |
| Singh | 2012 | India | lack of basic knowledge about the immobilization |  |  | lack of basic knowledge about transportation |  | [26] |
| Razzak | 2013 | Bangladesh | Poor public knowledge about immobilization | Poor National Health Referral System |  | 1. transfer by untrained people and non-standard vehicle 2. Delay in transport |  | [27] |
| Shrestha | 2014 | Nepal |  |  |  | Lack of immobilization during transportation. |  | [30] |
| Lofvenmark | 2015 | Botswana |  | Poor referral system |  | 1. transfer by non-medical staff and non-standard vehicles without proper immobilization.  in a sitting position with non-medical staff) |  | [87] |
| Debebe | 2016 | Ethiopia |  |  |  | Lack of immobilization during transportation |  | [35] |
| Munakomi | 2017 | Nepal |  |  | Lack of ambulances due to bureaucracy in importing ambulances from international borders | 1. Delay in transportation 2. Public behavior to emergency vehicle |  | [38] |
| Chhabra | 2017 | India | Transportation by untrained people | Lack of sufficient referral system |  | 1. Lack of trained staff  2. Delay in transporting to SCI centers | Poor first aid response  Poor pre-hospital care. | [86] |
| Ghajarzadeh | 2019 | Iran |  |  |  | Unequipped vehicle transportations |  | [42] |
| Yusuf | 2019 | Nigeria |  |  |  | Delay in transportation |  | [44] |
| Leidinger | 2019 | Tanzania |  | 1. Poor prehospital management  2. lack of referral hospitals |  | Delay in transportation |  | [73] |
| Al-Otaibi | 2020 | Saudi Arabia | Lack of public awareness about the main signs of cervical TSCI, proper first aid response, and immobilization. |  |  |  |  | [50] |
| Magogo | 2020 | Tanzania |  |  |  | Delay in transportation |  | [76] |
| Zuckerman | 2021 | Tanzania |  |  |  | Delay in admission |  | [57] |
| Jha | 2021 | India |  |  |  | Transfer by an untrained staff.  Neck immobilization |  | [53] |
| Baniya | 2022 | Nepal | Lack of knowledge about the importance of pharmacological agents for SCI-associated depression |  |  |  |  | [47] |
| Sharma | 2022 | India | Lack of awareness and basic knowledge about SCI. | Poor National Health Referral System |  |  |  | [88] |
| Shah | 2022 | Nepal |  |  |  | Poor referrals and transport caused delayed transfer of patients which is associated with higher cardiopulmonary complications. |  | [58] |
| Smith | 2022 | East Africa vs. North America |  |  |  | The patients are not immobilized properly during transportation in East Africa, in contrast with North America. |  | [59] |
| Shrestha | 2022 | Nepal | 1. Lack of trained human resources  2. Lack of knowledge about essential precautions for transportation | Poor national referral transfers | 1. Not providing essential equipment like oxygen cylinders, spine boards, and cervical collars during transport and no spine stabilization  2. Lack of ambulance  availability | 1. delay in hospital arrival and reaching a tertiary trauma center  2. 3. Lack of trained health care Staff |  | [69] |
| **Abbreviation:** EMS: Emergency Medical Services, ASIA: American Spinal Injury Association, SCI: Spinal Cord Injury | | | | | | | | |

| **Table S4.** In-hospital TSCI challenges in developing countries (ordered based on the year of publication) | | | | | | | |
| --- | --- | --- | --- | --- | --- | --- | --- |
| **1^st^ author** | **Year** | **Country** | **Challenges** | | | | **Ref** |
|  |  |  | **Medical staff** | **Hospital system and equipment** | **Failure to work with patients and their peers** | **Indirect and Environmental Problems** |  |
| Iwegbu | 1983 | Nigeria |  | Lack of equipment and facilities like beds and catheters |  |  | [12] |
| Chacko | 1986 | India | Inadequate nursing care available | Lack of sufficient SCI centers |  |  | [13] |
| Shanmugasundaram | 1988 | India |  | Underdeveloped methods of care for SCI patients in a General Hospital |  |  | [14] |
| Wang | 1990 | China | Lack of knowledge skills and manpower | Early hospital discharge | UTI and bedsores |  | [15] |
| Faure | 1990 | South Africa | Increased workload | Lack of facilities |  | Lack of fund | [16] |
| Raissi | 2007 | Iran | Lack of man force | Inappropriate setting of priorities of care | Lack of familiarity of temporary personnel with cultural aspects to effectively communicate with the patients |  | [20] |
| Rathore | 2008 | Pakistan | Few qualified neurosurgeons and spinal surgeons | 1. Lack of spinal units 2. Using charpoys (traditional beds which are unsuitable for SCI patients) 3. Mobilization after spinal fixation without spinal braces |  | Patients’ reluctancy to be discharged due to fear of loss of financial support | [22] |
| Bourgeosis Hadir | 2011 | Haiti | Lack of locally trained staff |  |  |  | [23] |
| Singh | 2012 | India | lack of trained staff in initial management | lack of specialized centers | numerous decubitus ulcer and UTIs |  | [26] |
| Razzak | 2013 | Bangladesh | No provision for injury management | Intermediate admission |  |  | [27] |
| Shrestha | 2014 | Nepal | 1. Lack of trained personnel  2. Inadequate training | 1. Absence of standard treatment protocols  2. Lack of a well-equipped center  3. Less than ideal environment for surgical treatment |  | Economic constraints | [30] |
| Lofvenmark | 2015 | Botswana | Lack of neurosurgeons | 1. Poor access to ICU 2. Lack of theatre space |  |  | [87] |
| Al-Chalabi | 2015 | UAE |  | Lack of SCI centers |  |  | [34] |
| Debebe | 2016 | Ethiopia | Lack of staff and resources | 1. Patient leaving without being admitted | Long stay at the adult emergency center without receiving adequate care | 1. Not having insurance 2. Overcrowding in EC | [35] |
| Lofvenmark | 2017 | Botswana |  | Basic care lagging in general wards |  |  | [32] |
| Choi | 2017 | Cambodia |  | 1. Limited availability of pedicle screw sets  2. Very firm beds |  |  | [36] |
| Moshi | 2017 | North-East  Tanzania |  | Insufficient emergency and intensive care services |  |  | [37] |
| Munakomi | 2017 | Nepal | Lack of manpower and health facilities outside the capital city |  |  | Financial constraints | [38] |
| Chhabra | 2018 | India |  | 1. Insufficient acute management.  2.Late admission  3. patients stated they reached a specific institution after being moved between two or more institutions. | 1. Late admission  2. Lack of awareness about SCI  3. Sending patients home after acute management  4. Neglected traumatic SCI | 1.Financial constraints 2.Not reaching to SCI center | [86] |
| Yusuf | 2019 | Nigeria |  | 1.Lack of Theatre space | Delay in radiological imaging  Delay in surgical intervention | 1.Financial constraints.  2.Absence of health insurance coverage | [44] |
| Jakimovska | 2019 | Macedonia | Lung physiotherapy is not performed for SCI patients due to the lack of staff. |  | Pressure ulcer  Long hospital stays |  | [45] |
| Leidinger | 2019 | Tanzania | Lack of education and expertise on Spinal injury in local health centers | 1.Limited availability of equipment (implants, invasive blood pressure monitoring)  2. Under-resourced and ill-equipped regional hospitals | 1.Delay in operation  2. No protocol existed to aid surgeons in early surgical, ICU admission, and hemodynamic monitoring priority.  3. No well-defined decision-making guidelines in electing imaging studies in SCI patients | 1.Low insurance coverage  2. High prices of surgical implants  3. Delay in discharge due to problems like financial constraints | [73] |
| Nade | 2020 | Tanzania |  |  |  | Unaffordability of CIC materials Low healthcare insurance rate | [46] |
| Lessing | 2020 | Tanzania |  | Delay in referral from regional hospitals to tertiary hospitals |  | Financial constraints that prevent SCI patients from undergoing surgery | [74] |
| Magogo | 2020 | Tanzania |  | Unavailability of implants |  | 1.Delay to surgery due to financial constraints  2. High cost of implants | [76] |
| Jha | 2021 | India |  | Admission to public hospitals was a predictor of major complications in TSCI. |  |  | [53] |
| Odunaiya | 2021 | Nigeria |  | 1.Poor structural facilities and operational protocols.  2. No place informal caregivers’ stay. | 1.Inappropriate attitudes of health workers  2. Lack of communication between physicians and informal caregivers |  | [55] |
| Toluse | 2021 | Nigeria |  |  | Pressure ulcers and UTI |  | [56] |
| Zuckerman | 2021 | Tanzania |  | 1. Neglecting surgery indication due to insufficient funds or equipment like surgical tools, operating beds, or spinal implants.  2. Lack of appropriate ICU services. | Delay in decompressive surgery | Insufficient funds | [57] |
| Sharma | 2022 | India | Healthcare providers generally lack sufficient awareness and knowledge about SCI. | Premature discharge from acute care | Inadequacies of patient and informal caregiver education |  | [88] |
| Shah | 2022 | Nepal |  |  | SCI neglected complications |  | [58] |
| Smith | 2022 | East Africa vs. North America |  | Misdiagnosis and underestimating the injury due to unavailability of imaging modalities like MRI for everyone. | Spine injuries scoring systems are not simple, reliable and comprehensive. |  | [59] |
| **Abbreviations:** CIC: Clean intermittent catheterization, SCI: Spinal Cord Injuries, TSCI: Traumatic Spinal Cord Injuries EC: Emergency Centers, UTI, Urinary Tract Infections, ICU: Intensive Care Unit, MRI: Magnetic Resonance Imaging | | | | | | | |

| **Table S5.** Post-hospital TSCI challenges in developing countries (ordered based on the year of publication) | | | | | | | |
| --- | --- | --- | --- | --- | --- | --- | --- |
| **1^st^ author** | **Year** | **Country** | **Challenges** | | | | **Ref** |
|  |  |  | **Facilities** | **Education, employment, and rehabilitation** | **Follow up** | **Patients** |  |
| Iwegbu | 1983 | Nigeria | Equipment lacks including catheters and wheelchairs |  | Lack of follow-up in treatment of complications |  | [12] |
| Chacko | 1986 | India | Inadequate rescue and retrieval systems | Difficulties in bladder function management due to educational, socio-economical and environment problems |  |  | [13] |
| Wang | 1990 | China |  | No rehabilitation or extended care |  | Low rate of coming back to the community | [15] |
| Levy | 1998 | Zimbabwe | Inadequate housings | 1. Financial problems  2. No prospect employment 3. Minimal social welfare assistance |  |  | [17] |
| Lug | 2007 | Colombia |  | Lack of a low-cost, out-patient programs which can increase functional level and facilitate the social integration of people suffering from spinal cord injury (not based on other communities' programs). |  |  | [18] |
| Raissi | 2007 | Iran | 1. Lack of facilities for social activities. 2. Ethical issues in allocating facilities | 1. Transportation problems for rehabilitation and lack of mobile teams 2. Misinformation about SCI rehabilitation 3. Lack of educational equipment 4. Lack of birth consultation and education 5. No education about heat weather effect on SCI | Unskilled NGOs’ working with disabled patients | 1. Issue of cultural belief 2. Dependency on family | [20] |
| Rathore | 2008 | Pakistan |  | 1. Lack of rehabilitation specialists and centers 2. Non-existent educational materials in local language 3. Inability to return to work |  |  | [22] |
| Bourgeosis hadir | 2011 | Haiti |  |  | Poor follow up |  | [23] |
| Babamohamadi | 2011 | Iran | Lack of facilities and necessary equipment and inaccessible buildings | 1. Lack of knowledge and poor education 2. Lack of employment opportunities 3. Financial limits for welfare organization for providing rehabilitation  4. Lack of financial resources |  | stigma | [24] |
| Scovil | 2012 | Nepal | 1. Independent access to the community because of the physical terrain.  2. Community participation challenge  3. Inaccessibility to toilet and water 4. Inadequate housing and rugged terrain |  |  |  | [25] |
| Singh | 2012 | India | Inadequate rescue and retrieval systems |  |  |  | [26] |
| Shah | 2013 | Nepal |  |  | 1. Lack of human resource for follow up and rehabilitation care 2. Lack of awareness among the medical personnel and general population 3. Exorbitant health care policy |  | [28] |
| Oderud | 2014 | Zimbabwe | Lack of access to school buildings and accessible and affordable transport |  |  | 1. Stigma and attitudes | [29] |
| Shrestha | 2014 | Nepal |  | 1. Inadequate education about surgery side effects 2. Overlooked rehabilitation  3. Few rehabilitation centers (NGOs) | Unawareness and not getting benefit from governmental financial support |  | [30] |
| Rathore | 2015 | Pakistan |  | 1. Neglected and confused rehabilitation. |  | Negative general attitudes of society toward disability | [33] |
| Al-chalabi | 2015 | UAE |  | 1. Not well local rehabilitation team |  | Economic problems | [34] |
| Lofvenmark | 2016 | Botswana | Barriers to accessibility, including the absence of ramps, inaccessible public transportation, and sandy terrain | Unemployment and income problems |  | Hiding disabled family members due to stigma | [31] |
| Lofvenmark | 2017 | Botswana | Lack of catheters | Lack of nursing homes |  | Patients’ reluctance to perform self-catheterization and to have a suprapubic catheter | [32] |
| Choi | 2017 | Cambodia |  | Nonexistent physical rehabilitation |  |  | [36] |
| Moshi | 2017 | North-East  Tanzania | Lack of appropriate wheelchairs |  |  |  | [37] |
| Munakomi | 2017 | Nepal |  | Lack of spine rehabilitation centers | Lack of a peer support for SCI patients and their care providers. |  | [38] |
| Suman | 2017 | India |  | 1. Economic issues 2. Poor guidance and lack of awareness | Distance to travel for follow-up |  | [39] |
| Chhabra | 2018 | India | 1. Lack of assistive technology like wheelchairs  2. transport infrastructure unsuitable for wheelchair users. | 1. Inadequate vocational and physical rehabilitation 2. Financial barriers 3. Accessing problems in the workplace 4. Few sexual and fertility counseling and education and rehabilitation (taboo discussion) 5. Lack of occupational therapy, psychological management, peer counseling, and educational classes 6. The essential principles of rehabilitation, such as goal planning and monitoring, were not adhered to. 7. A multidisciplinary team is needed and many of the specialties are not available. | 1. Lack of appropriate follow-up home-care services 2. A small number of individuals were referred for follow-up due to financial limitations and challenges accessing spinal injury centers.  3. the pre-discharge planning of the follow-up program is neglected.  4. Many comprehensive rehabilitations like occupational, psychological, or sexuality management are not available. | 1. Availability of alternative medicine 2. Community inclusion problems  3. patients are not aware of neurogenic bladder-like urodynamics evaluation.  4. Patients cannot afford single-use catheters.  5. 32% of people are aware of the cleaning or storing technique of reusable catheters.  6. Urinary tract infection  5. 67% of patients and 78% of partners/spouses of TSCI patients did not take sexual counseling.  7. Mobility problem of individuals with SCI in the community or at home | [86] |
| Alsaleh | 2018 | Saudi Arabia | The high cost of urine catheter  difficulty in finding the catheter |  |  | 1.insufficient training about clean intermittent catheterization.  2. Some people experience difficulties in self CIC due to poor function of their upper extremities. | [63] |
| Mansoor | 2018 | Pakistan |  | The practice of reusing disposable catheters is prevalent as a cost-saving measure for bladder management. |  | Clean intermittent catheterization (CIC) is time-consuming and it can impact the family life. | [77] |
| Foongchomcheay | 2018 | Thailand | 1. Commuting problems and physical inaccessibility of the locations of their home, workplace, or recreational facilities and long geographical distances  2. Unsupported public transportation | Unpaid employment |  | 1.Their homes were not adapted to accommodate their disabilities, and their family members discouraged any modifications, insisting that they would take care of everything for them.  2. Stigma  3. Rejection from their family or  Landlord when proposing the idea of environmental adjustment  6. Negative self-advocacy, self-image and self-esteem | [82] |
| Alwashmi | 2019 | Saudi Arabia |  |  |  | Lack of knowledge about vocational rehabilitation | [40] |
| Dorjbal | 2019 | Mongolia | 1.absence of wheelchair-friendly transportation  2.Poor access to the physical environment  3. Inadequate availability of assistive devices and medications. | 1. inadequate health and rehabilitation services  2. Restricted financial means for healthcare  3. Incorrect classification of disabilities in legislation |  | Negative societal attitudes | [41] |
| Rahman | 2019 | Malaysia |  | Rehabilitation programs do not include S=sexual rehabilitation |  | 1. Physical limitations  2. Reduced reproductive ability  3. Financial difficulties impeding sexual performance enhancement medications | [43] |
| Hossain | 2019 | Bangladesh |  | Lack of appropriate income of SCI patients after their disability |  | 1. Pressure ulcer  2. Muscle spasm  3. Problem with social participation.  4. Financial difficulties  5. High incidence of poverty in SCI patients and their families | [89] |
| Alve | 2019 | Bangladesh |  | 1.Inadequate formal support, including rehabilitation professionals and occupational therapists  2. Limited financial sources  3. Lack of educative intervention for the person and the family members about dealing with negative conditions before the discharge | 1. Insufficient provision of long-term follow-up support services in adverse circumstances.  2. Absence of peer support and community-based support systems. | 1.Low self confidence  2. Life became bored and meaningless to SCI patients  3. Reduced social interaction and participation | [9] |
| Dorjbal | 2019 | Mongolia | 1.Lack of equipment and medication  2. Inadequate infrastructures or facilities |  | 1.A lack of understanding and limited knowledge on SCI  2. Lack of rehabilitation policies  3. Insufficient number of trained and qualified personnel |  | [68] |
| paulus-mokgachane | 2019 | Botswana | 1.Structural challenges  (For instance too steep ramps, lack of ramp, sandy or rough terrain outside, narrow doors, a door mat or bathroom)  2. Public services were not effective in meeting the needs of the SCI patients. |  | 1. Insufficient provider  Knowledge about SCI at primary care units  2. Poor accessibility of facilities (location of primary care units was not in line with the patients’ location) | 1. Poor availability of prescribed medication and SCI-related consumables  2. Primary care was not acceptable (ethical, respectful, confidential)  3. Shortage of height-adjustable examining couches at primary care units | [80] |
| Shabany | 2019 | Iran | 1.Lack of assistive device elevators and electric wheelchair  2. Lack of accessibility of entertainment places and the streets and sidewalks | 1.Lack of information about SCI in the patients and the families  2. Loss of work and educational opportunities  3. Lack of training programs  4. Lack of knowledge, poor communication skills, and negative interactions in healthcare providers  5. Poor teamwork in healthcare providers  6. Poor shared-decision making between patients and healthcare providers | Inadequate follow-up services | 1.Negative emotions such as denial, anger, and depression, lack of hope and motivation isolation and poor social communication, change in family structure such as challenges in interpersonal relationships, family roles, and deprivations, loss of comfort, emotional support for the spouse and children, financial burden, physical and psychological harm, and loss of dignity  2. A common desire among families to provide as much help as possible to the injured member result in further dependence and unnecessary disability  3. Lack of SCI awareness in the community  4. High costs of care without insurance coverage  5. Poor insurance coverage  6. Financial constraints  7. Humiliating and pitiful reactions in the society  8. Secondary physical impairments and increased dependence  9. Poor adherence of SCI patients to recommendations for independence | [83] |
| Visser | 2019 | South Africa |  | A lack of knowledge about pressure ulcers in SCI patients |  | 1.Wrong beliefs about pressure ulcers  2.Poor adherence to the recommended guidelines, which included regular pressure relief and daily inspection of their skin  3. Not adequate pressure relief and transfer to prevent pressure ulcers | [84] |
| Jakimovska | 2020 | Macedonia |  | Lack of specialized SCI rehabilitation centers | No SCI related follow up routines |  | [45] |
| Nade | 2020 | Tanzania | Unavailable CIC equipment in local villages | Insufficient data on residual bladder volumes or urodynamic reports due to the unavailability of bladder scans or urodynamic tests.. |  |  | [46] |
| Mahooti | 2020 | Iran | 1. Environment’s barriers (e.g., Streets, passages, and urban furniture)  2. Transportation challenges like the need for taxis that support wheelchair, high cost of transportation | 1. Dealing with employment challenge like insufficient skill-training, inappropriate employment, lack of job support  2. Educational problems like dropouts, changing disciplines, and inability to access educational and training facilities. |  | 1. Denial  2. Isolation, depression and suicidal tendencies.  3. Dependency on others in their daily simple tasks  4. Dealing with a sudden change in the life  5.stigma  6. Discrimination  Lack of proper service delivery system:  1.Insufficiency of service delivery because of comprehensive rules: the policymakers are not aware of SCI patients’ needs.  2. Insufficient specialized training: lack of information on coping with problems and lack of training on performing daily living activities. | [49] |
| Liu | 2020 | Bangladesh | Environment and structural barriers such as steps | Limited  Employment opportunities | Non-negotiable actions typically dictated by healthcare providers | 1.pressure injuries and bladder-related issues  2. Low motivation  3. Social isolation and depression  4. Socioeconomic problems  5. Societal attitudes | [75] |
| Farmahini-farahani | 2021 | Iran |  |  |  | Caregiving at home is associated with many problems to caregiver like lack of knowledge about medical professional care of SCI patients and this demanding duty can affect the caregiver’s wellbeing and cause feeling of insufficiently. Besides they faced lack of support like financial support and insurance, or the support of other relatives. | [52] |
| Munakomi | 2021 | Nepal |  |  | Caregivers and patients must travel a significant distance for their follow-up medical visits in healthcare centers. | The caregivers reported severe burden which make them leave their job permanently and financial issues. The high burden caregiving leads to depression, feeling of loss of identity, anxiety and loss of social and family life. | [54] |
| Odunaiya | 2021 | Nigeria |  |  |  | The informal caregivers deal with a high-level burden of caring the SCI patients and financial difficulties and physical exhaustion. | [55] |
| Badenhorst | 2021 | South Africa | 1.Low quality of care of public hospitals and rehabilitation services.  2. Inaccessible transportation and  3. High costs of transportation  4. High cost of equipment and consumables | 1.Insufficient knowledge about SCI.  2. Lack of knowledge and support about sexual partnership and fertility.  3. Difficulties during arranging appointment with doctors and prolonged waiting periods. |  | 1.Facing difficulties in the communication with health care providers, like negative attitude or lack of autonomy of patients was a barrier in obtaining high quality healthcare.  2. Delay in seeking professional help because of financial difficulties.  3. High rate of out-of-pocket payment | [66] |
| Höfers | 2021 | Seven countries: Sweden, China  USA  Russia  Israel  State of Palestine Norway |  | 1.Deficiency in interdisciplinary and multi professional approach to care in some rehabilitation centers  2. Difficulties with discharge policy in all rehabilitation centers due to home modifications, suitable accommodation, and equipment funding for SCI patients  3. Inadequacy or unavailability of pediatric SCI units in rehabilitation centers |  |  | [67] |
| Kumprou | 2021 | Thailand | Environmental constrains such as uneven surfaces makes it hard to use assistant devices.  Insufficiency of technology without suitable environment |  |  | 1.Budget constraints to afford external devices | [71] |
| Kuzu | 2021 | Turkey |  |  |  | 1.Increased caregiver burden  2. Stigma  3. Low independence in activities  4. Depression | [72] |
| Oliveira | 2021 | Brazil |  | 1.Low rate of return to work  2. Low rate of paid work |  |  | [78] |
| Pilusa | 2021 | South Africa | 1.Shortage of  bowel and bladder management tools  2. Poor quality  Assistive devices  3. High costs of  bowel and bladder management tools  4. Inaccessible built environment such as  public bathrooms improper for wheelchair users, rugged  terrain, and uneven sidewalks  5. The stigma in using public transport system and high cost of private transportation as an alternative | Inadequate empowerment during in-patient rehabilitation. | Lack of knowledge in health care | 1.Lack of medication | [93] |
| Pilusa | 2021 | South Africa | Availability at healthcare facilities, suitability for individual needs, and the cost of purchasing or repairing them, such as wheelchairs |  |  | 1.Secondary health condition (SHC)  2. Mental well-being  3. Lack of knowledge about secondary health conditions  4. Socio-economic issues (lack of adequate finances)  5. Lack of compliance with preventative care practice for SHCs  6. Delayed  Seeking help early | [95] |
| Yang | 2021 | China | 1.Insufficient  accessibility of communication devices and medical supplies  2. Lack of nursing and assistance services  3. Environmental barriers  (Access to homes, public places, and nursing and assistance services, government services ( |  | long- distance traveling for rehabilitation | 1.Environmental barriers (related to climate, financial resources, and negative social attitudes and from acquaintances, colleagues, neighbors, family and friends)  2. Participation  Restrictions due to environmental barriers | [85] |
| Baniya | 2022 | Thailand |  |  | Physical barriers to accessing a healthcare facility |  | [47] |
| Sharma | 2022 | India |  | 1.Rehabilitation lag showing months to years prior to receiving rehabilitation after SCI 2. Inadequacies of rehabilitation |  | 1. Inadequate self-management and occurrence of secondary conditions.  2.Doctor shopping and hopes for miracles.  3.Inadequate expectations for recovery | [88] |
| Williams | 2022 | South Africa |  | The patients are not sufficiently educated about the pain management by the healthcare professionals. | 1.Ineffective pain management due to lack of education in healthcare professionals as well  As peer facilitators  2. Lack of a therapeutic alliance between patients and their healthcare providers and imbalanced decision making | 1. Ineffectiveness of pharmaceutical medications in pain management for a prolonged period.  2. Difficulties in interaction with the clinicians about their pain and their therapeutic treatments, like lack of empathy in healthcare providers and sharing decision making about managing their pain in practice.  3.Feeling of discontentment  4.Feeling unimportant during interactions with healthcare professionals | [60] |
| Tharu | 2022 | Bangladesh |  |  |  | 1.The caregivers’ knowledge about pressure injuries in SCI patients is not sufficient, for example in immobility related problems or positioning or transferring patients.  2. The caregivers’ attitude toward pressure injuries is neutral.  3. The practice of caregivers in the prevention of pressure injuries is not satisfactory.  4. There is a positive correlation between knowledge and practice and knowledge and attitude. | [64] |
| Pilusa | 2022 | South Africa | 1.Limited access to clean water and electricity | 1.Limited number of rehabilitation professionals at primary health centers  2. Lack of rehabilitation guidelines. 3. Educating patients and families when they have not accepted the new condition yet. |  | 1.Educating patients and caregivers about signs of incontinence or pressure injuries. | [91] |
| Ashipala | 2022 | Namibia | Lack of transportation facilities and building standardized accessibility for SCI rehabilitated patients. |  |  | 1.experiencing psychological distress and depression  2. Stigma  3.social discrimination | [65] |
| Samanmalee gowinnage | 2022 | Sri Lanka |  | Lack of knowledge about health sexual dysfunction and reproductive health issues and their management |  | Low confidence in sexual activity | [92] |
| Mohammadi | 2022 | Iran |  | Loss of one’s employment and educational status |  | 1. Emotional distress (crisis-prone behavior, persistent depressive symptoms, rumination, expressions of pity, fear of the future).  2. Diminished sense of dignity (neglect of self-care, sexual dysfunction)  3. Lack of social support  4. Absence of financial companies and sponsors | [81] |
| Pilusa | 2022 | South Africa | Lack of assistive device | 1.lack of rehabilitation facilities  2. Lack of rehabilitation therapists |  | 1.Secondary health condition (pain,  Pressure sores, and UTI)  2. Limited access to healthcare services  3. Lack of home- based care  4. Socioeconomic inequalities in women | [94] |
| Sertkaya | 2023 | Turkey |  | Inadequate health literacy in SCI patients |  |  | [61] |
| Uddin | 2023 | Low / low-to-middle income countries (LIC/LMIC) | 1.Inadequate healthcare resources allocation to SCI patients  2. Emerging technologies are not available in low/ low-to-middle income countries.  3. Lack of facilities that enable the care of patients who lack capacity to consent to maintain their liberty. | 1.Involvement in alternative or unproven therapies to maintain their hope in cure.  2. Insurance system does not cover the cost of long-term rehabilitation.  3. Inability to enroll a long-term indoor rehabilitation program because of financial problems | 1.Women in comparison with men, do not get enough physical, emotional and social care and follow-up after the discharge in LIC/LMIC | 1. Inadequate education and literacy, depression or pain affect the patients’ capacity of decision making and it may be problematic in maintaining ethical issues.  2. The patients desire to end their life in form of euthanasia is controversial and in LIC/LMIC it is affected by the rehabilitation professionals’ religious beliefs and cultural and legal issues | [90] |
| **Abbreviation:** TSCI: Traumatic Spinal Cord Injury, SCI: Spinal Cord Injury, UTI: Urinary Tract Infections, NGO: Non-governmental organization, CIC: Clean intermittent catheterization, SHC: Secondary Health Conditions, LIC: Low Income Countries, LMIC: Lower Middle-income Countries | | | | | | | |

1. Iwegbu, C., *Traumatic paraplegia in Zaria, Nigeria: the case for a centre for injuries of the spine.* Spinal Cord, 1983. **21**(2): p. 81-85.

2. Chacko, V., et al., *Management of spinal cord injury in a general hospital in rural India.* Spinal Cord, 1986. **24**(5): p. 330-335.

3. Shanmugasundaram, T., *The care of SCI patients in the developing nations—Can we stem the rot?* Spinal Cord, 1988. **26**(1): p. 10-11.

4. Wang, D., et al., *China's first total care unit for the spinal cord injured.* Spinal Cord, 1990. **28**(5): p. 318-320.

5. Faure, J., *Spinal cord injury management in 1990.* South African medical journal= Suid-Afrikaanse tydskrif vir geneeskunde, 1990. **78**(8): p. 443-444.

6. Levy, L., et al., *Problems, struggles and some success with spinal cord injury in Zimbabwe.* Spinal Cord, 1998. **36**(3): p. 213-218.

7. Lugo, L.H., F. Salinas, and H.I. García, *Out-patient rehabilitation programme for spinal cord injured patients: Evaluation of the results on motor FIM score.* Disability and Rehabilitation, 2007. **29**(11-12): p. 873-881.

8. Pandey, V., et al., *Care of post-traumatic spinal cord injury patients in India: an analysis.* Indian journal of orthopaedics, 2007. **41**(4): p. 295.

9. Raissi, G.R., *Earthquakes and rehabilitation needs: experiences from Bam, Iran.* The journal of spinal cord medicine, 2007. **30**(4): p. 369-372.

10. Rasouli, M.R., et al., *Comparison of road traffic fatalities and injuries in Iran with other countries.* Chinese Journal of Traumatology (English Edition), 2008. **11**(3): p. 131-134.

11. Nwadinigwe, C. and A. Ugezu, *Management of penetrating spinal cord injuries in a non spinal centre: Experience at Enugu, Nigeria.* Nigerian Journal of Medicine, 2008. **17**(2): p. 205-209.

12. Rathore, F.A., et al., *Spinal cord injury management and rehabilitation: highlights and shortcomings from the 2005 earthquake in Pakistan.* Archives of physical medicine and rehabilitation, 2008. **89**(3): p. 579-585.

13. RC, B.H.A., *Development of a rehabilitation programme after the earthquake in Haiti: opportunities and challenges from emergency to post-acute care.* Physiotherapy 2011. **97**.

14. Babamohamadi, H., R. Negarandeh, and N. Dehghan‐Nayeri, *Barriers to and facilitators of coping with spinal cord injury for Iranian patients: A qualitative study.* Nursing & health sciences, 2011. **13**(2): p. 207-215.

15. Scovil, C.Y., et al., *Follow-up study of spinal cord injured patients after discharge from inpatient rehabilitation in Nepal in 2007.* Spinal Cord, 2012. **50**(3): p. 232-237.

16. Singh, R., *Epidemiology of spinal cord injuries: Indian perspectives.* Epidemiology of Spinal Cord Injuries, 2012: p. 157-68.

17. Razzak, A.T.M.A., *Early care following traumatic spinal cord injury (TSCI) in a rehabilitation centre in Bangladesh-an analysis.* Disability, CBR & Inclusive Development, 2013. **24**(2): p. 64-78.

18. Shah, N., B. Shrestha, and K. Subba, *Spinal cord injury rehabilitation in Nepal.* JNMA J Nepal Med Assoc, 2013. **52**(190): p. 427-31.

19. Oderud, T., *Surviving spinal cord injury in low income countries.* African journal of disability, 2014. **3**(2): p. 1-9.

20. Shrestha, D., *Traumatic spinal cord injury in Nepal.* Kathmandu Univ Med J (KUMJ), 2014. **12**(47): p. 161-2.

21. Löfvenmark, I., et al., *Traumatic spinal cord injury in Botswana: characteristics, aetiology and mortality.* Spinal cord, 2015. **53**(2): p. 150-154.

22. Rathore, F.A., *Revisiting the 2005 earthquake paraplegics: what has changed in a decade?* Journal of Ayub Medical College Abbottabad, 2015. **27**(3): p. 513-514.

23. KM, A.-C., *Spinal cord injuries in UAE: Retrospective, demographic & overview study of patients admitted & managed in Neuro-Spinal Hospital Dubai during last 12 years.* Int J Phys Med Rehabil, 2015. **3**((4)).

24. Debebe, F., et al., *The clinical profile and acute care of patients with traumatic spinal cord injury at a tertiary care emergency centre in Addis Ababa, Ethiopia.* African Journal of Emergency Medicine, 2016. **6**(4): p. 180-184.

25. Löfvenmark, I., et al., *‘The moment I leave my home–there will be massive challenges’: experiences of living with a spinal cord injury in Botswana.* Disability and Rehabilitation, 2016. **38**(15): p. 1483-1492.

26. Löfvenmark, I., et al., *Outcomes after acute traumatic spinal cord injury in Botswana: from admission to discharge.* Spinal Cord, 2017. **55**(2): p. 208-212.

27. Choi, J.-H., et al., *Epidemiology and clinical management of traumatic spine injuries at a major government hospital in Cambodia.* Asian spine journal, 2017. **11**(6): p. 908.

28. Moshi, H., et al., *Traumatic spinal cord injury in the north-east Tanzania–describing incidence, etiology and clinical outcomes retrospectively.* Global health action, 2017. **10**(1): p. 1355604.

29. Munakomi, S., B. Bhattarai, and I. Cherian, *Prospective observational research on the clinical profile and outcome analysis among a cohort of patients sustaining traumatic cervical spine and cord injury in a peripheral tertiary spine care centre in Nepal.* F1000Research, 2017. **6**.

30. D., S., *WHATSAPP AS A TOOL TO IMPROVE DISTANCE UROLOGY CARE AND FOLLOW-UP OF SPINAL CORD INJURY (SCI) PATIENTS IN DEVELOPING COUNTRIES. NEUROUROLOGY AND URODYNAMICS.* WILEY 111 RIVER ST, 2017. **HOBOKEN 07030-5774; NJ USA**.

31. Chhabra, H., S. Sharma, and M. Arora, *Challenges in comprehensive management of spinal cord injury in India and in the Asian Spinal Cord network region: findings of a survey of experts, patients and consumers.* Spinal Cord, 2018. **56**(1): p. 71-77.

32. AlSaleh, A.J., et al., *Long-term compliance with bladder management in patients with spinal cord injury: A Saudi-Arabian perspective.* The Journal of Spinal Cord Medicine, 2020. **43**(3): p. 374-379.

33. Mansoor, S.N. and F.A. Rathore, *Bladder management practices in spinal cord injury patients: A single center experience from a developing country.* J Spinal Cord Med, 2019. **42**(6): p. 786-790.

34. Foongchomcheay, A., et al., *Quality of life after spinal cord injury in Thai individuals: A mixed-methods study.* Hong Kong Physiother J, 2019. **39**(1): p. 35-55.

35. Alwashmi, A.H., *Vocational Rehabilitation Awareness Among Spinal Cord Injury Male Patients in Saudi Arabia: A Brief Communication.* Cureus, 2019. **11**(1).

36. Ghajarzadeh, M. and H. Saberi, *Transportation mode and timing of spinal cord decompression and stabilization in patients with traumatic spinal cord injury in Iran.* Spinal Cord, 2019. **57**(2): p. 150-155.

37. Rahman, P.A., et al., *Sexuality among Men with Spinal Cord Injury.* Indian Journal of Public Health Research & Development, 2019. **10**(1).

38. Yusuf, A.S., et al., *Clinical characteristics and challenges of management of traumatic spinal cord injury in a trauma center of a developing country.* Journal of neurosciences in rural practice, 2019. **10**(03): p. 393-399.

39. Hossain, M.S., et al., *Health status, quality of life and socioeconomic situation of people with spinal cord injuries six years after discharge from a hospital in Bangladesh.* Spinal Cord, 2019. **57**(8): p. 652-661.

40. Leidinger, A., et al., *Spinal trauma in Tanzania: current management and outcomes.* J Neurosurg Spine, 2019. **31**(1): p. 103-111.

41. Paulus-Mokgachane, T.M.M., S.J. Visagie, and G. Mji, *Access to primary care for persons with spinal cord injuries in the greater Gaborone area, Botswana.* Afr J Disabil, 2019. **8**(0): p. 539.

42. Shabany, M., et al., *Family-centered empowerment process in individuals with spinal cord injury living in Iran: a grounded theory study.* Spinal Cord, 2020. **58**(2): p. 174-184.

43. Visser, A.M. and S. Visagie, *Pressure ulcer knowledge, beliefs and practices in a group of South Africans with spinal cord injury.* Spinal Cord Ser Cases, 2019. **5**: p. 83.

44. Dorjbal, D., et al., *Toward an optimization of rehabilitation services for persons with spinal cord injury in Mongolia: the perspective of medical doctors.* Disabil Rehabil, 2021. **43**(15): p. 2200-2212.

45. Azadmanjir, Z., et al., *Sustaining the national spinal cord injury registry of Iran (NSCIR-IR) in a regional center: challenges and solutions.* Iranian journal of public health, 2020. **49**(4): p. 736.

46. Dorjbal, D., et al., *Living with spinal cord injury in Mongolia: A qualitative study on perceived environmental barriers.* The Journal of Spinal Cord Medicine, 2020. **43**(4): p. 518-531.

47. Nade, E.S., et al., *Intermittent catheterisation for individuals with disability related to spinal cord injury in Tanzania.* Spinal Cord Series and Cases, 2020. **6**(1): p. 66.

48. Alve, Y.A., P. Bontje, and S. Begum, *Intra-and interpersonal agency: Resuming occupational participation among persons with spinal cord injury after discharge from in-patient rehabilitation.* Scandinavian Journal of Occupational Therapy, 2020. **27**(1): p. 66-79.

49. Jakimovska, V.M., et al., *Epidemiological characteristics and early complications after spinal cord injury in Former Yugoslav Republic of Macedonia.* Spinal cord, 2020. **58**(1): p. 86-94.

50. Mahooti, F., et al., *Psychosocial challenges of social reintegration for people with spinal cord injury: a qualitative study.* Spinal cord, 2020. **58**(10): p. 1119-1127.

51. Al-Otaibi, M.L., et al., *Levels of public awareness regarding cervical spine injury and the suitable first aid response among adults in Saudi Arabia.* Saudi Medical Journal, 2021. **42**(5): p. 543.

52. Lessing, N.L., et al., *Nonoperative treatment of traumatic spinal injuries in Tanzania: who is not undergoing surgery and why?* Spinal Cord, 2020. **58**(11): p. 1197-1205.

53. Liu, H., et al., *Understanding how a community-based intervention for people with spinal cord injury in Bangladesh was delivered as part of a randomised controlled trial: a process evaluation.* Spinal Cord, 2020. **58**(11): p. 1166-1175.

54. Magogo, J., et al., *Operative Treatment of Traumatic Spinal Injuries in Tanzania: Surgical Management, Neurologic Outcomes, and Time to Surgery.* Global Spine J, 2021. **11**(1): p. 89-98.

55. Farmahini-Farahani, M., et al., *Excruciating Care: Experiences of Care Transition from Hospital to Home among the Family Caregivers of Patients with Spinal Cord Injury.* Nursing and Midwifery Studies, 2021. **10**(1): p. 34-34.

56. Jha, R.K. and R. Gupta, *Traumatic Spinal Cord Injury, an Overview of Epidemiology and Management in Vindhya Region.* Indian Journal of Public Health Research & Development, 2021. **12**(2): p. 304-307.

57. Munakomi, S., et al., *Appraisal of Burden of Caregivers to Chronically Rehabilitated Patients with Spinal Cord Injuries in a Tertiary Neurological Center in Nepal.* Medical and Biomedical Updates, 2021: p. 125-131.

58. Odunaiya, N., et al., *A Mixed-Method Study of Burden of Care and Its Associated Factors Among Informal Caregivers of Individuals with Spinal Cord Injury in Nigeria.* International Journal of Physiotherapy, 2021: p. 121-130.

59. Toluse, A.M. and T.O. Adeyemi, *Epidemiology and clinical outcomes of spinal cord injuries at a level II trauma centre in Nigeria: a longitudinal five year study.* International orthopaedics, 2021. **45**: p. 665-671.

60. Zuckerman, S.L., et al., *Cervical spine trauma in east Africa: presentation, treatment, and mortality.* International Journal of Spine Surgery, 2021. **15**(5): p. 879-889.

61. Badenhorst, M., et al., *Accessing healthcare as a person with a rugby-related spinal cord injury in South Africa: The injured player’s perspective.* Physiotherapy Theory and Practice, 2022. **38**(11): p. 1639-1655.

62. Höfers, W., et al., *Organisation of services and systems of care in paediatric spinal cord injury rehabilitation in seven countries: a survey with a descriptive cross-sectional design.* Spinal Cord, 2022. **60**(4): p. 339-347.

63. Kumprou, M., et al., *External devices among individuals with spinal cord injury from a developing country.* American Journal of Physical Medicine & Rehabilitation, 2021. **100**(10): p. 952-957.

64. Kuzu, D., P.B. Perrin, and M. Pugh Jr, *Spinal cord injury/disorder function, affiliate stigma, and caregiver burden in Turkey.* PM&R, 2021. **13**(12): p. 1376-1384.

65. Oliveira, F.G., et al., *Spinal cord injury and work challenges: an analysis of paid work status and pathways of return to work in Brazil.* Spinal Cord, 2021. **59**(10): p. 1111-1119.

66. Pilusa, S., H. Myezwa, and J. Potterton, *Environmental factors influencing the prevention of secondary health conditions among people with spinal cord injury, South Africa.* PLoS One, 2021. **16**(6): p. e0252280.

67. Pilusa, S., H. Myezwa, and J. Potterton, *‘I forget to do pressure relief’: Personal factors influencing the prevention of secondary health conditions in people with spinal cord injury, South Africa.* South African Journal of Physiotherapy, 2021. **77**(1): p. 1493.

68. Yang, Y., et al., *Environmental barriers and participation restrictions in community-dwelling individuals with spinal cord injury in Jiangsu and Sichuan Provinces of China: Results from a cross-sectional survey.* J Spinal Cord Med, 2023. **46**(2): p. 277-290.

69. Baniya, M., L. Kitrungrote, and J. Damkliang, *Prevalence, severity, and self-management of depressive mood among community-dwelling people with spinal cord injury in Nepal.* Belitung Nursing Journal, 2022. **8**(2): p. 101-107.

70. Sharma, S. and M. Sivakami, *“God will decide her fate”: the trajectories of women with traumatic spinal cord injury in India.* Disability and Rehabilitation, 2023. **45**(12): p. 2003-2012.

71. Shah, G., et al., *Outcome of cervical spine trauma patients admitted to the intensive care unit at a tertiary government referral trauma center in Nepal.* Global spine journal, 2022. **12**(7): p. 1388-1391.

72. Smith, C.J., et al., *A Comparison of Thoracolumbar Injury Classification in Spine Trauma Patients Among Neurosurgeons in East Africa Versus North America.* Cureus, 2022. **14**(11).

73. Uddin, T., et al., *Ethical issues and dilemmas in spinal cord injury rehabilitation in the developing world: a mixed-method study.* Spinal Cord, 2022. **60**(10): p. 882-887.

74. Tharu, N.S., et al., *Caregivers’ knowledge, attitude, and practice towards pressure injuries in spinal cord injury at rehabilitation center in Bangladesh.* Advances in Orthopedics, 2022. **2022**.

75. Pilusa, S.I., H. Myezwa, and J. Potterton, *Services and interventions needed to prevent secondary health conditions throughout the life span of people with spinal cord injury, South Africa.* African Journal of Disability (Online), 2022. **11**: p. 1-8.

76. Ashipala, D.O. and L. Langendorf, *Experiences of spinal cord injury patients admitted to the rehabilitation unit at the national referral hospital in Khomas region, Namibia.* African journal of disability, 2022. **11**: p. 1018.

77. Shrestha, S., K. Shrestha, and C.C. Groves, *Patient handling and transportation from site of injury to tertiary trauma centres in Nepal following acute traumatic spinal cord injury: a descriptive study.* Spinal Cord Ser Cases, 2022. **8**(1): p. 79.

78. Gowinnage, S.S., P. Wicramabahu Senarath Paranayapa, and C. Arambepola, *Sexual and Reproductive Health Experiences, Knowledge and Associations: A Neglected Issue Among Adults with Spinal Cord Injury in Sri Lanka.* Sexuality and Disability, 2022. **40**(4): p. 687-700.

79. Mohammadi, F., et al., *Perception of facing life's challenges in patients with spinal cord injury in Iran: a qualitative study.* BMC Psychol, 2022. **10**(1): p. 202.

80. Pilusa, S., H. Myezwa, and J. Potterton, *Views of health care users and providers: Solutions to improve the prevention of secondary health conditions among people with spinal cord injury, South Africa.* Spinal Cord Ser Cases, 2022. **8**(1): p. 67.

81. Williams, T.L., et al., *Exploration of the Experiences of Persons in the Traumatic Spinal Cord Injury Population about Chronic Pain Management.* Int J Environ Res Public Health, 2022. **20**(1).

82. Sertkaya, Z., et al., *Investigation of health literacy level and its effect on quality of life in patients with spinal cord injury.* The Journal of Spinal Cord Medicine, 2023. **46**(1): p. 62-67.

83. Alve, Y.A. and P. Bontje, *Factors influencing participation in daily activities by persons with spinal cord injury: lessons learned from an international scoping review.* Topics in Spinal Cord Injury Rehabilitation, 2019. **25**(1): p. 41-61.
