# Supplementary material for: Challenges in traumatic spinal cord injury care in developing countries – a scoping review
Source: Front Public Health. 2024 Aug 19;12:1377513. doi: 10.3389/fpubh.2024.1377513 (PMC11368135; doi:10.3389/fpubh.2024.1377513)
Supplement: Supplementary file 3 [file Data_Sheet_3.pdf]

Appendix C-Supplementary Figures: Neurotrauma challenges for each country

Figure S1. Bangladesh

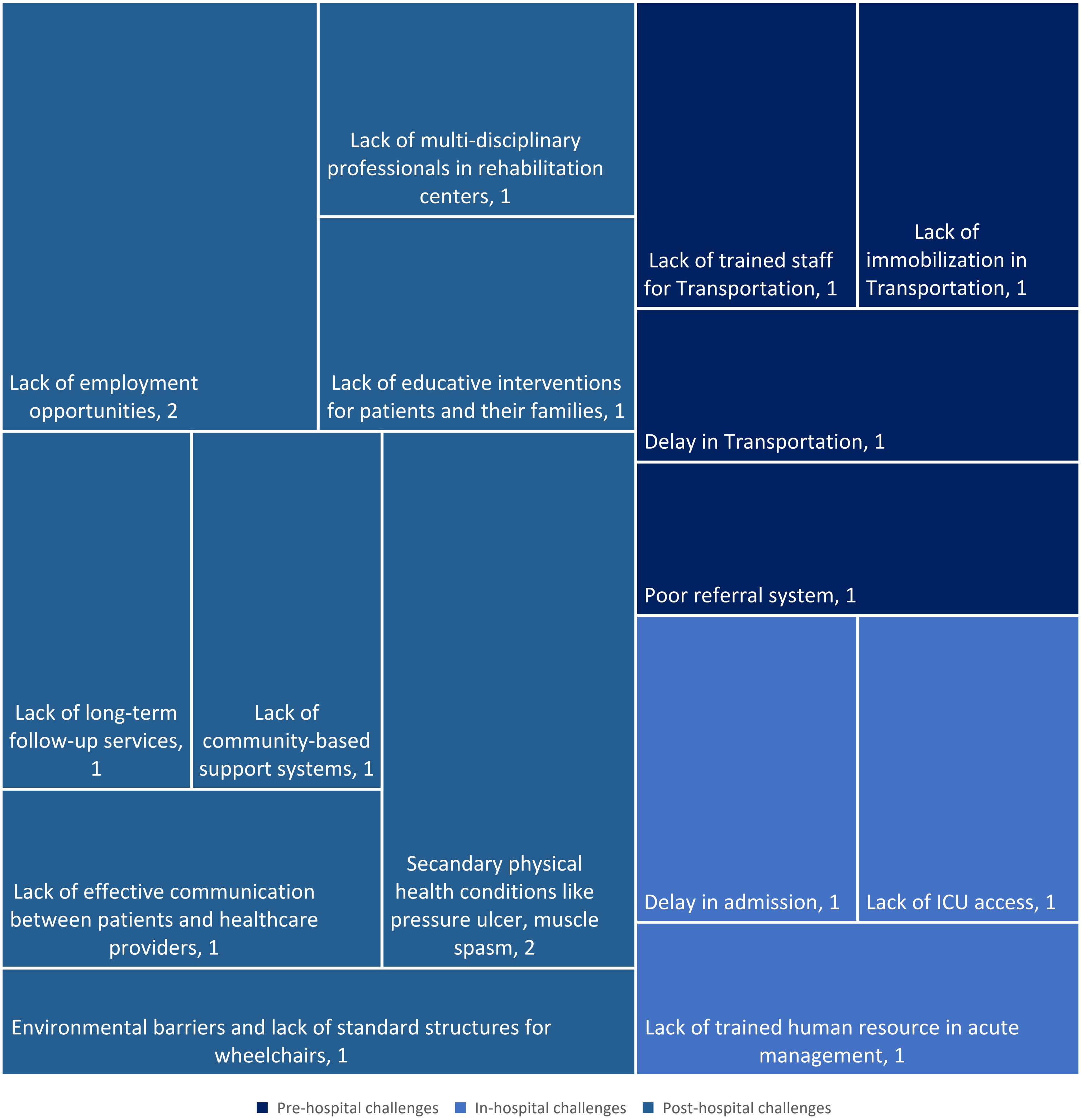

* *The numbers illustrate the number of studies that have reported each problem.*

Figure S2. Botswana

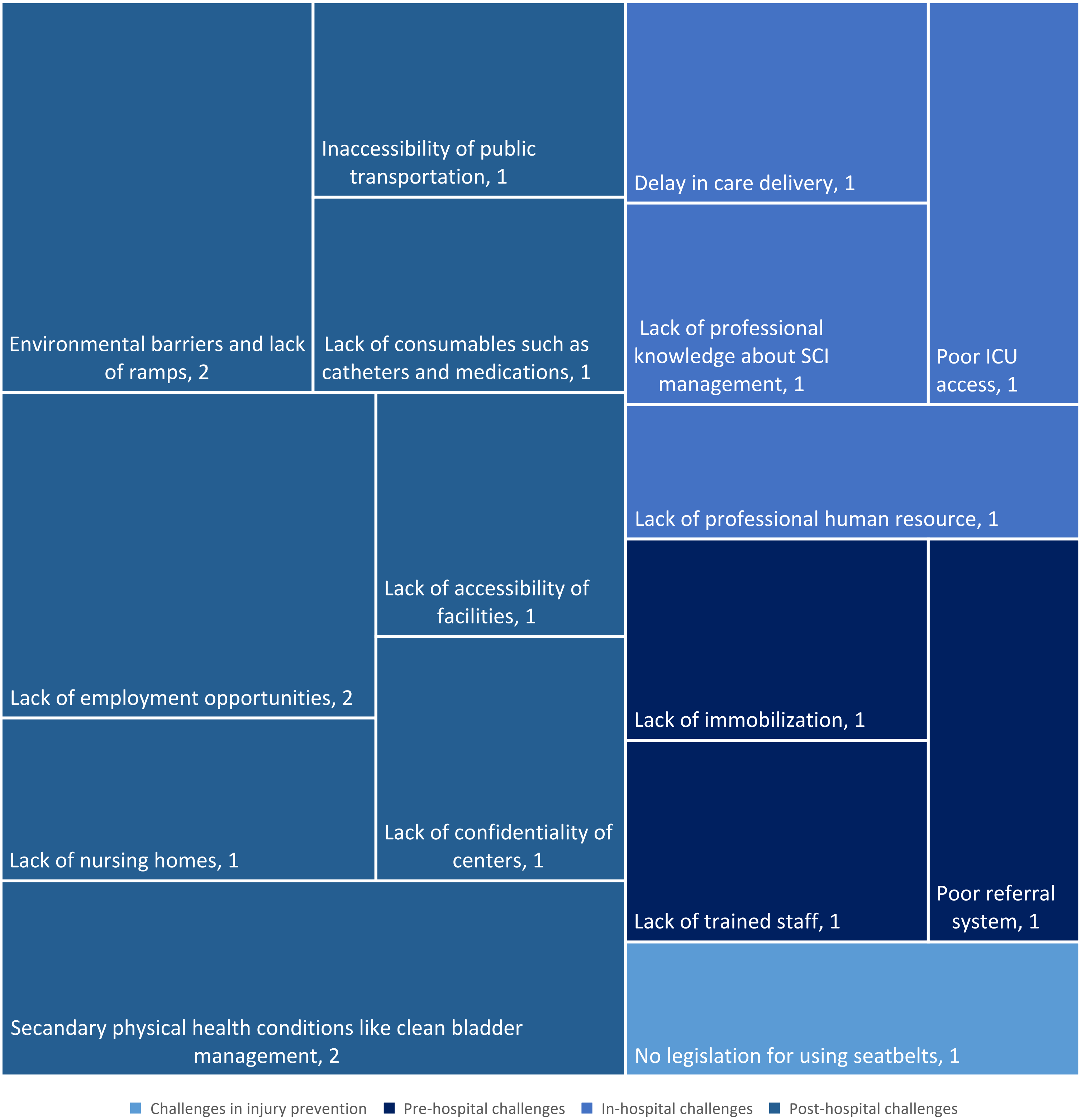

* *The numbers illustrate the number of studies that have reported each problem.*

Figure S3. China

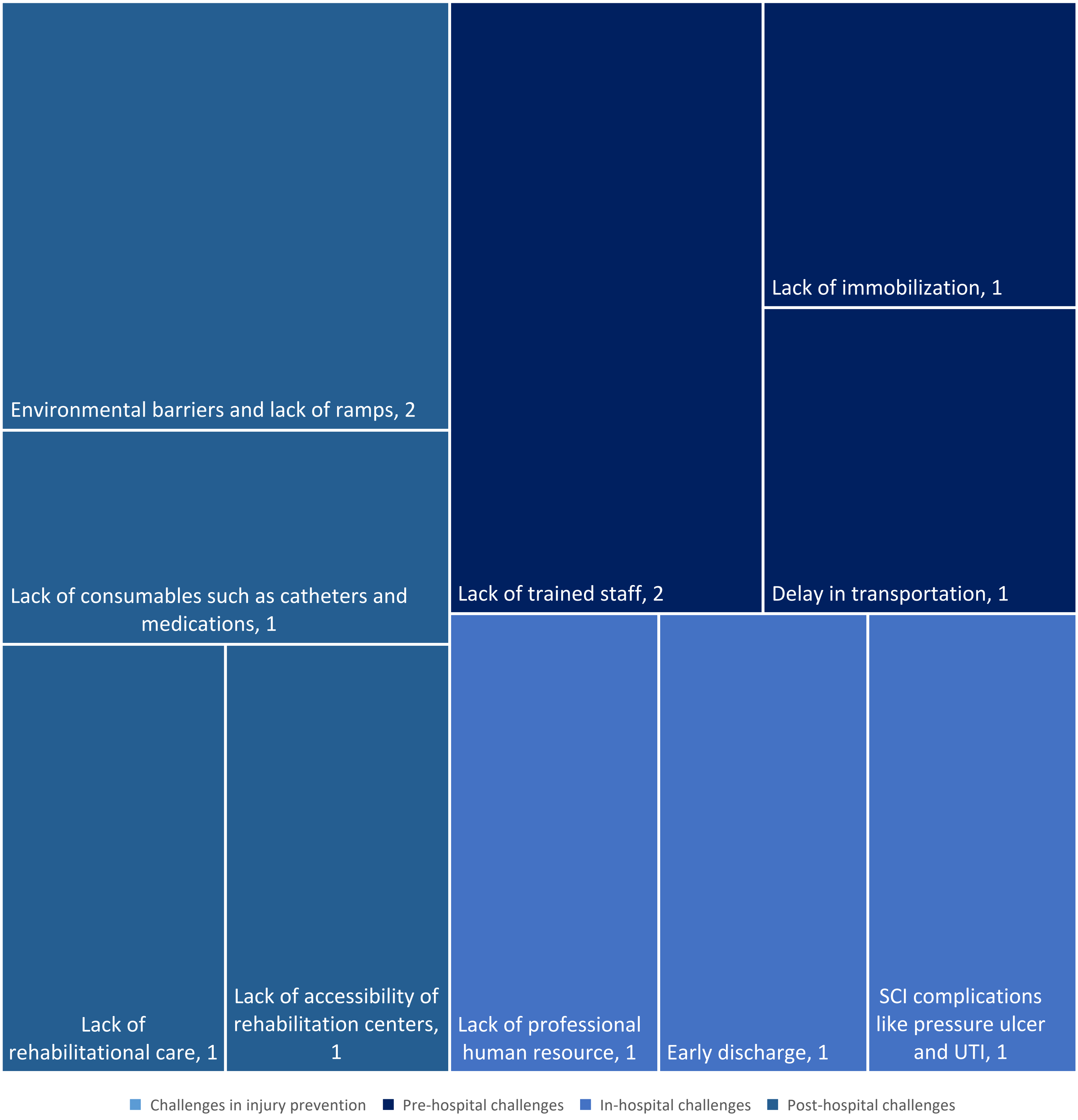

* *The numbers illustrate the number of studies that have reported each problem.*

Figure S4. India

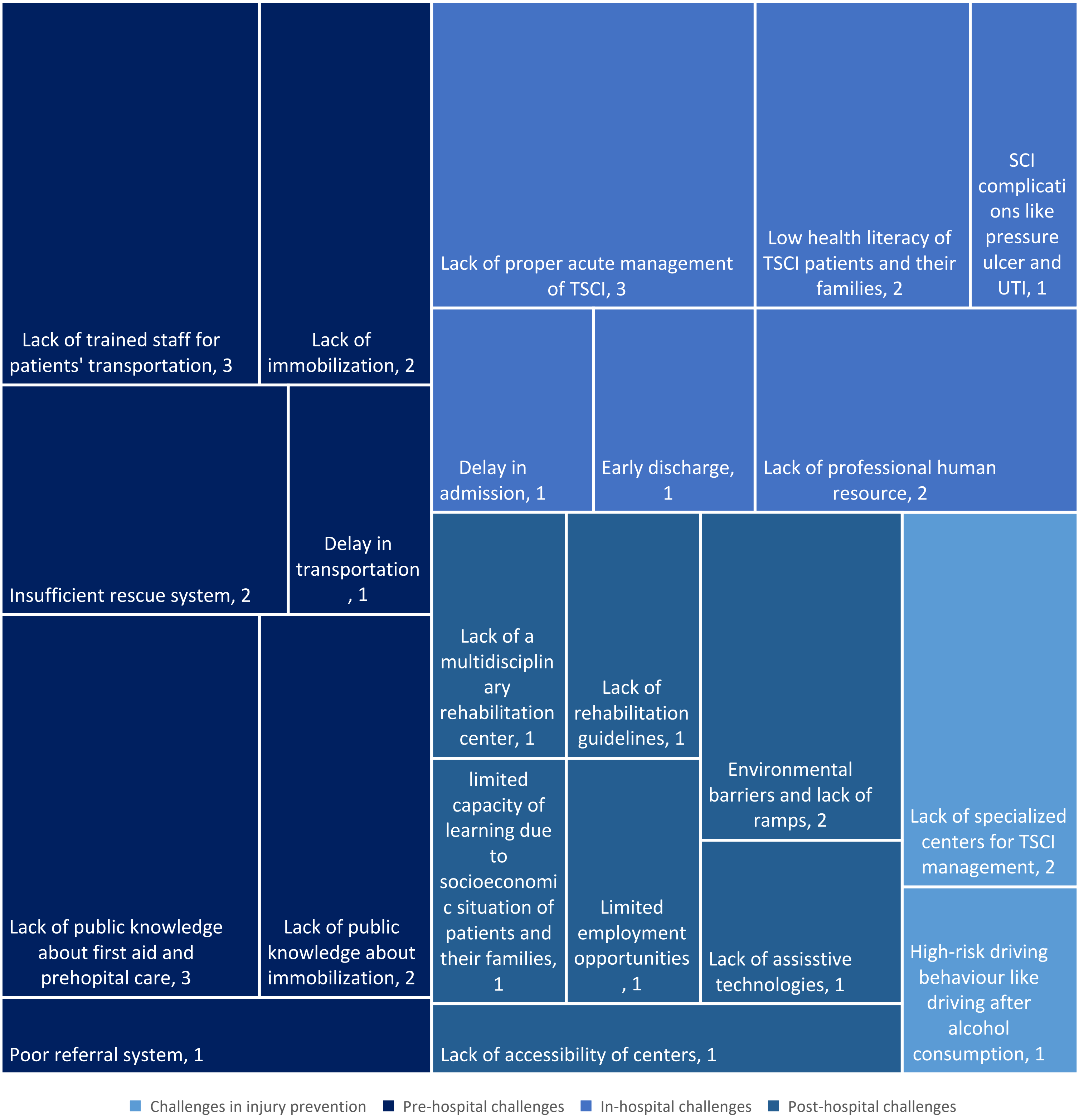

* *The numbers illustrate the number of studies that have reported each problem.*

Figure S5. Iran

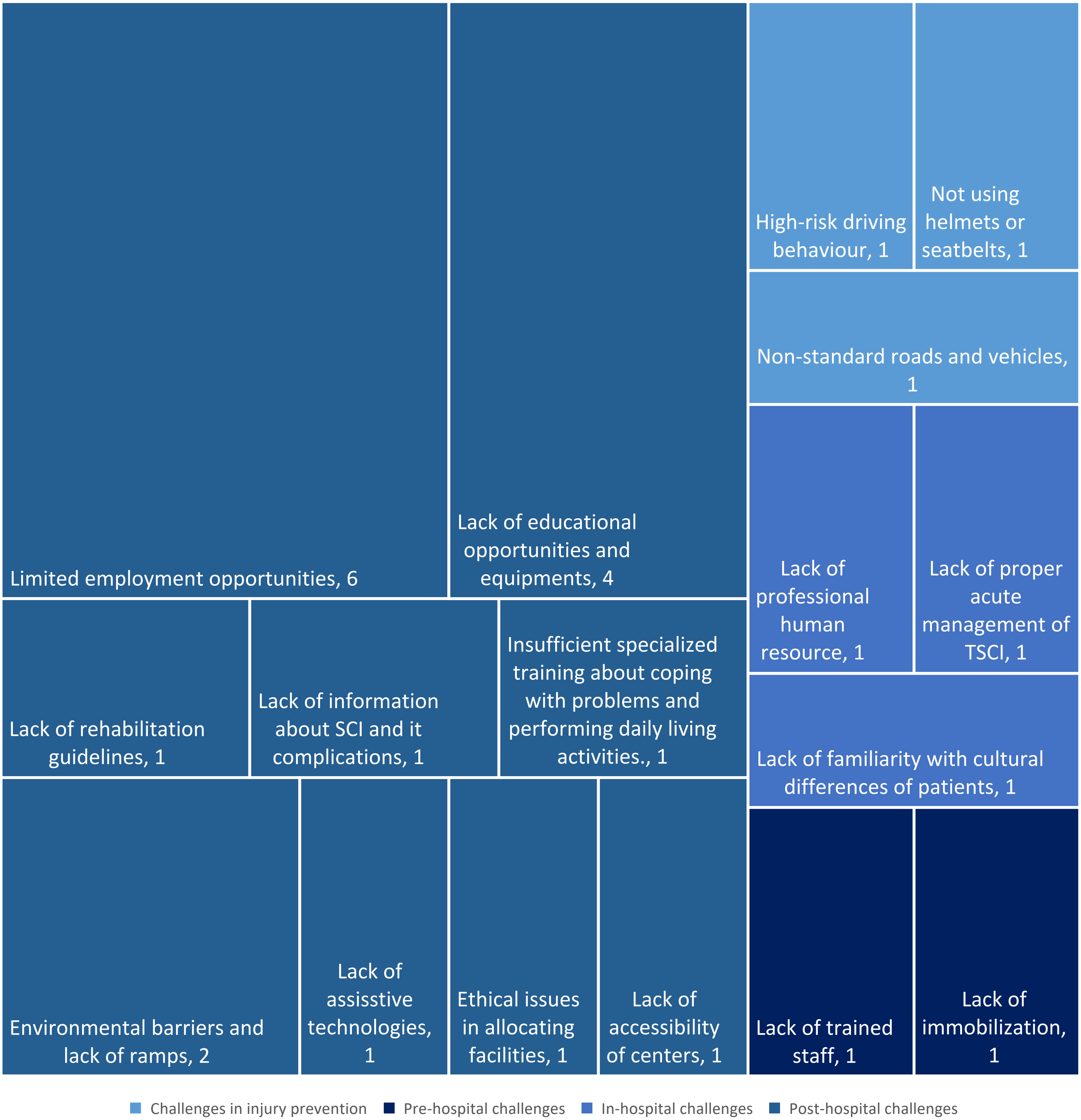

* *The numbers illustrate the number of studies that have reported each problem.*

Figure S6. Mongolia

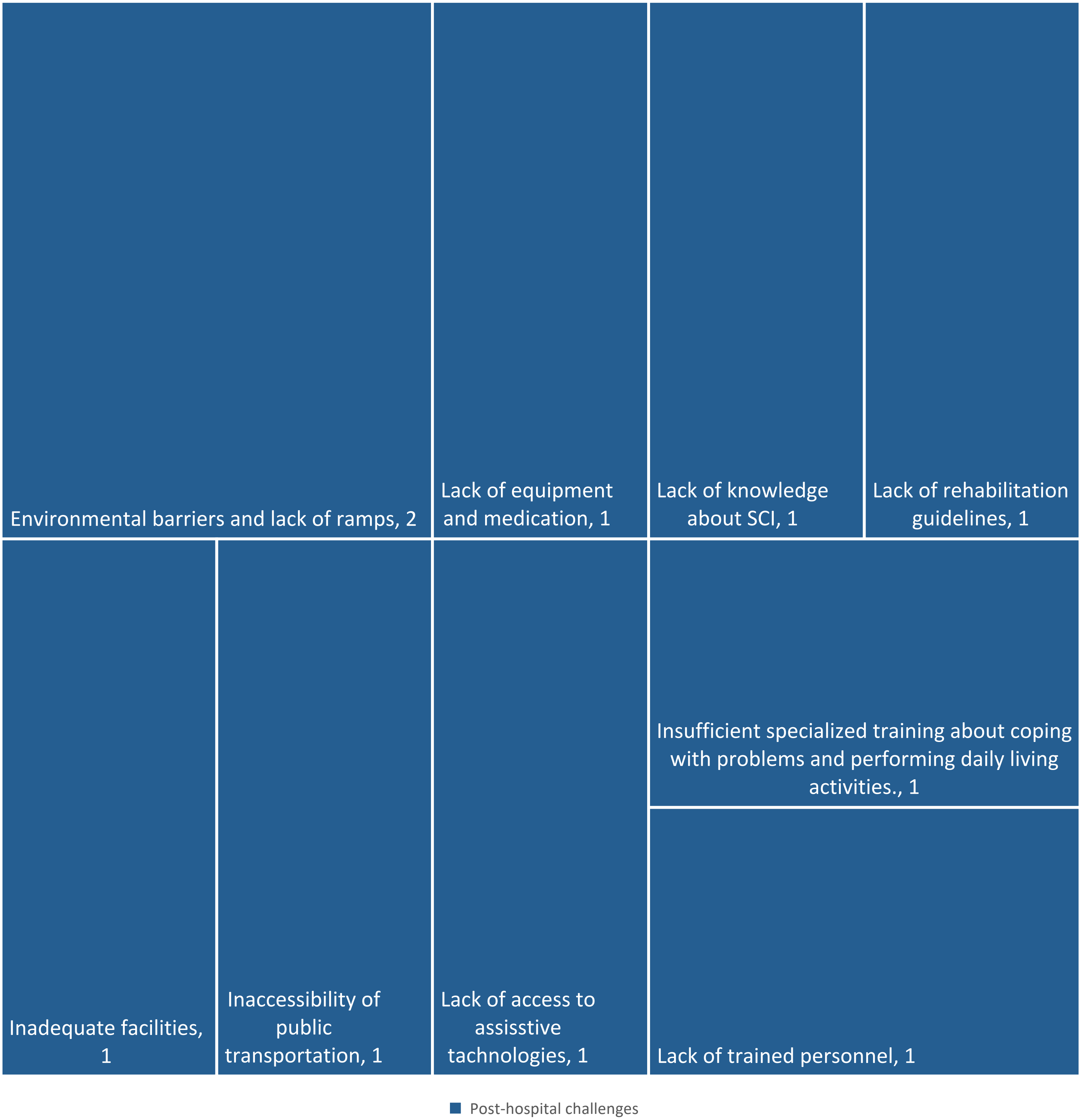

* *The numbers illustrate the number of studies that have reported each problem.*

Figure S7. Nepal

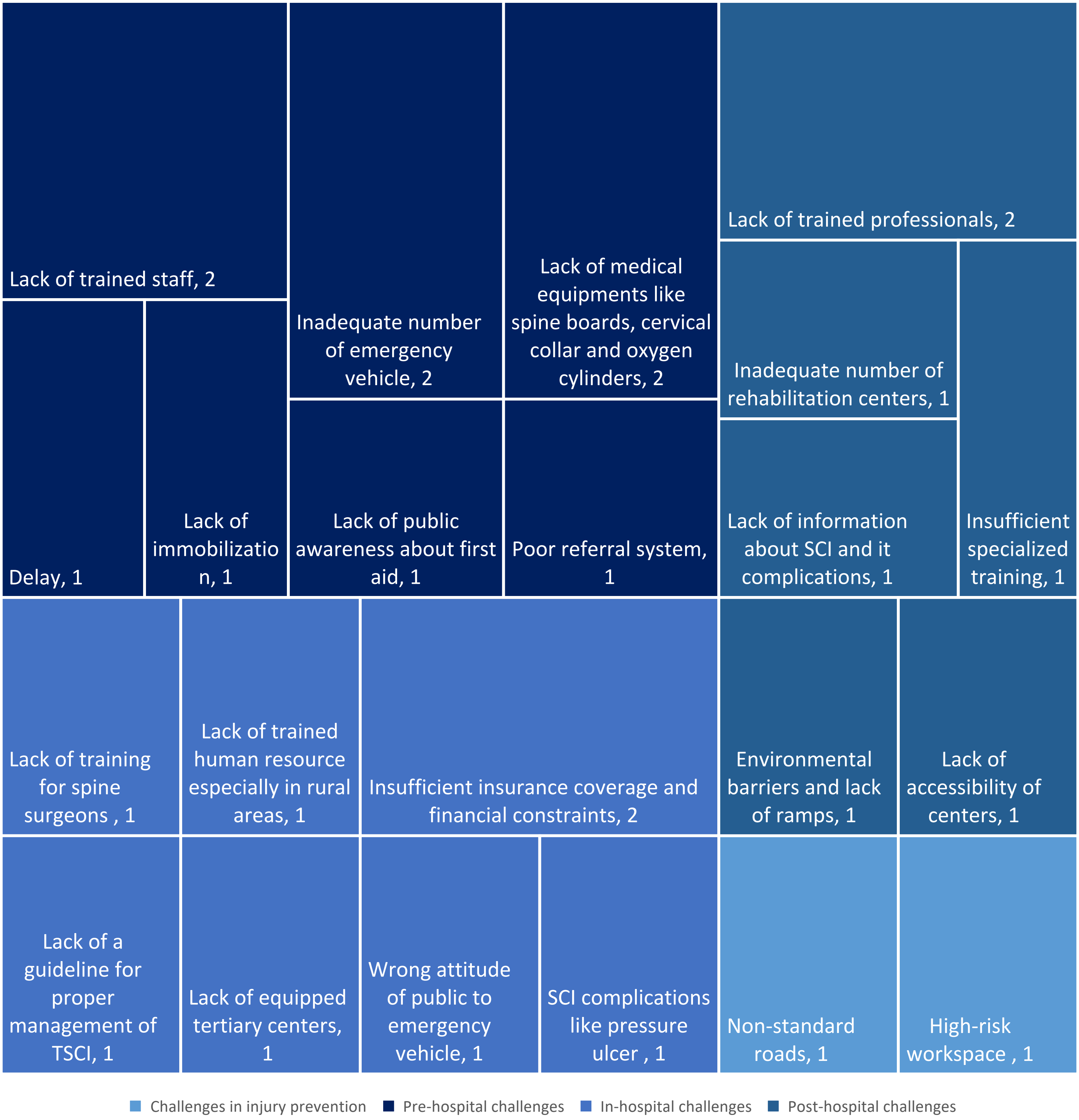

* *The numbers illustrate the number of studies that have reported each problem.*

Figure S8. Nigeria

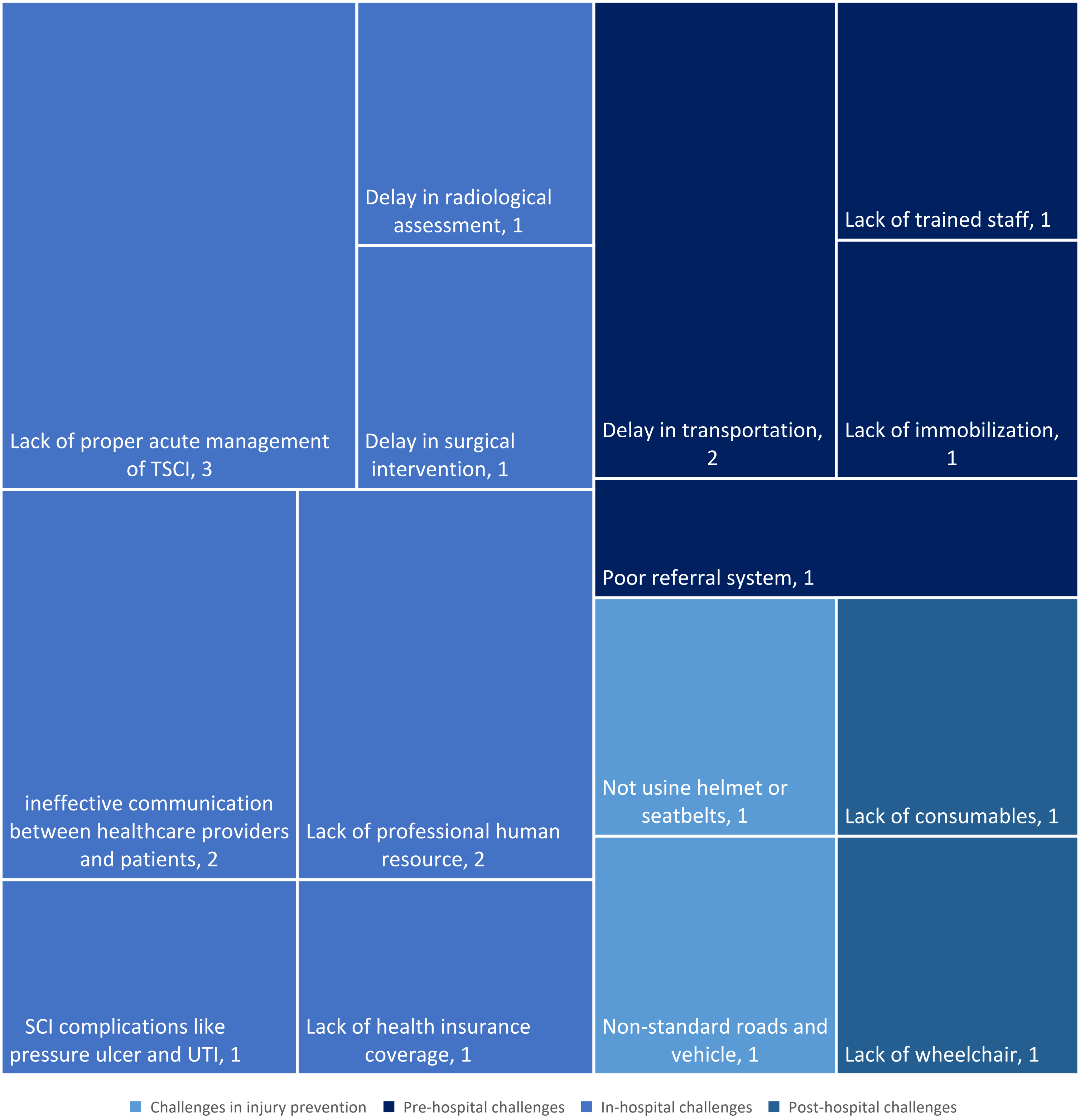

* *The numbers illustrate the number of studies that have reported each problem.*

Figure S9. Pakistan

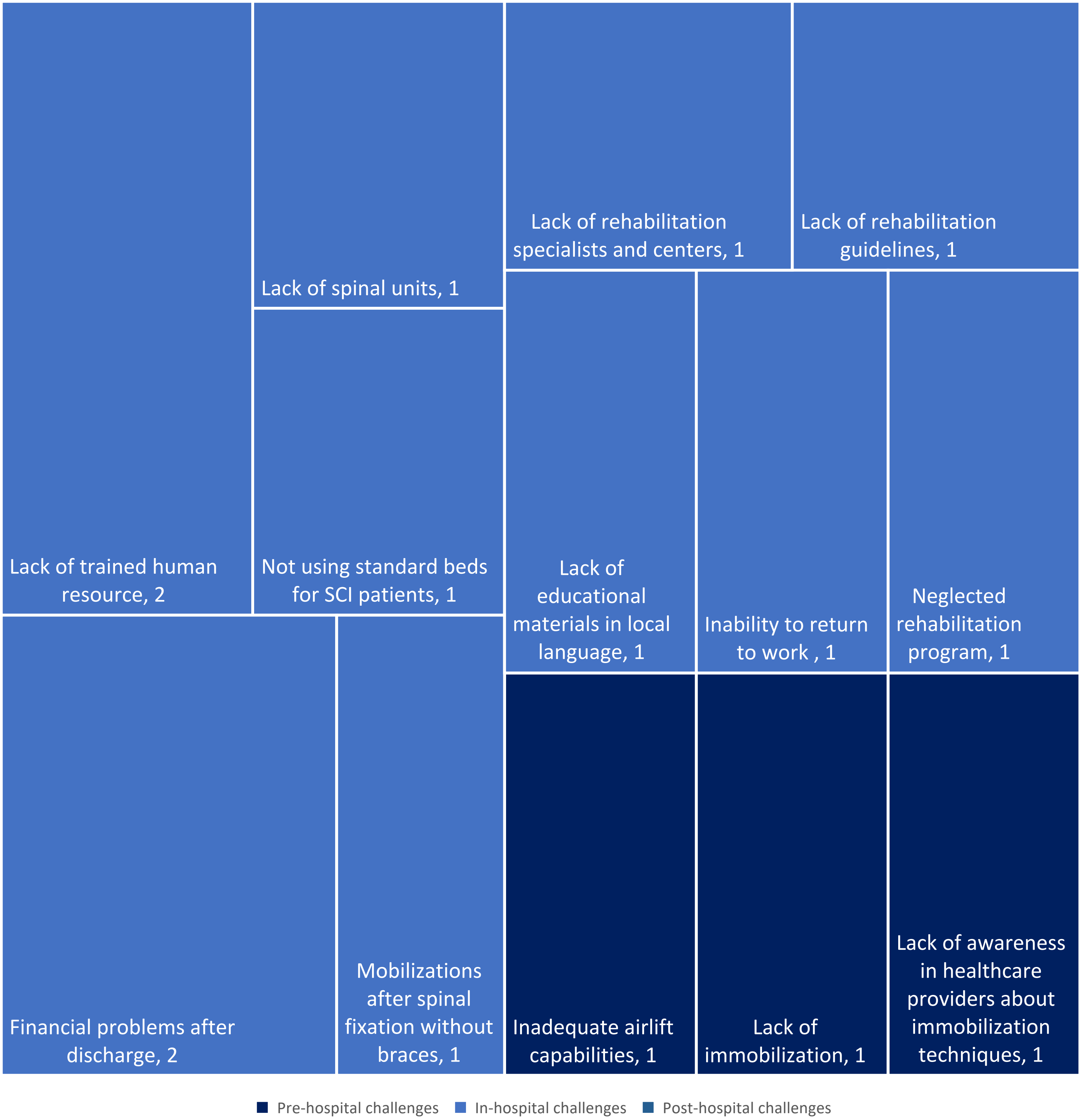

* *The numbers illustrate the number of studies that have reported each problem.*

Figure S10. Saudi Arabia

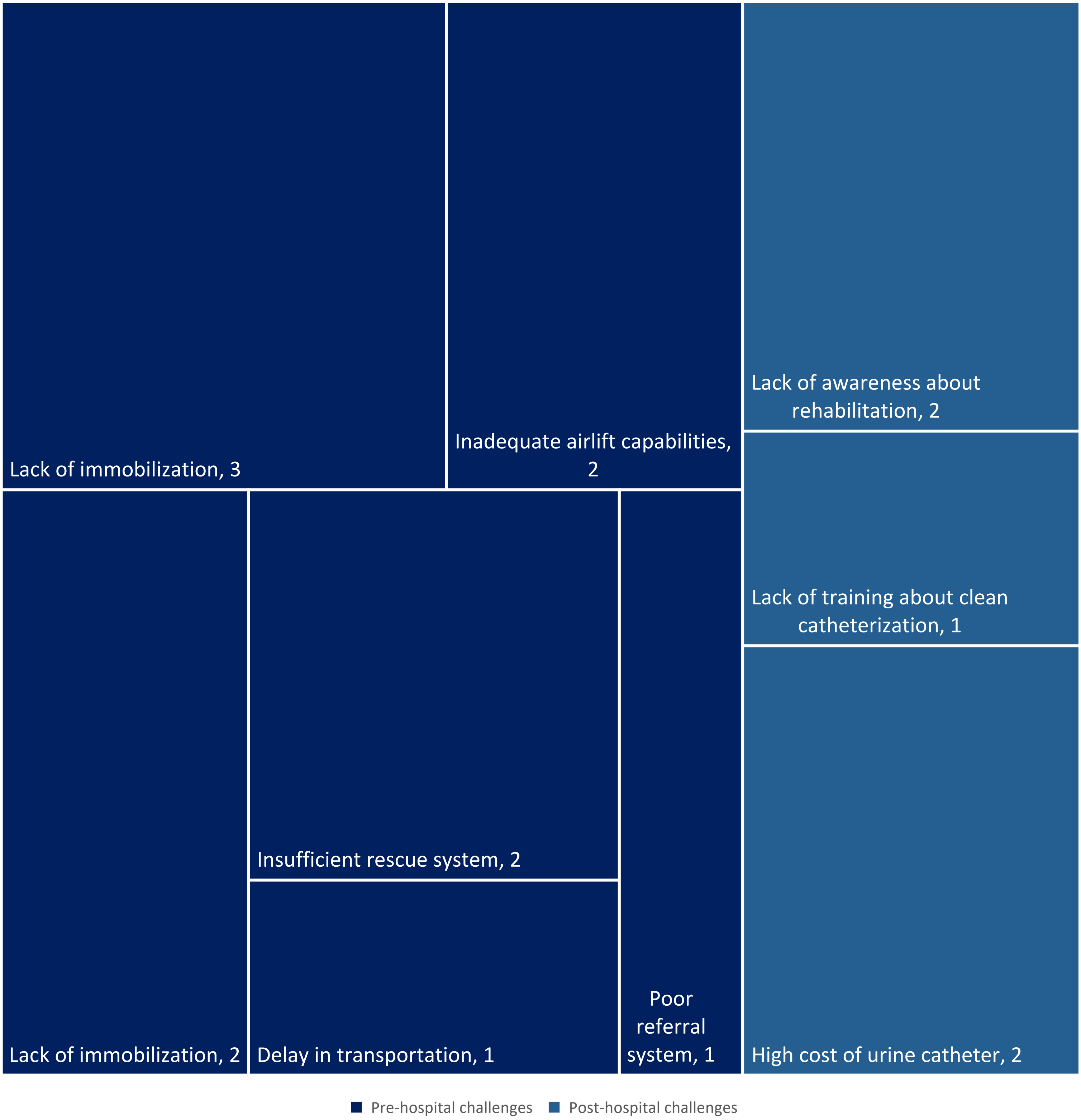

* *The numbers illustrate the number of studies that have reported each problem.*

Figure S11. South Africa

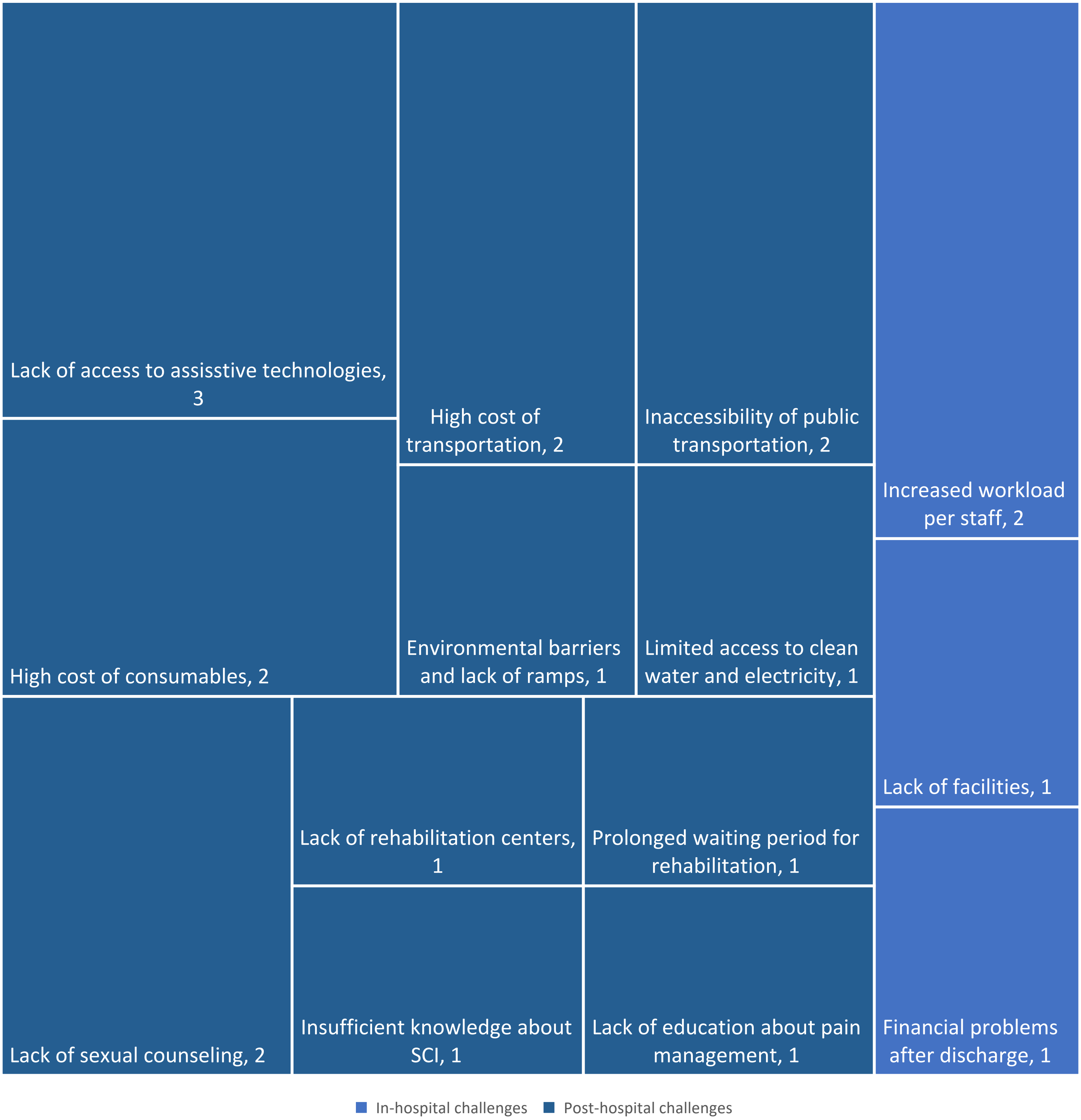

* *The numbers illustrate the number of studies that have reported each problem.*

Figure S12. Tanzania

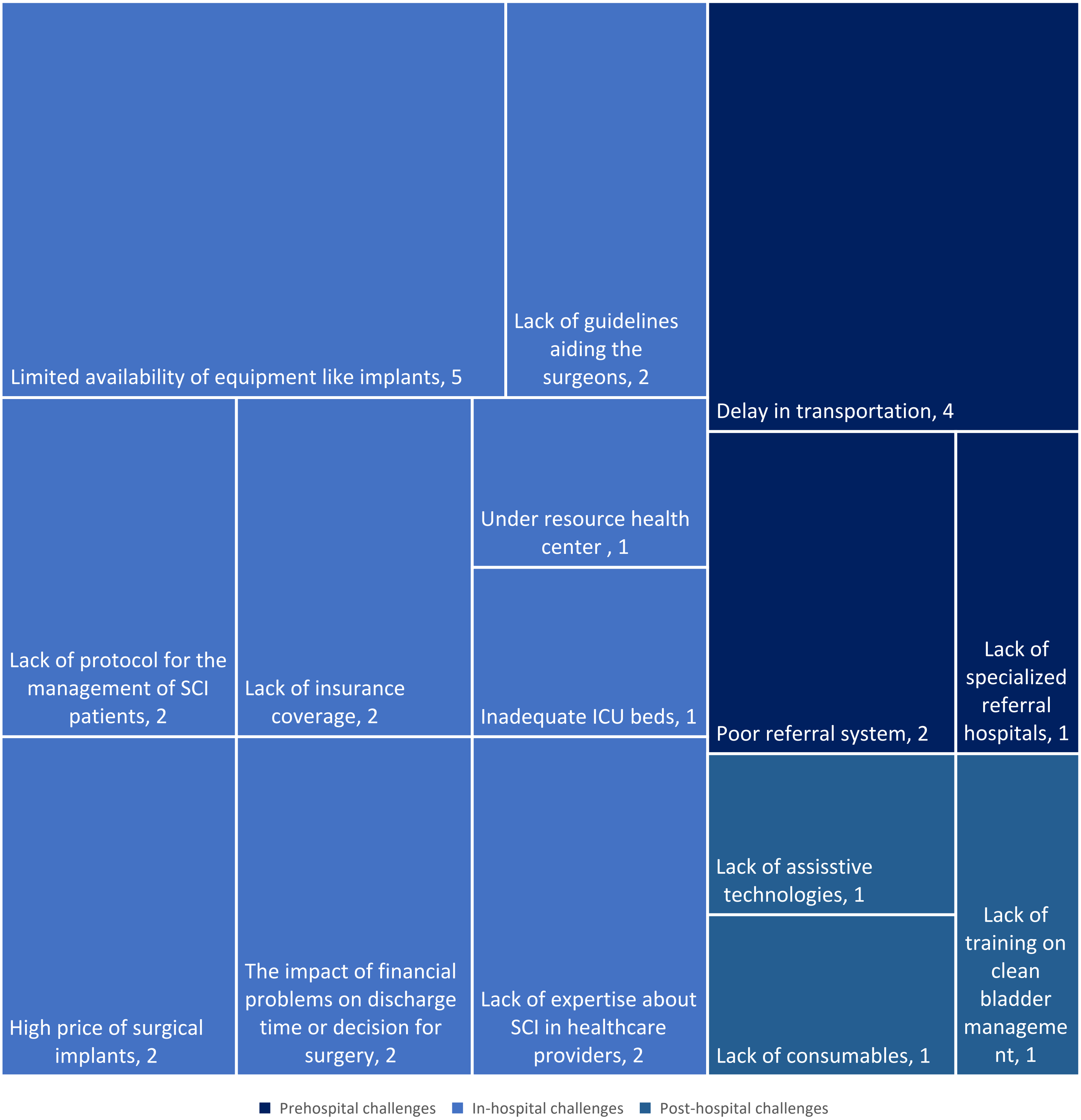

* *The numbers illustrate the number of studies that have reported each problem.*

Figure S13. Thailand

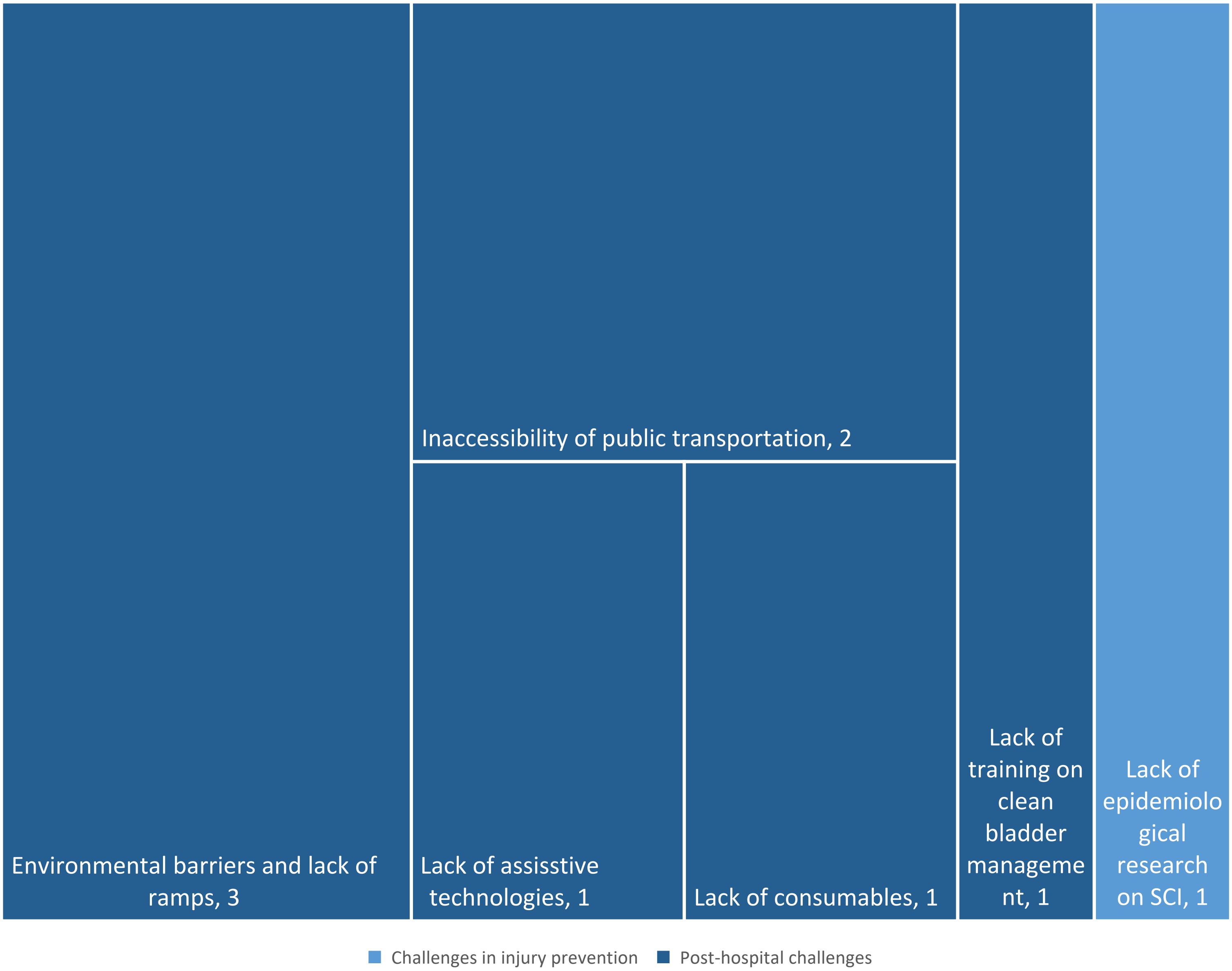

* *The numbers illustrate the number of studies that have reported each problem.*
