# Supplementary material for: Challenges in traumatic spinal cord injury care in developing countries – a scoping review
Source: Front Public Health. 2024 Aug 19;12:1377513. doi: 10.3389/fpubh.2024.1377513 (PMC11368135; doi:10.3389/fpubh.2024.1377513)
Supplement: Supplementary file 4 [file Data_Sheet_4.docx]

# Appendix D- Health Care Entity Relationships and Investments

| ***Entity*** | ***Relationship (See Legend Below)*** | ***Value*** |
| --- | --- | --- |
| Replication Medica | d |  |
| Medtronics | c |  |
| Stryker Spine | c, |  |
| Globus | c,d |  |
| Paradigm Spine | d |  |
| Stout Medical | d |  |
| Progressive Spinal Technologies | d |  |
| Advanced Spinal Intellectual Properties | d |  |
| Aesculap | c |  |
| Spine Medica | d |  |
| Computational Biodynamics | d |  |
| Spinology | d |  |
| Flagship Surgical | d |  |
| Cytonics | d |  |
| Bonovo Orthopaedics | d |  |
| Electrocore | d |  |
| Insight Therapeutics | d |  |
| FlowPharma | d |  |
| Rothman Institute and Related Properties | d |  |
| AO Spine | g |  |
| Innovative Surgical Design | d |  |
| Orthobullets | d |  |
| Thieme | c |  |
| Jaypee | c |  |
| Elseviere | c |  |
| Taylor Francis/Hodder and Stoughton | c |  |
| Expert testimony | g |  |
| Vertiflex | d |  |
| Avaz Surgical | d |  |
| Dimension Orthotics, LLC | d |  |
| SpineWave | c |  |
| Atlas Spine | c |  |
| Nuvasive | d |  |
| Parvizi Surgical Innovation | d |  |
| Franklin Bioscience | d |  |
| Deep Health | d |  |

# Legend

1. Consulting / Independent Contractor
2. Service on Scientific Advisory Board / Board of Directors / Service on Committees
3. Receipt of Royalty Payments
4. Stock / Stock Option Ownership Interests
5. Institutional / Educational Grant
6. Deputy editor/ Editor/Editorial Board
7. Member in good standing// Independent Contractor
